# Kinetics of a Ni/Ir-Photocatalyzed Coupling of ArBr with RBr: Intermediacy of ArNi^II^(L)Br and Rate/Selectivity Factors

**DOI:** 10.1021/jacs.2c06831

**Published:** 2022-08-15

**Authors:** Yael Ben-Tal, Guy C. Lloyd-Jones

**Affiliations:** EaStChem, University of Edinburgh, Joseph Black Building, David Brewster Road, Edinburgh EH9 3FJ, U.K.

## Abstract

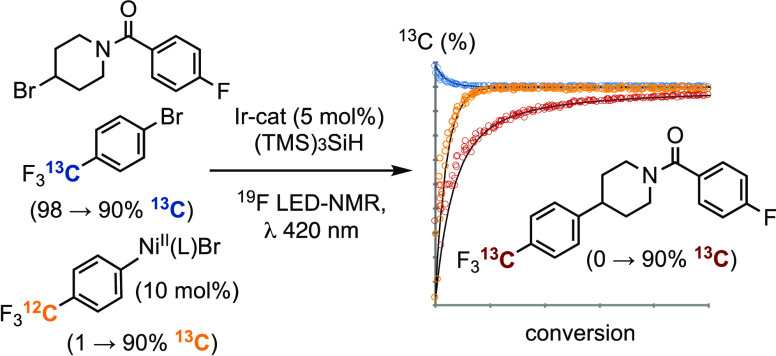

The Ni/Ir-photocatalyzed coupling of an aryl bromide (ArBr) with an alkyl bromide (RBr) has been analyzed using *in situ* LED-^19^F NMR spectroscopy. Four components (light, [ArBr], [Ni], [Ir]) are found to control the rate of ArBr consumption, but not the product selectivity, while two components ([(TMS)_3_SiH], [RBr]) independently control the product selectivity, but not the rate. A major resting state of nickel has been identified as ArNi^II^(L)Br, and ^13^C-isotopic entrainment is used to show that the complex undergoes Ir-photocatalyzed conversion to products (Ar-R, Ar-H, Ar-solvent) in competition with the release of ArBr. A range of competing absorption and quenching effects lead to complex correlations between the Ir and Ni catalyst loadings and the reaction rate. Differences in the Ir/Ni Beer–Lambert absorption profiles allow the rate to be increased by the use of a shorter-wavelength light source without compromising the selectivity. A minimal kinetic model for the process allows simulation of the reaction and provides insights for optimization of these processes in the laboratory.

## 1 Introduction

Photocatalysis is a fundamental methodology in organic synthesis. A groundbreaking advance in the application of photoredox^1^ was the independent demonstration in 2014, by Doyle and MacMillan,^2^ and by Molander,^3^ that unique reactivity could be attained by combining photocatalysis with more traditional transition metal catalysis. Most developments have been made through the combination of a photocatalyst (most often iridium-based) with a nickel cross-coupling catalyst, although other transition metals and main-group elements have also been used in place of nickel.^4−8^ This dual catalysis approach allows the union of substrate pairs wholly complementary to those coupled by more traditional second- and third-row transition metals. This complementarity has substantially expanded the chemical space accessible by synthesis, and in less than a decade since its inception, the dual catalysis principle has been applied very broadly.^9,10^

Unsurprisingly, detailed mechanistic understanding has not kept pace with the methodological advances.^11^ The situation is exacerbated by the complexity of the reactions in terms of a number of components and processes: in addition to the prerequisite catalysts, cross-coupling partners, solvent, and light, several stoichiometric additives are often also required. Nonetheless, a range of mechanistic studies, both computational^12−17^ and experimental,^17−36^ have been conducted. Most of the experimental studies consider selected components or processes within the overall reaction network. This often involves either synthesis, spectroscopy, and *in situ* studies of proposed intermediates, usually Ni species,^17−31^ or photophysical techniques probing the excited state dynamics of the photocatalyst.^30−35^ While these studies have provided valuable information about the possible behavior of the reaction, studies of the dynamics of Ni/photocatalyst systems under synthetic conditions remain rare. Indeed, to the best of our knowledge, to date, there has only been one study of the overall kinetics of dual Ni/photocatalysis: a report by Seeberger in 2020 on the acetoxylation of an aryl iodide, in which a homogeneous Ir(ppy)_3_ photocatalyst was compared with heterogeneous graphitic carbon nitride photocatalyst, using a calibrated *in situ* Fourier transform-infrared (FT-IR) method.^37^ A recent study by Kleij and co-workers of an organophotocatalyst/Co-catalyzed decarboxylative allylation also incorporated some bulk *in situ* FT-IR and ultraviolet–visible (UV-vis) kinetic measurements and modeling.^38^

Mechanistic studies on Ir/Ni-catalyzed C–C bond-forming reactions have primarily focused on the α-C-H functionalization of ethers, with pioneering contributions by Doyle,^17−20^ Wu,^39^ Molander,^21^ Martin,^26^ and König.^40^ Herein, we report on the kinetics of a closely related alkyl–aryl (sp^2^-sp^3^) coupling developed by MacMillan,^41^ a process that is of considerable utility in discovery chemistry. The investigation has allowed us to identify which of the eight reaction components control the rate, which control the selectivity, and the major on-cycle resting state of the nickel as ArNi^II^(L)X. We also report the development of a minimal kinetic model that can successfully simulate the full reaction evolution starting from a wide range of initial conditions and predict the concentrations required for high selectivity. Overall, the analysis eliminates several generic mechanisms from consideration and guides optimization in the laboratory.

## 2 Results and Discussion

### 2.1 Process Selection

**Scheme 1:**
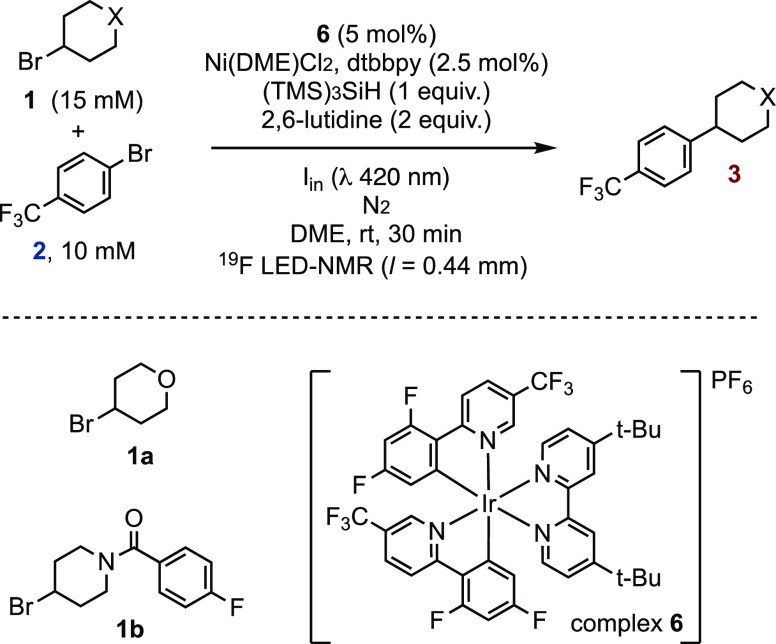
*In Situ* Analysis of a MacMillan^41^ Cross-Coupling I_in_ = monochromatic photon flux, 1.1 mEL^–1^s^–1^, and *l* is the pathlength.

Our investigation focuses on the kinetics of Ir/Ni-catalyzed coupling of secondary alkyl bromides **1a,b** with aryl bromide **2**, in the presence of (TMS)_3_SiH^42^ and 2,6-lutidine, to generate the alkyl–aryl coupling products **3a,b**, Scheme 1. To analyze the process, we constructed a software-controlled LED-NMR system^43^ (see Section S.1 in the Supporting Information) and then monitored reactions by *in situ*^19^F NMR spectroscopy.^44^ The use of 2,6-lutidine as a base ensured the homogeneity required for the analysis. The process was carefully optimized to facilitate comparison of systematic variations from a central reference point, Scheme 1, in all components, including the light intensity (photon flux, I_in_/mEL^–1^s ^–1^) and wavelength (λ). The short pathlength (*l*, 0.44 mm) and low catalyst concentrations ensure that I_in_ is approximately constant throughout the reaction volume; see Section S.5.3 in the Supporting Information.

### 2.2 Initial Observations

**Figure 1:**
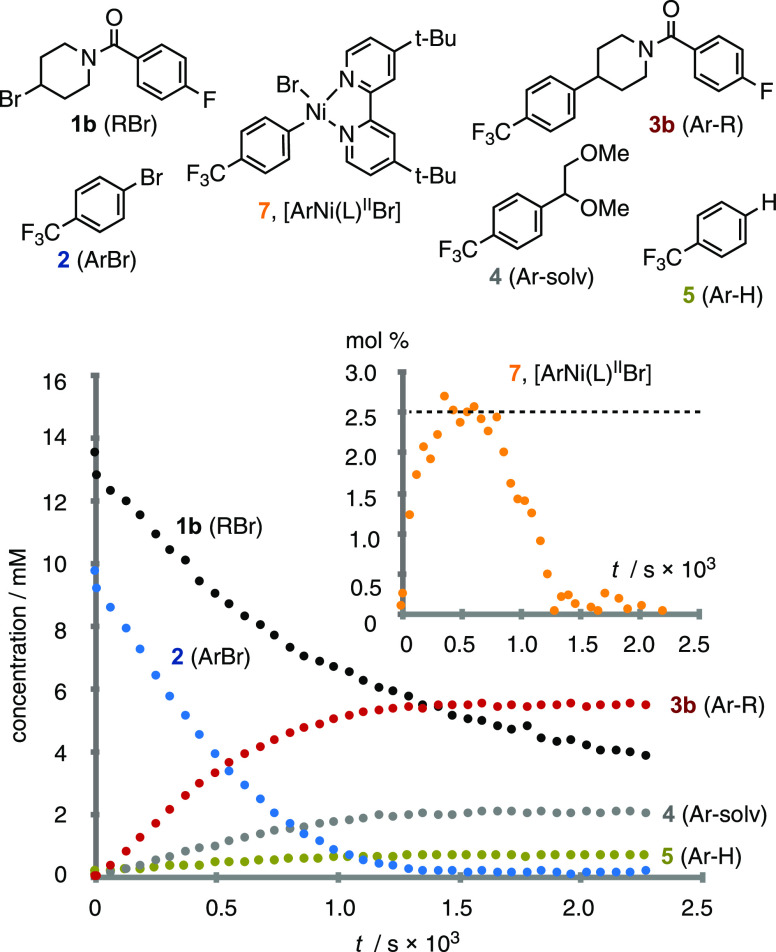
Typical profile for the reaction of **1b** and **2** under the conditions of Scheme 1. Solvent-coupled product **4** is generated as two regioisomers (ratio 7/1); only the major isomer is shown in the scheme. Inset shows profile for complex **7**, L = dtbbpy; the dashed line indicated the maximum theoretical concentration (2.5 mol%, Scheme 1). When the photon flux (I_in_) is alternated between 0 and 1.1 mEL^–1^s^–1^ (not shown), catalysis only proceeds during irradiation, and the concentration of intermediate **7** is unaffected.

Under the conditions of Scheme 1, the reactions of the alkyl bromides **1a**/**1b** with aryl bromide **2** proceed as expected to generate the alkyl–aryl coupling products **3a**,**b**, see *e.g.*, Figure 1. There are two major aryl bromide-derived side products, arising from solvent coupling (**4**, confirmed by independent synthesis) and protodebromination (**5**), with the selectivity profile (**3**, **4**, **5**) dependent on the alkyl bromide identity (**1a** versus **1b**). Several other minor side products were observed, including the protodebromination of alkyl bromide **1b** and traces of the biaryl corresponding to homocoupling of **2**, as confirmed by reference samples; see Section S.3.2 in the Supporting Information.

Under the conditions of Scheme 1, in the absence of either of the catalysts or the light, there was no consumption of **1** or **2**. In the absence of (TMS)_3_SiH, the aryl bromide **2** was coupled with the solvent (→**4**), and in the absence of alkyl bromide **1**, the aryl bromide was again converted to **4**, plus the protodebrominated product (**5**), and a broad range of minor unidentified products. While the productive coupling (**1** + **2** → **3**) only requires six of the seven components to proceed (**1**, **2**, Ir, Ni, (TMS)_3_SiH, and light), the reactions stall in the absence of base; see Section S.3.3 in the Supporting Information. We discuss the kinetics and selectivity in Sections 2.5 and 2.6.

### 2.3 Identification of an ArNi^II^ Intermediate

During these initial kinetic studies, an intermediate was detected (see inset to Figure 1). The species is formed upon irradiation of the initial reaction mixture, reaches a steady-state concentration, and then decays as the aryl bromide **2** becomes depleted. Once formed, the intermediate persists in the absence of light but decays rapidly on exposure of the solution to atmospheric oxygen. Based on the ^19^F NMR chemical shift of the CF_3_ group present in the intermediate and its steady-state concentration dependency on the quantity of the Ni(dtbbpy)Cl_2_ precatalyst employed, we identified the structure as the known ArNi^II^ complex **7**.^25^ This was confirmed by independent synthesis of **7** from Ni(COD)_2_,^25^ then *in situ* analysis after glovebox addition to a running reaction.

### 2.4 Assessing the Productivity of ArNi^II^ Complex **7**

The air- and moisture-sensitive complex **7** underwent reaction with alkyl bromide **1a**,**b** on irradiation at 420 nm in the presence of photocatalyst **6**, base, and silane to generate the coupling products **3a,b** and associated side products. In the absence of light, there was no reaction. Complex **7** (2.5 mol%) also catalyzed the reactions of **1a,b** with **2** to generate products **3**, **4**, and **5** with the same selectivity as the standard process, Scheme 1; see Section S.3.4 in the Supporting Information. Some of the prior mechanistic proposals for nickel/photoredox feature ArNi^II^ species analogous to **7** as intermediates;^16,18−20,22,26,37,45^ although more recently, catalysis via a low concentration Ni^I^/Ni^III^ cycle^23,25,28,30,31,46^ with the ArNi^II^ acting as an off-cycle ‘reservoir’ has been favored. To test this possibility, we synthesized 1-bromo-4-[^13^CF_3_]-benzene ([^13^CF_3_]-**2**) and employed this in ‘isotope entrainment’^44^ experiments, *vide infra*. The remote site of the ^13^C label ensures that there are negligible kinetic isotope effects in the Ir/Ni-catalyzed coupling reaction. [^13^CF_3_]-**2** is readily distinguished from **2** by the isotope shift (Δδ_F_ = 0.13 ppm) and scalar coupling (^1^*J*_CF_ = 272 Hz) evident in the ^19^F NMR spectrum. Analogous differences (Δδ and ^1^*J*_CF_) arise in all intermediates and products derived from [^13^CF_3_]-**2**, allowing their provenance to be traced throughout the coupling process.

**Figure 2:**
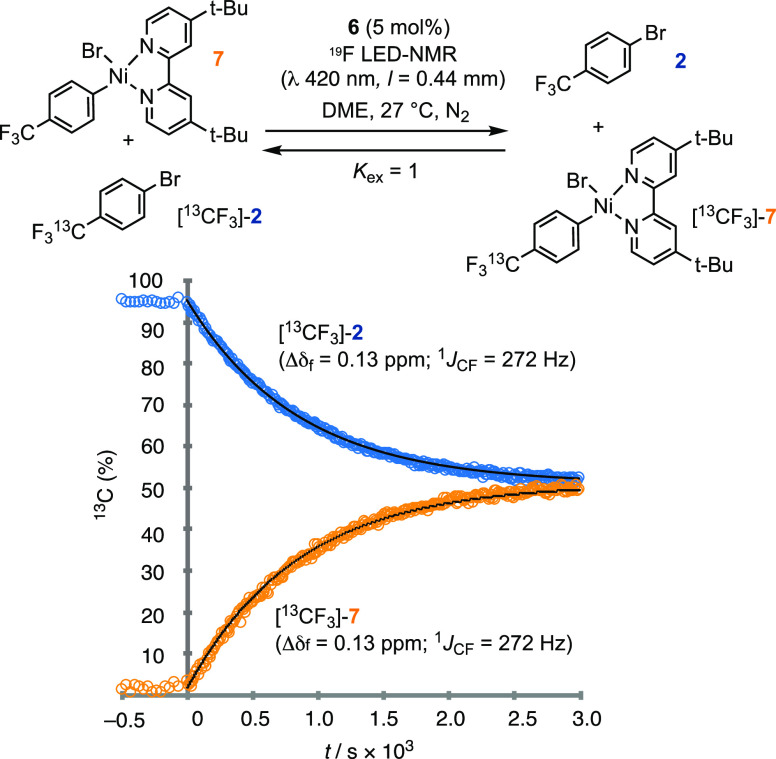
^19^F/^13^C-detected, photochemically induced, apparent exchange of the aryl groups between **7** and **2** upon low-power (I_in_ = 0.1 mEL^–1^s^–1^) irradiation at 420 nm in DME in the presence of 5 mol% Ir catalyst **6**. Under these conditions, the apparent rate coefficient for *formal* exchange ([^13^CF_3_]-**2** + **7** = **2** + [^13^CF_3_]-**7**), *k*_ex_, is 0.1 M^–1^s^–1^, see solid line through data points. Approximately 5% [^13^CF_3_]-**4** and 5% **4** are also generated.

**Figure 3:**
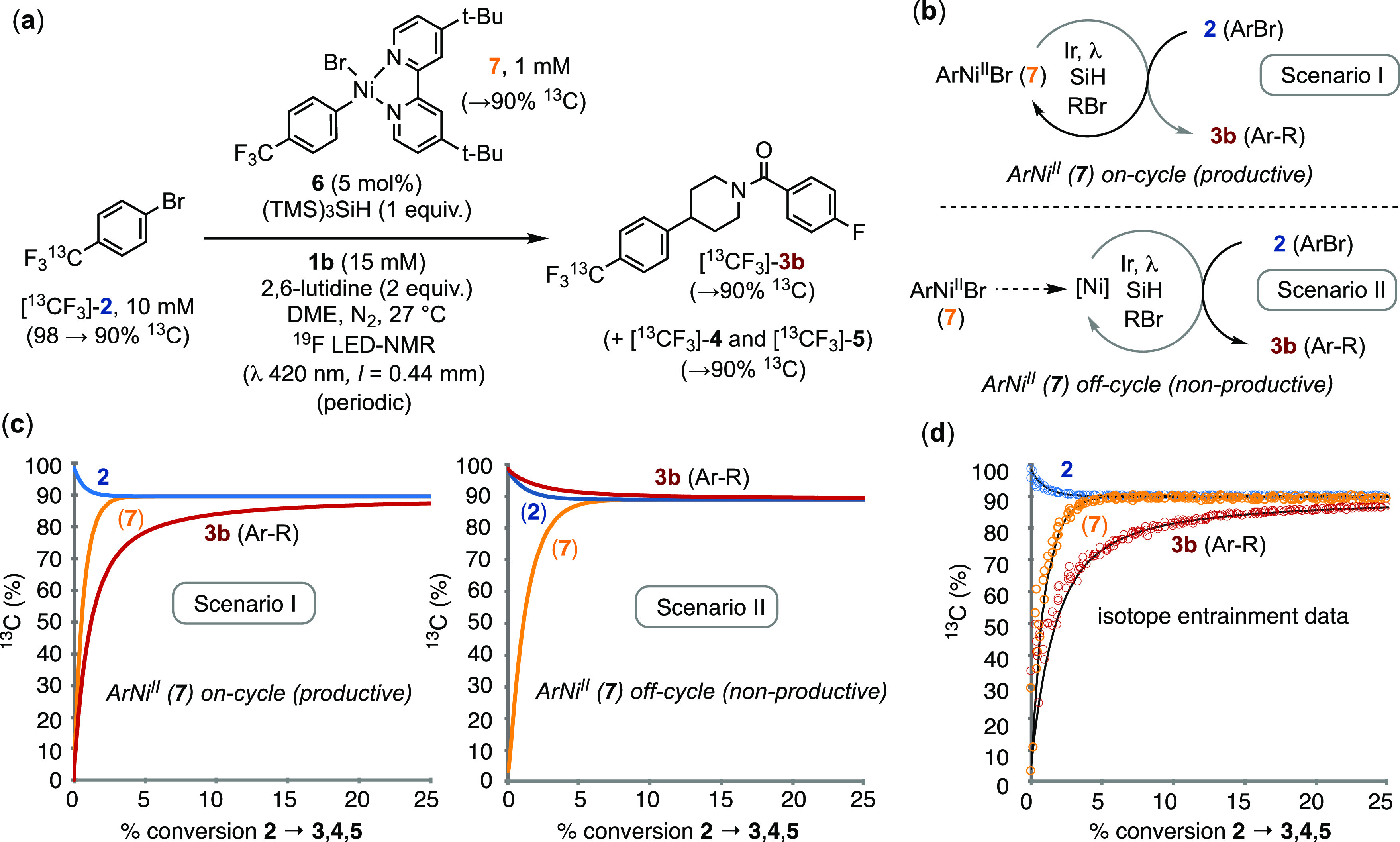
(a) Isotope entrainment to analyze the productivity of intermediate **7**; (b) generic scenarios I and II for interpretation of the isotope entrainment experiments. In scenario I, the catalytic flux occurs directly through complex **7**. In scenario II, complex **7** acts as a reservoir, with the catalytic flux occurring through reaction of the bulk ArBr mediated by *in situ*-released Ni species. (c) Examples of ^13^C incorporations under scenarios I and II when the relative rate of exchange between {[^13^CF_3_]-**2** + **7**} and {**2** + [^13^CF_3_]-**7**} is set to be in excess of that of conversion. (d) Experimental data (circles) from conditions shown in part (a), together with scenario I modeled (lines) when exchange occurs at 9.3 times the rate of conversion. The [^13^CF_3_] incorporation in the low concentrations of side products **4** and **5** mirror that in **3a**; see Section S.3.6 in the Supporting Information.

With [^13^CF_3_]-**2** in hand, we first evaluated whether the addition of **2** to Ni to generate the ArNi^II^Br complex 7 is reversible. In solution, a very slow exchange of the Ar groups between [^13^CF_3_]-**2** and **7** was detected, with equilibrium attained after 3 days. The rate was unaffected by irradiation at 420 nm or the presence of catalytic Ir complex **6** in the absence of light. However, rapid exchange was detected while the latter system was irradiated, with exchange ceasing immediately after the irradiation was paused. By attenuating the light intensity, the equilibration kinetics were readily analyzed, Figure 2. While some Ar–Ni complexes are known to be photoactive,^17,19,46−48^ this specific exchange phenomenon (Figure 2) has, to the best of our knowledge, not been detected before. It has important consequences for the kinetic analysis, *vide infra*, and results in an inherent photochemical inefficiency in the coupling, Scheme 1. Having quantified the kinetics of the nonproductive Ir-photocatalyzed exchange of {[^13^CF_3_]-**2** + **7**} with {[^13^CF_3_]-**7** + **2**}, we were then able to interpret the outcome of isotope entrainment experiments under the coupling conditions, Figure 3.

Irradiation of a solution containing [^13^CF_3_]-**2**, 5 mol% Ir-photocatalyst **6**, 11 mol% **7**, alkyl bromide **1b**, (TMS)_3_SiH, and 2,6-lutidine led to turnover to generate the usual mixture of aryl-derived coupling products (**3**–**5**), but now containing ^13^CF_3_ in various proportions, Figure 3a. To interrogate the system, we employed a ‘periodic activation’^44^ approach in which the 420 nm irradiation (I_in_) was alternated between 0.05 mEL^–1^s^–1^, during which the system evolved, and 0 mEL^–1^s^–1^, during which high-quality ^19^F NMR were acquired. This allowed the [^13^CF_3_]-populations in substrate (**2**), ArNi^II^ complex **7**, and products (**3**, **4**, **5**) to be reliably analyzed as a function of conversion.

The results can be interpreted by consideration of the two limiting scenarios (I and II) presented in Figure 3b. In scenario I, complex **7** is a genuine intermediate, and each revolution of the cycle transfers the Ar group in **7** into the coupled product **3b**. In the absence of any competing Ir-photocatalyzed exchange of [^13^CF_3_]-**2** and **7**, the first turnover generates **3b** and subsequent cycles generate [^13^CF_3_]-**3b**. In scenario II, complex **7** is off-cycle and acts as a reservoir for the release of low concentrations of highly active Ni species that perform the productive catalysis and convert **1b** + [^13^CF_3_]-**2** into [^13^CF_3_]-**3b**. In both scenarios, the competing Ir-photocatalyzed exchange of [^13^CF_3_]-**2** and **7** (as in Figure 2) results in release of **2** and generation of [^13^CF_3_]-**7**. The rate of the exchange versus turnover governs the theoretical profile of [^13^CF_3_] incorporation (%) versus fractional conversion. In Figure 3c, the two scenarios have been modeled when the ratio of exchange is one order of magnitude faster than turnover.

Analysis of the experimental data, Figure 3d, shows that the cross-coupling is sufficiently competitive with the rate of exchange of [^13^CF_3_]-**2** and **7** to identify that complex **7** is an active intermediate in the generation of **3b**. Kinetic simulation of scenario I gave a good fit (see lines through the data points) when the rate ratio for exchange/coupling was set at 9.3. Moreover, the [^13^CF_3_] incorporations in **4** and **5** mirror those in **3b**, and thus all three species (**3**,**4**,**5**) emanate from the same general catalytic flux. Analogous isotope entrainment experiments in which [^13^CF_3_]-**2** (0.5 equiv) was added to an evolving coupling of **1b** with **2** (0.5 equiv) under the standard conditions, Scheme 1, using the Ni(dtbbpy)Cl_2_ precatalyst also gave results consistent with catalytic flux predominantly or exclusively involving the ArNi^II^ complex **7**; see the Supporting Information S.3.6.

### 2.5 Rate and Selectivity Sequence

**Figure 4:**
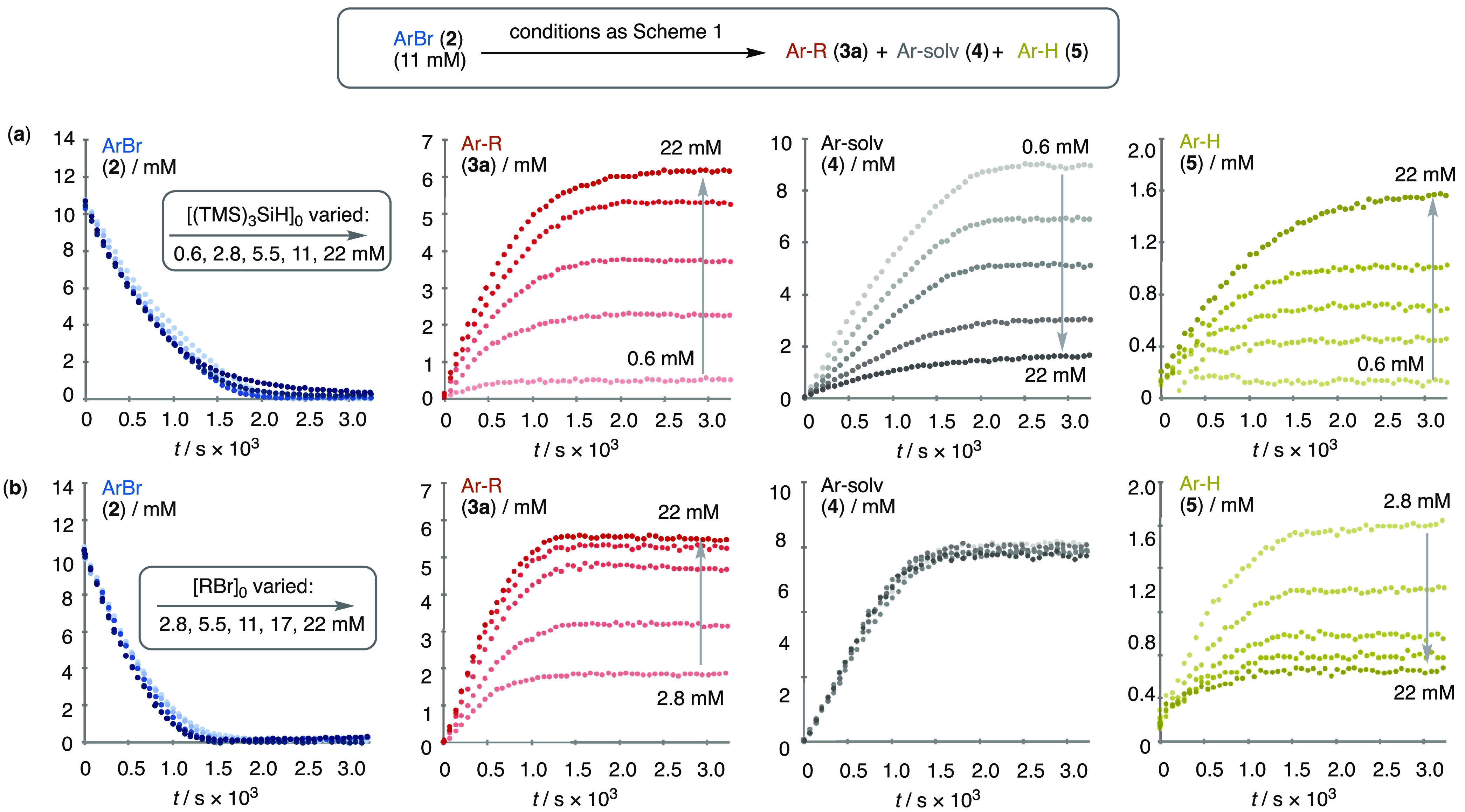
Impact of (a) (TMS)_3_SiH and (b) RBr concentrations on the rate and selectivity of coupling. See the Supporting Information S.3.7 for analyses of the effects of light intensity and the concentrations of [ArBr], [Ni], [Ir], and [2,6-lutidine].

Using the conditions of Scheme 1 as a central reference point, we then determined the full reaction profiles for the coupling of **1a** with **2**, under more than 40 different sets of initial conditions, in which all seven components were systematically varied. Although the initial rates provided some preliminary insights—for example, product evolution corresponds linearly to light intensity—the overall system was too complex for this approach to enable holistic evaluation. Instead, we used visual kinetic analysis to gain preliminary insight into the factors controlling the rate, selectivity, and speciation. This led to the testing of steady-state approximations and, ultimately, the construction of a minimal model for numerical method simulation (Section 2.6).^44^ The complete sets of data are detailed in section S.3.7 of the Supporting Information. Here, for illustration, we present just two examples. In Figure 4a, [(TMS)_3_SiH]_0_ is the variable, ranging from 0.6 to 22 mM, and in Figure 4b, alkyl bromide ([**1a**]_0_) is the variable, ranging from 2.8 to 22 mM.

In both datasets, the analysis shows that the rate of consumption of aryl bromide **2** is unaffected by either the (TMS)_3_SiH or the alkyl bromide (**1a**) concentration. The partitioning of the aryl-derived products (**3a**/**4**/**5**), which occurs after the turnover rate-limiting event, is, however, sensitive to the (TMS)_3_SiH and RBr (**1a**) concentrations, but in differing ways. In the case of (TMS)_3_SiH, raised concentrations favor generation of both the cross-coupled (**3a**) and protodebrominated (**5**) products, by attenuating the solvent coupling (**4**), Figure 4a. In contrast, when the alkyl bromide **1a** is varied, the partitioning of aryl bromide into the solvent-coupled product **4** is unaffected (Figure 4b), and instead, raised concentrations of **1a** favor generation of the cross-coupled (**3a**) by attenuating the protodebromination (**5**); see Section S.3.7 of the Supporting Information.

**Figure 5:**
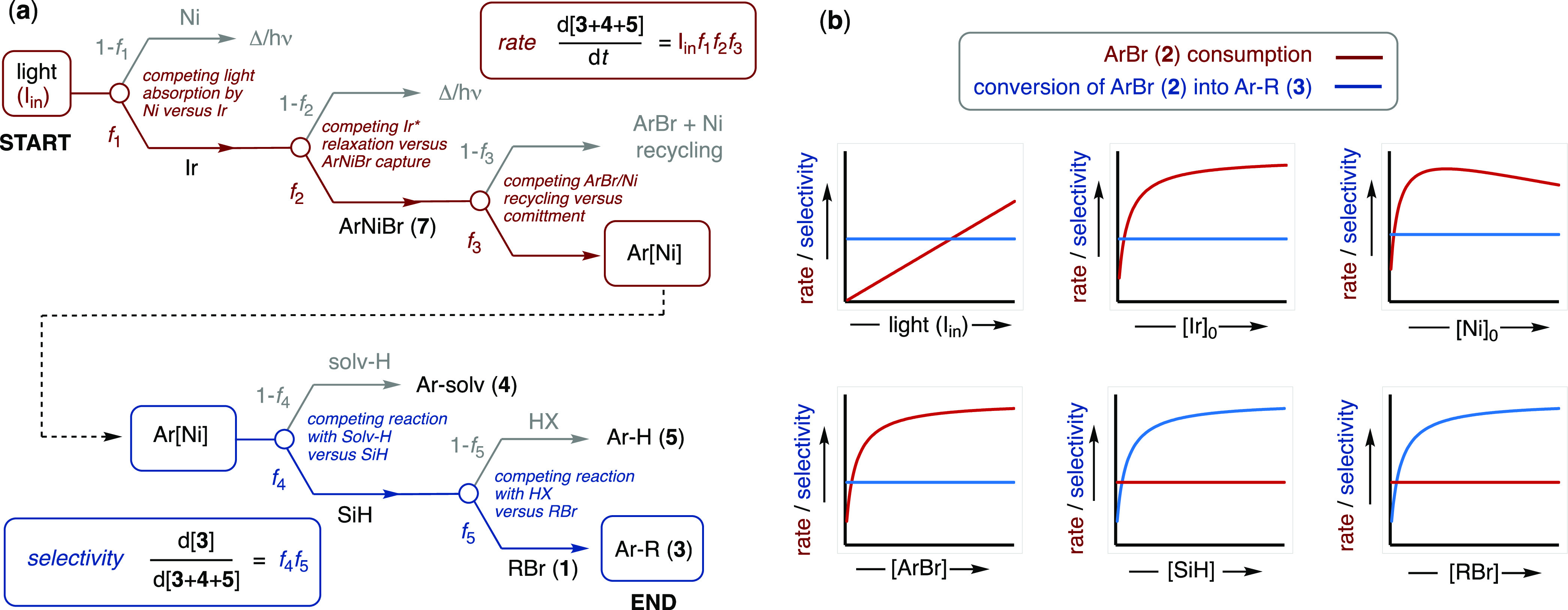
(a) Flow diagrams depicting two schematic sequences of fractionations established by analysis of the influence of all components on the rate (*f*_1_·*f*_2_·*f*_3_) and selectivity (*f*_4_·*f*_5_) of conversion of photon flux (I_**in**_; EL^–1^s ^–1^) and ArNiBr (**7**) into coupling product Ar–R (**3**). Δ/h*v* = heat, unabsorbed light, fluorescence, *etc*. (b) Schematic illustration of the effect of the variables on the rate and selectivity at moderate photon flux (I_**in**_) over short pathlengths. The kinetic data obtained indicate that additional Ir- and Ni-mediated quenching processes attenuate the rate, see Section S5.3 in the Supporting Information, possibly by attenuation of *f*_2_ and *f*_3_.

Analogous investigation of the effects of light intensity (I_in_; mEL^–1^s^–1^), concentrations of [ArBr], [Ni], [Ir], and [2,6-lutidine], and alkyl halide identity (**1a**/**b**) allowed deduction of the two sequences shown in Figure 5a. Although small changes in the chemical shift of **6**, consistent with anion metathesis (PF_6_/Br), were detected during the reaction, there was no apparent influence of endogenous or exogenous bromide on the rate or selectivity;^49^ see the Supporting Information S.9. The flow diagram shows five discrete events that control the overall rate of Ar–R product generation, d[**3**]/d*t* (Ms^–1^) based on a series fractionations (*f*_1_ → *f*_5_) beginning with the photon flux (I_in_ / EL^–1^s^–1^).

The flow diagram comprises two sections: {I_in_·*f*_1_·*f*_2_·*f*_3_} controls the *rate* of consumption of the ArBr (**2**) and {*f*_4_·*f*_5_} controls the *selectivity* for its conversion to the coupling product Ar–R (**3**). Fractionations *f*_1_ and *f*_2_ govern the efficiency of conversion of the photon flux (I_in_; EL^–1^s^–1^) into Ni turnover. Fractionation *f*_3_ relates to the commitment of NiArBr (**7**) to product generation versus recycling of ArBr, *vide infra.* Fractionations *f*_4_ and *f*_5_ govern the efficiency of conversion of the committed Ar substrate (i.e., that emerging from I_in_·*f*_1_·*f*_2_·*f*_3_) into the aryl/alkyl coupling product Ar–R (**3**).

The selectivity is determined by a specific sequence, first is *f*_4_, which is [(TMS)_3_SiH]-dependent, then *f*_5_, which is [RBr, **1**]-dependent. Fractionations *f*_1_ → *f*_4_ are thus independent of the alkyl bromide identity (**1a**,**b**); see Section S.3.8 in the Supporting Information. 2,6-Lutidine serves to inhibit stalling of the Ni/Ir-catalyzed reaction but does not directly control the rate or the selectivity. Under the conditions explored, the overall rate of aryl/alkyl cross-electrophile coupling (d[**3**]/d*t*; eq 1) corresponds to the product of two terms: the rate of consumption of ArBr (**2**), (I_in_*·f*_1_*·f*_2_*·f*_3_), and the selectivity of its conversion into Ar–R (**3**) (*f*_4_*·f*_5_); see Figure 5 (a) and eqs 2 to 6.
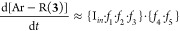
1
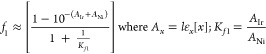
2
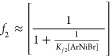
3
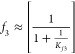
4
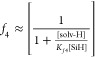
5
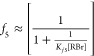
6

Equation 2 comprises Beer–Lambert components (*A*_x_) to account for Ir versus Ni fractionation in the capture of the photon flux, I_in_; EL^–1^s^–1^ over pathlength *l* /cm. The opposing influence of [Ni] in *f*_1_ and *f*_2_, together with additional quenching processes by Ir and Ni, see Section S5.3 in the Supporting Information, results in complex behavior of the rate, *but not the selectivity*, with respect to the catalyst concentrations, Figure 5b. Saturation in [ArBr], and thus possibly also [ArNiBr], was observed by Seeberger in C–O cross-coupling.^37^Eq 4 corresponds to the fractional commitment (*f*_3_) of ArNiBr to the generation of products (**3**,**4**,**5**) versus ejection of ArBr, as detected by the isotope entrainment analysis, Figure 3.

The rate of consumption of ArBr (**2**) is independent of the [(TMS)_3_SiH] and [RBr] concentrations, as these impact on the fractionations occurring after commitment (*f*_3_) to product generation, Figure 5a. The [(TMS)_3_SiH] and [RBr] concentrations do, however, independently govern the selectivity for aryl/alkyl cross-electrophile coupling (Ar–R, **3**) over solvent coupling (**4**) and protodebromination (**5**). Equations 5 and 6 indicate how the selectivity becomes independent of both [(TMS)_3_SiH] and [RBr] at high concentrations. Evidence for the direct participation of the solvent (solv-H = DME) in fractionation step *f*_4_ includes an increase in selectivity for **3** + **5** over **4** in *d*_10_-DME, corresponding to *K*_*f*4(D)_/*K*_*f*4(H)_ ≈ 2; see Section S.3.9 in the Supporting Information.

On changing the *in situ* LED-NMR irradiation wavelength from 420 to 455 nm, the intensity-normalized rate of product generation under the standard conditions, Scheme 1, became significantly slower. However, the selectivity profiles (**3**, **4**, **5**) were the same, indicative that the photon flux, I_in_, is not involved in *f*_4_ or *f*_5_ of the fractionation sequence. The experimentally determined extinction coefficients (ε/M^–1^ cm^–1^) of **6** and **7** at 455 nm (ε_Ir_ = 0.4 × 10^3^; ε_Ni_ = 2.9 × 10^3^) versus 420 nm (ε_Ir_ = 2.2 × 10^3^; ε_Ni_ = 2.7 × 10^3^) predict a 6-fold decrease in *f*_1_, eq 2, at 455 nm compared to 420 nm. This is consistent with an experimentally determined 7-fold reduction in the rate at 455 nm; see Sections S.3.3.1 and S7 in the Supporting Information.

### 2.6 Development of a Kinetic Model

**Figure 6:**
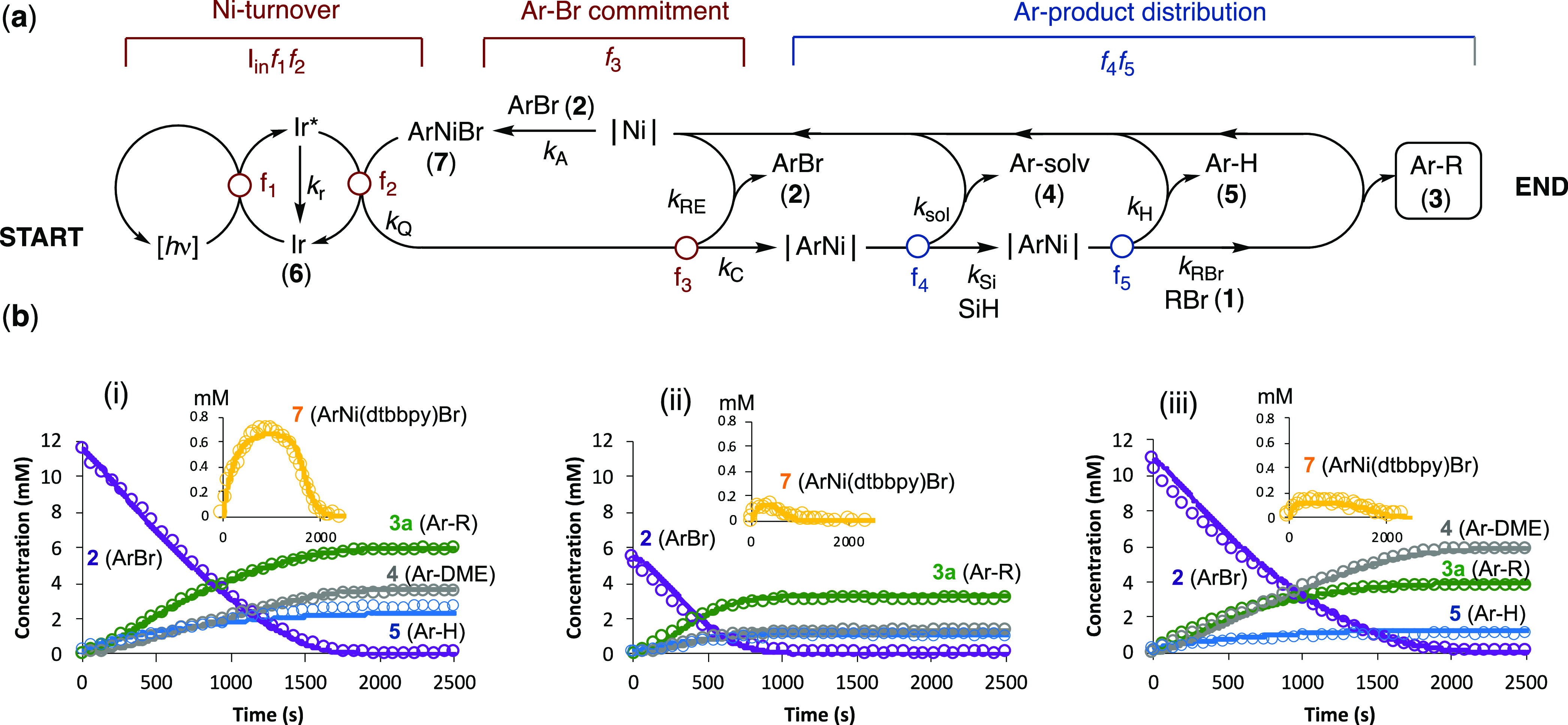
(a) Minimal kinetic model for simulation of Ir/Ni-catalyzed conversion of ArBr + RBr + photon flux (I_**in**_) into coupling product Ar–R (**3**) and side products Ar–solv (**4**) and Ar–H (**5**), under the conditions explored, Scheme 1. (b) Three examples (from 35) of correlations of experimental data for the reaction of **1a** with **2** (open circles; determined by *in situ* LED-^19^F NMR spectroscopy) with simulations using the minimal model (lines through data). The model is predominantly ‘*chemically agnostic’* and includes an induction process (not shown) that converts NiX_2_ into the on-cycle species |Ni| and *a* progressive decay in Ir. The five fractionation points indicated (*f*_1_ → *f*_5_) correspond to the sequence established by semiquantitative analysis, Figures 4 and 5. A kinetically indistinguishable alternative involves fractionation *f*_2_ giving two discrete Ar–Ni species. See section S.5.2 in the Supporting Information for all 35 datasets, discussion of the model, simulation parameters, and the impacts of pathlength and Ir/Ni concentrations. Initial conditions for the reactions, listed for i, ii, iii: [**1a**] = 15, 15, 13, mM; [**2**] = 12, 5, 11, mM; [**6**] = 0.64, 0.52, 0.56, mM; [Ni(dme)Cl_2_] and [dtbbpy] = 0.77, 0.26, 0.16; [(TMS)_3_SiH] = 11, 12, 6, mM; [2,6-lutidine] = 20, 20, 20, mM; λ (420 nm) = 0.31, 1.1, 1.1, mEL^–1^ s^–1^; and *l* = 0.044 cm.

Having established the sequences and components that control the rate and selectivity, Figure 5, we conducted kinetic simulations of the process, starting from 35 different initial conditions; see the Supporting Information S.5. The simulations must account for the rate of consumption of ArBr (**2**), the steady-state concentration of Ar–Ni^II^ complex **7**, the Ir-photocatalyzed exchange of the aryl groups between **2** and **7**, and the selectivity for **3** over side products **4** and **5**. After many iterations, we established the model shown in Figure 6. While the model depicts the Ar–Ni^II^ complex **7**, *in all other aspects, by design, it is ‘chemically agnostic’*’: it solely serves to semiquantitatively describe the rate and selectivity in minimal complexity. For discussion of other arrangements and kinetic sequences that were considered less reasonable; see Section S.6.2 in the Supporting Information.

The model comprises three interlinked stages (i) photochemically driven turnover of coupled Ir–Ni cycles, (ii) Ar–Br commitment via Ar–Ni complex **7**, and (iii) determination of the Ar product selectivity; see Figure 6. In the first stage, the Ir is excited and either relaxes (*k*_r_) or is quenched (*k*_Q_) by Ar–Ni complex **7**. Rather than photon flux, the simulation employs a notional chemical species ‘h*v*’ as a catalyst for Ir excitation, with the concentration [h*v*] being modulated to account for the competing Beer–Lambert absoprtion behavior of [Ni]; see Section S.5.3 in the Supporting Information. In the second stage, the Ar group is either recycled back into aryl bromide (*k*_RE_ → **2**) or committed (*k*_C_ → |Ar-Ni|) to the third and final stage. The selectivity is determined in two independent sequential fractionations, *f*_4_ and *f*_5_, both of which occur after the turnover rate-limiting events in the first and second stages. The selectivity in fractionations *f*_4_ and *f*_5_ progressively reduces with conversion through depletion of the (TMS)_3_SiH and RBr concentrations. End-point ^1^H and ^29^Si NMR analyses of the couplings indicate that (TMS)_3_SiH is converted to (TMS)_3_SiBr; see Section S.3.3.10 in the Supporting Information; however, the insensitivity of the ^29^Si NMR method precluded detailed *in situ* analysis of the processes leading to this. All steps in the third and final stage (*k*_sol_, *k*_H_, and *k*_RBr_) ultimately lead to readdition (*k*_A_) of aryl bromide **2** and thus repopulation of the Ar–Ni complex **7**.

Given the complexity of the overall cross-coupling, the model gave reasonably satisfactory fits to the temporal concentration profiles for **2**, **3**, **4**, **5**, and **7** across the span of variations in all components. Three examples of these are shown in Figure 6; the fits for all 35 datasets are provided in section S.5.2 of the Supporting Information.

### 2.7 Mechanistic Insight from Kinetic Simulations

**Figure 7:**
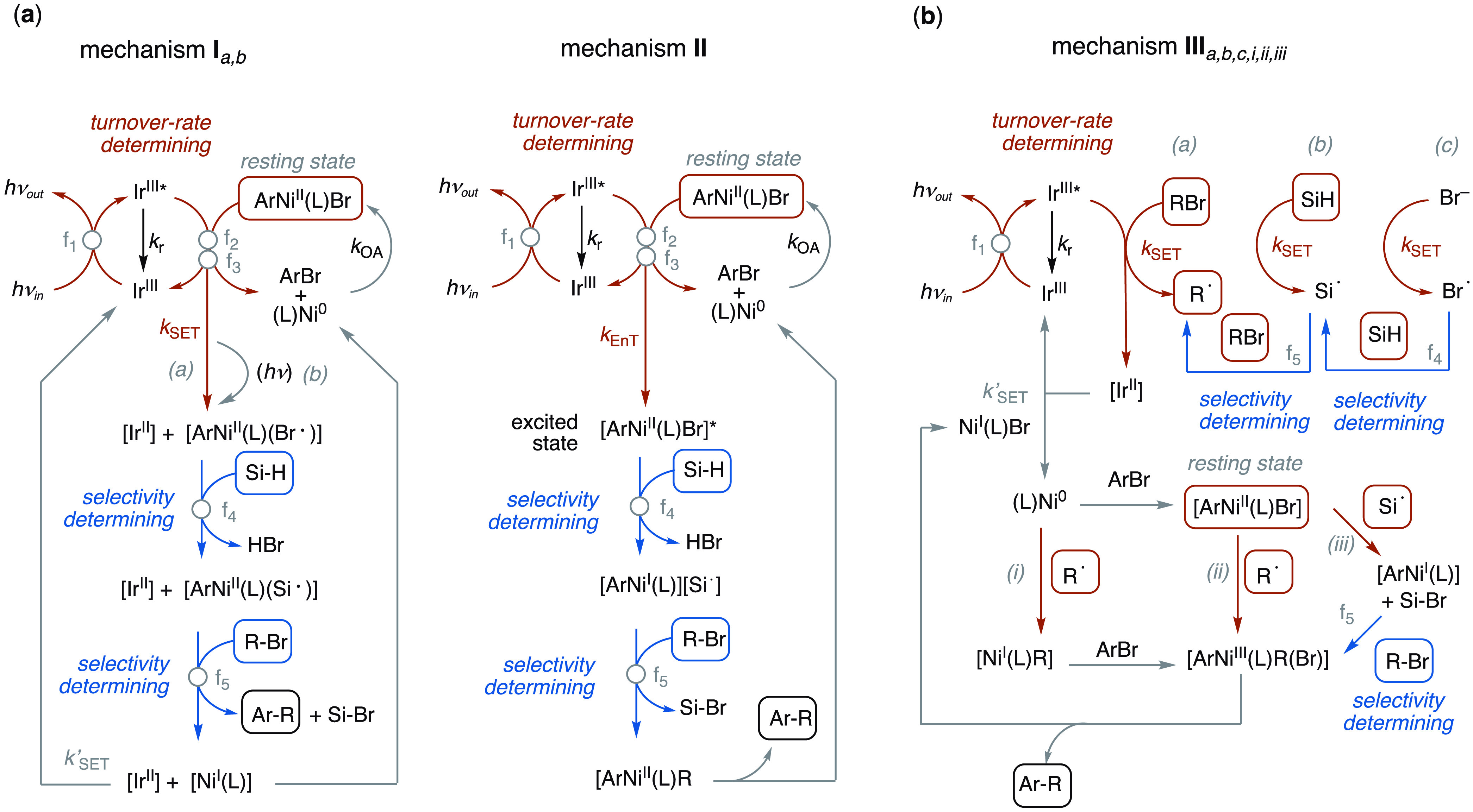
Examples of mechanisms for Ir/Ni-catalyzed ArBr + RBr coupling via an ArNi^II^(L)Br intermediate and with single electron transfer (*k*_SET_) and energy-transfer (*k*_EnT_) steps.^50^ (a) Mechanisms **I** and **II** with which the minimal model (Figure 6; see also section S.5 in the Supporting Information) can be readily reconciled. (**b**) Mechanism **III**, for which we were unable to configure any models that displayed overall kinetic behavior phenomenologically consistent with that found empirically; see Section S.6 in the Supporting Information.

The cross-coupling in Scheme 1 requires the input of eight reaction components (ArBr, RBr, Ir, Ni, (TMS)_3_SiH, light, base, solvent) and their manifold interconnection through a complex network of chemical, photophysical, and physicochemical processes. A complete mechanistic analysis of the reaction is substantially beyond the scope of this study, and we have been deliberately ‘agnostic’ about many aspects of the speciation. Nonetheless, the kinetic behavior that has been elucidated (Figures 5 and 6, eqs 1–6, and sections S.3.7 and S.5.2 of the Supporting Information) does provide valuable constraints when considering the wide range of mechanisms that have been proposed for these and related couplings.^50^ Below, we consider examples of generic mechanisms that feature the requisite ArNi^II^(L)Br complex; **I**, **II**, **III**, Figure 7.

The general kinetic behavior of the coupling (Scheme 1) can be satisfactorily simulated using a minimal model (Section 2.6) that is readily reconciled with mechanisms **I** and **II** (Figure 7) under most conditions. The key distinction between them is that mechanism **I** involves electron transfers (*k*_SET_) between Ni and Ir complexes, while mechanism **II** involves energy transfer (*k*_EnT_). This results in some differences in the speciation and oxidation states (Ni and Ir) and also in the mechanisms for (nonrate-limiting) selectivity. Prior investigations on similar systems have been unable to definitively distinguish energy transfer from electron transfer processes.^31^ In contrast to **I** and **II**, very specific conditions and constraints are required in kinetic simulations using models based on any configuration (*a*,*b*,*c*,*i*,*ii*,*iii*) of **III**. Indeed, to the best of our abilities, we were unable to find a general fit to **III**, with significant deviations between the predicted and observed kinetic behavior across the 35 datasets that were explored; see Section S6 in the Supporting Information for further discussion.

## 3 Conclusions

A dual Ir/Ni-photocatalyzed cross-electrophile coupling of alkyl bromide **1** with aryl bromide **2**, Scheme 1, has been investigated using a combination of *in situ* LED-^19^F NMR spectroscopy, ^13^CF_3_-labeling, and kinetic simulations. The major nickel speciation at steady state is ArNi^II^(L)Br complex **7**, with isotope entrainment indicative that this is an active intermediate (scenario I, Figure 3) rather than a Ni reservoir (scenario II, Figure 3). The silane ((TMS)_3_SiH) and alkyl bromide (RBr) interact independently but in a specific sequence. The silane diverts the process away from solvent arylation, then the alkyl bromide diverts the process away from protodebromination (Ar–H), Figure 5a. A simple overarching model, Figure 6, accounts for the behavior of the system. The minimal model is explicitly ‘agnostic’ on several important points of contention in the current literature, and while it can be reconciled with generic mechanisms **I** and **II**, Figure 7, *we are not suggesting these to be definitive or exclusive*. Nonetheless, the general kinetic relationships that have been elucidated provide a framework for future mechanistic work.

It is important to note that the kinetics were analyzed over a short pathlength (*l*, 0.44 mm) with low concentrations of all components. Under such conditions, simple Beer–Lambert and steady-state approximations can be applied, eqs 1 to 6; see Section S.5.3 in the Supporting Information for further discussion. In contrast, longer pathlengths and higher catalyst and reactant concentrations are routinely employed in synthesis, and this will lead to large instantaneous light-intensity gradients, local perturbations in Ir and Ni catalyst speciation, mass-transfer (diffusion) limitations, and self quenching. Nonetheless, the five key findings noted below provide insights for optimization of these and related dual photocatalysis processes in the laboratory:

(i) Four reaction components (incident photon flux (I_in_), [Ni], [Ir], and [ArBr]) control the rate of ArBr consumption but not the product (Ar-R) selectivity. Two components ([(TMS)_3_SiH] and [RBr]) control the product selectivity but not the rate. The rate and selectivity are independent of the base (2,6-lutidine) concentration, but its presence is essential to inhibit the eventual stalling of the reaction by the HBr that otherwise accumulates throughout turnover.
(ii) Under most conditions, the rate of turnover approaches concentration independence (’saturation’) in [ArBr], despite the competing Ir-photocatalyzed recycling of the steady-state ArNi^II^(L)Br complex **7** into ArBr/Ni, Figure 5a.
(iii) Selectivity for the cross-coupling product (Ar–R) is raised using excess silane and alkyl bromide. For the reaction of RBr **1a** with ArBr **2**, the selectivity is predicted to be ≥98% when [(TMS)_3_SiH] and [**1a**] are both ≥0.3 M; see Section S.3.7 in the Supporting Information.
(iv) Beer–Lambert behavior, competitive quenching, saturation, and ArBr/Ni recycling mean that changes in the Ir and Ni catalyst loadings do not necessarily translate into corresponding changes in the rate of productive turnover, Figure 5b. The behavior depends on the absolute concentrations of the catalysts, the incident light intensity, and the pathlength. Indeed, under some conditions, increases in the catalyst loadings can result in significant decreases in rate.
(v) The ArNi^II^ complex **7** competes with the Ir photocatalyst **6** for the incident light (eq 2). Complex **6** has a six-fold greater extinction coefficient (ε) at 420 nm compared to 455 nm, whereas the ε-values for complex **7** are similar at the two wavelengths. This results in the reaction proceeding faster at 420 nm than 455 nm, without loss of product selectivity. The use of a violet rather than the blue light source is thus of significant benefit in this class of coupling.

## Supporting Information Available

Experimental details; characterization data; reaction profiles; kinetic simulations; and NMR spectra (PDF)
