# Supplementary material for: Kinetics of a Ni/Ir-Photocatalyzed Coupling of ArBr with RBr: Intermediacy of ArNiII(L)Br and Rate/Selectivity Factors
Source: J Am Chem Soc. 2022 Aug 15;144(33):15372–82. doi: 10.1021/jacs.2c06831 (PMC9413222; doi:10.1021/jacs.2c06831)
Supplement: Supplementary file 1 — ja2c06831_si_001.pdf [file ja2c06831_si_001.pdf]

SUPPORTING INFORMATION

Kinetics of a Ni/Ir Photo-Catalyzed Coupling of ArBr with RBr: Intermediacy of $ArNi^{II}(L)Br$ and Rate/Selectivity Factors

Yael Ben-Tal[∝] and Guy C. Lloyd-Jones[∝]*

[∝]EaStChem, University of Edinburgh, Joseph Black Building, David Brewster Road, Edinburgh, EH9 3FJ, UK

*E-mail: guy.lloyd-jones@ed.ac.uk

Table of Contents

1. General Considerations 4
2. Synthesis 6
4-(4-(trifluoromethyl)phenyl)tetrahydro-2H-pyran,[S2] 3a 6
1-(4-trifluoromethylphenyl)-1,2-methoxyethane, 4 6
4,4′-Bis(trifluoromethyl)biphenyl S1 7
N-(4-fluorobenzoyl)-4-bromopiperidine,[S7] 1b 7
N-4-fluorobenzoylpiperidine,[S8] S2 8
(4,4′-di-tert-butyl-2,2′-bipyridine)Ni(4-trifluoromethylphenyl)(Br),[S9] 7 9
[$^{13}CF_3$]-4-bromobenzotrifluoride, [$^{13}CF_3$]-2 9
3. Kinetic Studies 11
3.1 General Procedure for *in situ* Kinetic Analyses 11
3.2 Representative temporal-concentration data 12
3.2.1 4-bromotetrahydropyran, 1a, as AlkBr 12
3.2.2 N-(4-fluorobenzoyl)-4-bromopiperidine, 1b, as AlkBr 14
3.3 Control Experiments 16
3.3.1 Comparison of reactions at 455 nm and 420 nm 16
3.3.2 No Photocatalyst 17
3.3.3 No AlkBr 18
3.3.4 No Silane 18
3.3.5 No Base 19
3.3.6 Midpoint ^{19}F NMR control reactions 19
3.3.9 Light "on-off" studies 20
3.3.10 ^{1}H and ^{29}Si NMR spectroscopic analysis of silane. 21
3.4 Reactivity of NiArBr complex 7 23
3.4.1 Stoichiometric reaction of 7 with Alkyl Bromide 1b 23
3.4.2 Use of ArNi(L)Br complex 7 as catalyst 24
3.5 Photochemically-Induced Ar-exchange between ArBr (2) and ArNi(L)Br (7) 25
3.6 Isotope Entrainment 26
3.7 Systematic Variation of Individual Components 29
3.7.1 Variation of Ni catalyst loading 30
3.7.2 Variation of Ir photocatalyst 6 loading 31
3.7.3 Variation of ArBr 2 loading 32
3.7.4 Variation of Alkyl Bromide 1a Loading 33

3.7.5 Variation of Silane Loading 34
3.7.6 Variation of Base Loading 35
3.7.7 Variation of Light Intensity 36
3.8 Comparative concentration-concentration plot showing independence of silane-mediated and Alk-Br mediated partitions..37
3.9 DME Solvent $^{1}H/^{2}H$ KIE..38
4. Determination of the Absolute Photon Flux of the LED-NMR Apparatus using Ferrioxolate Actinometry 39
5. Kinetic Simulations 42
5.1 Model Development 42
5.2 Optimisation of the Final Minimal Model (Figure S30) 44
5.2.1 Datasets, initial conditions and fitted variables. 45
5.2.2. Individual fits of datasets, numbering as in Table S2. 46
5.3 General considerations of the Impact of Beer-Lambert Law Absorption of [Ir] and [Ni], and the effect of concentration and pathlength on the reaction rate 51
6. Potential Mechanisms...54
6.1 Processes facilitating a general fit of the model 54
6.2 Processes for which a general fit of the model could not be found 55
7. In Situ UV-Vis Spectra and Extinction Coefficients...57
7.1 Photocatalyst 6...57
7.2 Ni(II) species 7 58
7.3 Changes in UV-Vis Spectrum during the Ar-Br/RBr cross coupling. 59
8. Behaviour of Iridium complex 6 on irradiation at 420 nm..60
9. Effect of endogenous and exogenous bromide ion on rate and selectivity. 61
10. References..64

1. General Considerations

All NMR spectra were recorded on a Bruker Avance III 400 MHz NMR spectrometer equipped with a Prodigy cryoprobe at 300 K. MestReNova versions 10.0 and 11.0 were used for processing all NMR spectra. All chemical shifts for ^{1}H NMR spectra are reported in parts per million (ppm) relative to the solvent residual signals. All chemical shifts for ^{19}F NMR are reported in ppm relative to an internal standard (fluorobenzene; δ = -113.14 ppm). All chemical shifts for ^{13}C NMR are reported in ppm relative to the solvent peak.

UV-Vis spectra were recorded in a quartz cuvette with 1cm path length on an Ocean-Optics Flame CCD Spectrometer connected via optical fibre to a DH2000-BALUV lamp. Acquisition was controlled by the Kinetic Studio software package (version 5). Data was processed and plotted using Excel.

Infrared (IR) spectra were recorded using a Bruker ALPHATM ATR-FTIR spectrometer. IR spectra were acquired over a range of 400-4000 cm^{-1} and peaks are reported in cm^{-1}.

A Bruker ESI Micro-Tof mass spectrometer was used to record all electrospray ionisation (ESI+) spectra. A Thermo Finnigan MAT900XP mass spectrometer was used to record electron impact (EI) spectra. Data acquired is reported as m/z.

Melting points (m.p.) were determined using a Griffin capillary apparatus and glass capillary tubes; the melting points are reported in °C.

Dimethoxyethane (Sigma-Aldrich) was purchased as an anhydrous liquid, then deoxygenated by sparging with nitrogen and stored in a N_2-filled MBraun glove box. Fluorobenzene, 2,6-lutidine, 4-bromobenzotrifluoride and 4-bromotetrahydropyran were distilled under vacuum and then stored in the same glove box. $(TMS)_3SiH$ (Sigma-Aldrich) was stored in the glove box without further purification. $(Ir[dF(CF_3)ppy]_2(dtbpy))PF_6$ (Strem Chemicals), $Ni(Cl)_2$.glyme (Sigma-Aldrich) and 4,4′-di-tert-butyl-2,2′-dipyridyl (dtbbpy) (Sigma Aldrich) were transferred immediately into the glovebox upon purchase. $Ni(COD)_2$ (Sigma Aldrich) was transferred immediately into the glovebox upon purchase and stored at –40 °C. All other reagents were obtained from commercial sources (Sigma Aldrich, Alfa Aesar, Fischer Scientific, Acros Organics and Fluorochem) and used without purification unless otherwise specified.

External irradiation of photochemical reactions was conducted at 365 nm, 420 nm or 450 nm using a Merck photoreactor. Internal irradiation of photochemical reactions was conducted using an LED-NMR apparatus constructed in-house and based on the design developed by Gshwind and co-workers.[S1] A range of fibre-coupled LEDs (λ = 365 nm, 420 nm and 455 nm) powered by a BLS-SA04-US LED driver was purchased from Mightex Systems. This was connected to an Opt 20L TTL trigger box from Hi-Tech Scientific, fitted with a BNC splitter to allow simultaneous communication with the NMR console and the LED driver. The operation of an external trigger switch by the experimenter thus causes the spectrometer to begin recording measurements simultaneously to the reaction beginning to be illuminated. The fibre-optic cable is a FP1500URT purchased from Thor Labs. In order to prepare the fibre for our desired purpose the various layers of cladding (external rubber, followed by Teflon fibres, then a loose plastic sheath, then several layers of adhered polymer) were carefully stripped away from one end to the appropriate length and the final layer of optical coating was removed by dissolution in acetone. The end of the cable was very gently roughened with fine sandpaper

to give uniform illumination in all directions, placed within a quartz coaxial insert (OD = 3.9 mm) and permanently attached using epoxy resin. All experiments are conducted within amberized standard 5 mm NMR tubes (ID = 3.97 mm) containing the fibre-optic cable held within the quartz insert to give a total reaction volume of 280 µL, with a light path length of 0.44 mm. Amberized NMR tubes and quartz coaxial inserts were both purchased from Norrell Scientific. The absolute photon flux delivered by this system into the solution at 420 nm was measured by the ferrioxolate actinometer to be 0.31 µE/s at maximum LED power (further details in section S4).

2. Synthesis

4-(4-(trifluoromethyl)phenyl)tetrahydro-2H-pyran,[S2] 3a

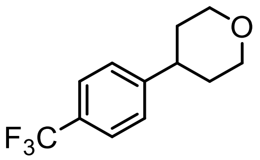

In order to confirm the in situ ^{19}F NMR assignment, **3a** was prepared according to an independent literature procedure.[S2] In a glovebox, (*S*)-(+)-prolinol (12 mg, 0.12 mmol, 12 mol%), $NiCl_2$·glyme (13.3 mg, 0.060 mmol, 6.0 mol%) and KHMDS (400 mg, 2.0 mmol, 2.0 equiv.) were placed into a Schlenk flask equipped with a stir bar. The Schlenk flask was removed from the glovebox and then 4-(trifluoromethyl)phenylboronic acid (165 mg, 1.5 mmol) was added. The flask was purged with nitrogen. Isopropanol (1.2 mL) was then added, and the resulting mixture was stirred for 5 minutes at room temperature. 4-Chlorotetrahydropyran (0.108 mL, 1.0 mmol) was then added by syringe, and the vial was heated at 60 °C. After 24 hours, the reaction mixture was allowed to cool to room temperature, and was then filtered through a short pad of silica gel (washing with 200 mL of 1:1 hexanes:ether). The filtrate was concentrated under vacuum, and the residue was purified by column chromatography. All spectral data agreed with the reported values.[S2] ^{1}H NMR (400 MHz, $CDCl_3$) δ 7.61 – 7.53 (m, 2H), 7.38 – 7.30 (m, 2H), 4.16 – 4.05 (m, 2H), 3.54 (td, *J* = 11.5, 2.7 Hz, 2H), 2.83 (tt, *J* = 11.4, 4.5 Hz, 1H), 1.97 – 1.70 (m, 4H). ^{19}F NMR (377 MHz, $CDCl_3$) δ -62.38. ^{19}F NMR (377 MHz, DME, relative to PhF) δ -61.57.

1-(4-trifluoromethylphenyl)-1,2-methoxyethane, 4

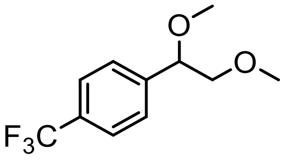

Following previously reported literature conditions,[S3] *p*-toluenesulfonic acid (0.172 g, 1 equiv.) was placed in a flask equipped with a stir bar. The flask was purged with N_2 and H_2O_2 (0.2 mL, 30% aqueous solution, 2 equiv.) was then added. The mixture was stirred for 5 min, then 4-(trifluoromethyl)styrene (0.15 mL, 1 mmol) was added. After 21 h stirring at 50 °C, the reaction mixture was neutralized with sodium bicarbonate, and reduced with Na_2SO_3, and then extracted with diethyl ether. After drying and removal of the volatiles *in vacuo*, 1-(4-trifluoromethylphenyl)-1,2-hydroxyethane diol was isolated as a white solid, with spectral data consistent with the reported values.[S4] The diol was carried forward to the next step without further purification. A flask containing NaH (0.028 g, 60% w/w, 3 eq.) and equipped with a magnetic stir bar was flushed with N_2 and cooled to 0 °C. 1-(4-Trifluoromethylphenyl)-1,2-hydroxyethane diol (0.0471 g, 0.23 mol) was dissolved in THF (3 mL) and added slowly to the NaH. The flask was allowed to warm to room temperature and stirred for 30 minutes, after which MeI (0.04 mL, 3 eq.) was added. After 22 hours the solution was washed with saturated ammonium chloride, extracted with ether and concentrated under reduced pressure.

Compound **4** was isolated as a colorless oil by column chromatography (7.9 mg, 14.7%). ^{1}H NMR (400 MHz, $CDCl_3$) δ 7.65 (d, *J* = 7.8 Hz, 2H), 7.48 (d, *J* = 8.0 Hz, 2H), 4.46 (dd, *J* = 7.6, 3.8 Hz, 1H), 3.60 (dd, *J* = 10.4, 7.6 Hz, 1H), 3.46 (dd, *J* = 10.4, 3.8 Hz, 1H), 3.41 (s, 3H), 3.33 (s, 3H). ^{13}C{^{1}H} NMR (101 MHz, $CDCl_3$) δ 127.29, 125.45 (q), 82.47, 77.21, 59.35, 57.29. Note the CF_3 and both quaternary Cs were not detected. ^{19}F NMR (377 MHz, $CDCl_3$) δ -62.56. ^{19}F NMR (377 MHz, DME, relative to PhF) δ -61.72. HRMS (ESI) calculated for $C_{11}H_{13}F_3O_2Na_1$ 257.07599, observed 257.0758. ATR FT-IR v_{max} /cm^{-1} 2987, 2932, 2893, 2828, 1322, 1163, 1102, 1065, 1017, 837, 609.

4,4′-Bis(trifluoromethyl)biphenyl S1

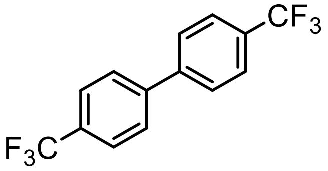

Biaryl **S1** was prepared using a previously reported procedure.[S5] All spectral data agreed with previously reported values.[S6] ^{1}H NMR (400 MHz, $CDCl_3$) δ 7.75-7.69 (m, 8H). ^{19}F NMR (377 MHz, $CDCl_3$) δ-62.58. ^{19}F NMR (377 MHz, DME, relative to PhF) δ-62.32. ^{13}C{^{1}H} NMR (101 MHz, $CDCl_3$) δ 143.42 (s), 130.47 (q, $^2J_{CF}$ = 32.54 Hz), 127.79 (s), 126.11 (q, $^3J_{CF}$ = 3.96 Hz), 124.26 (q, $^1J_{CF}$ = 272 Hz). HRMS (EI) calculated for $C_{14}H_8F_6$ 290.05247, observed 290.052516

N-(4-fluorobenzoyl)-4-bromopiperidine,[S7] 1b

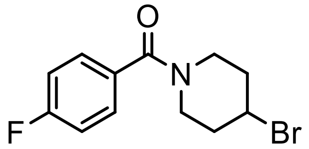

4-Fluorobenzoyl chloride (12 mL, 0.1 mol, 1 equiv.) was slowly added to a mixture of 4-hydroxypiperidine (10 g, 0.1 mol, 1 equiv.), and Et_3N (29 mL, 200 mmol, 2 equiv) in DCM (150.0 mL) at -10 ℃. The reaction mixture was warmed to room temperature. After it was stirred for 2h, the reaction mixture was washed with HCl (1M) and then brine. The organic layer was dried over $MgSO_4$, filtered and concentrated *in vacuo*, and the resulting solid recrystalised from EtOAc to afford *N*-(4-fluorobenzoyl)-4-hydroxypiperidine (15.7 g, 70%) as white solid that was used without further purification. m.p. 106-109 °C. ATR FT-IR v_{max} /cm^{-1} 3307, 3071, 2924, 2876, 1600, 1585, 1454, 1286, 1221, 1030, 1002, 966, 848, 587. ^{1}H NMR (400 MHz, $CDCl_3$) δ 7.48 – 7.39 (m, 2H), 7.17 – 7.06 (m, 2H), 4.15 (s, broad, 1H) 4.01 (tt, *J* = 8.0, 3.8 Hz, 1H), 3.77 (s, broad, 1H), 3.32 (s, broad, 2H), 1.93 (s, broad, 2H), 1.58 (s, broad, 2H). ^{13}C NMR (101 MHz, $CDCl_3$) δ 169.54, 164.62, 132.08, 129.20, 129.11, 115.69, 115.47, 77.34, 77.02, 76.70, 67.15. ^{19}F (377 MHz, $CDCl_3$) δ -110.46 (1F, m). HRMS (ESI) calculated for MH^+ $C_{12}H_{15}FNO_2$ 224.10813, observed 224.10850. A solution of *N*-(4-fluorobenzoyl)-4-hydroxypiperidine (15.66 g, 1 equiv.) in pyridine (55 mL) was stirred at 0 °C. TsCl (1.5 equiv, 20.07 g) was added portion-wise and the reaction was stirred at 0 °C for five hours. DCM and

water were added and the organic layer was washed successively with 2M HCl, saturated aqueous $NaHCO_3$, and then water, and then dried ($MgSO_4$). The DCM was removed *in vacuo* and the residue triturated with diethyl ether to afford *N*-(4-fluorobenzoyl)-4-tolunesulfonylpiperidine as white solid (12.44 g, 47 %) that was used without further purification. ^{1}H NMR (400 MHz, $CDCl_3$) δ 7.86 – 7.78 (m, 2H), 7.46 – 7.34 (m, 4H), 7.16 – 7.05 (m, 2H), 4.81 (tt, J = 6.7, 3.8 Hz, 1H), 3.70 (s, broad, 4H), 2.48 (s, 3H), 1.82 (s, broad, 4H). ^{13}C NMR (101 MHz, $CDCl_3$) δ 169.71, 145.11, 134.32, 130.12, 129.39, 129.30, 127.78, 115.93, 115.71, 21.82. ^{19}F NMR (377 MHz, $CDCl_3$) δ -109.93 (1F, m). Lithium bromide (7.5 equivs, 20 g) was heated in acetone (100 mL) in a flask equipped with a reflux condenser. Once all the LiBr had dissolved, *N*-(4-fluorobenzoyl)-4-tolunesulfonylpiperidine (12.5 g) was added and the mixture held at reflux until TLC showed complete consumption of the tosylate (approximately 3 hours). The solvent was removed under reduced pressure, and the residue was dissolved in a minimum amount of water and extracted with EtOAc (3 × 50 mL). The combined organics were washed with 0.1 M KOH and brine, dried with $MgSO_4$, and concentrated in vacuo. The residue was purified by column chromatography (1:1 EtOAc:40-60 petroleum ether) and *N*-(4-fluorobenzoyl)-4-tolunesulfonylpiperidine **1b** (2.8 g, 29.8%) isolated after recrystallisation from cyclohexane. The NMR data is in good agreement with that reported.[S7] m.p. (77-78°C), ATR FT-IR v_{max}/cm^{-1} 3068, 2927, 1621, 1596, 1437, 1274, 1218, 1132, 999, 844, 760, 589. ^{1}H NMR (400 MHz, $CDCl_3$) δ 7.50 – 7.39 (m, 2H), 7.17 – 7.07 (m, 2H), 4.46 (tt, J = 7.3, 3.8 Hz, 1H), 3.64 (d, broad, J = 113.1 Hz, 4H), 2.03 (d, broad, 4H). ^{13}C NMR (101 MHz, $CDCl_3$) δ 169.72, 164.84, 162.36, 131.83, 131.80, 129.40, 129.32, 115.90, 115.69, 48.77, 27.05. ^{19}F (377 MHz, $CDCl_3$) δ -110.07 (1F, m). ^{19}F (377 MHz, DME, relative to PhF) δ -111.32 (1F, m). ESI MS MH$^-$ 286.06.

N-4-fluorobenzoylpiperidine,[S8] S2

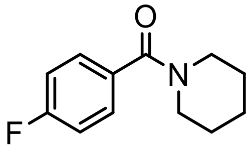

N-4-fluorobenzoylpiperidine **S4** was prepared from 4-fluorobenzoyl chloride (0.12 mL, 1 mmol, 1 equiv.) and piperidine (0.1 mL, 1 mmol, 1 equiv.) following the same general procedure employed for *N*-(4-fluorobenzoyl)-4-hydroxypiperidine. The NMR data is in good agreement with that reported.[S8] ^{1}H NMR (400 MHz, $CDCl_3$) δ 7.51 – 7.35 (m, 2H), 7.19 – 7.04 (m, 2H), 3.54 (d, broad, J = 120.1 Hz, 4H), 1.88 – 1.40 (m, broad, 6H). ^{19}F (377 MHz, $CDCl_3$) δ -110.97 (1F, m). ^{19}F (377 MHz, DME, relative to PhF) δ -111.9 (1F, m).

(4,4′-di-tert-butyl-2,2′-bipyridine)Ni(4-trifluoromethylphenyl)(Br),[S9] 7

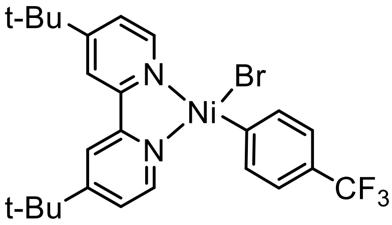

Complex **7** was prepared according to the a reported procedure.[SC] In a nitrogen filled glove box, $Ni(COD)_2$ (276 mg, 1 mmol, 1.0 equiv) and 4,4'-di-tert-butyl-2,2'-bipyridine (268 mg, 1 mmol, 1.0 equiv) were dissolved in dry tetrahydrofuran (5 mL) to give a dark purple solution. This was stirred for 12 hours at room temperature. 4-Bromobenzotrifluoride (2.8 mL, 20 mmol, 20.0 equiv) was then added. The solution was stirred for approximately 30 minutes until a deep red colour was observed. Ten minutes after observation of the colour change, dry pentane (30 mL) was added to the solution and the resulting precipitate immediately collected by filtration. The brick-red solid was then washed with pentane (3 x 10 mL) and dried under vacuum to afford complex **7** (398 mg, 72%). The complex was stored in a nitrogen filled glove box at -40 ^{o}C and used without further purification. The NMR data was consistent with that previously reported.[S9] ^{1}H NMR (400 MHz, CD_2Cl_2) δ 9.26 (d, J = 5.9 Hz, 1H), 7.89 (d, J = 7.0 Hz, 2H), 7.80 (d, J = 7.8 Hz, 2H), 7.54 (d, J = 5.5 Hz, 1H), 7.32 – 7.03 (m, 4H), 1.45 (br s, 9H), 1.35 (br s, 9H). ^{19}F NMR (377 MHz, CD_2Cl_2) δ -61.93 (s, 3F). ^{19}F NMR (377 MHz, DME, referenced to PhF) δ -60.7 (s, 3F). HRMS (EI) calculated for $C_{25}H_{28}{}^{79}BrF_3N_2{}^{58}Ni$ 550.07596, observed 550.07359.

[$^{13}CF_3$]-4-bromobenzotrifluoride, [$^{13}CF_3$]-2

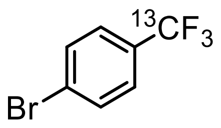

1,4-Dibromobenzene (2.642 g, 11.2 mmol) was dissolved in tetrahydrofuran (30 mL) and cooled at -78° C under nitrogen. A solution of n-butyl lithium in hexanes (4.5 mL, 11.2 mmol, 2.5 M) was added slowly and the reaction was stirred at -78 °C for 1 hour. Meanwhile, concentrated H_2SO_4 was added dropwise to a solution of $Na_2{}^{13}CO_3$ (1.99 g) in water (6 mL) until evolution of gas ceased. The released $^{13}CO_2$ was captured and condensed at -78° C under nitrogen. The pellet of $^{13}CO_2$ (0.69 g) was rapidly added to the solution of 1-lithio-4-bromobenzene and the mixture allowed to slowly warm to ambient temperature while stirring for 1 h. Water was slowly added at 0° C and the mixture was washed with diethyl ether (×3). HCl (conc.) was added to until the solution was acidic (pH paper) resulting in generation of a white precipitate. The mixture was then extracted with diethyl ether (×3) and dried with $MgSO_4$, and the solvent was removed *in vacuo* to yield 1-[$^{13}CF_3$]-4-bromobenzoic acid as a white solid (1.82 g, 72%) that was used without further purification. The NMR data is consistent with that expected, based on reference to commercially-available natural abundance 4-bromobenzoic acid. ^{1}H NMR (400 MHz, $CDCl_3$) δ 7.93 (m, 2H), 7.61 (d, J = 7.78 Hz). ^{13}C (101 MHz, $CDCl_3$) δ 169.4 (4-bromobenzoic acid has very limited solubility in $CDCl_3$; only the signal for the

[$^{13}CF_3$]-group is reported). Following a previously reported procedure for preparation of natural abundance 4-bromobenzotrifluoride from 1-CF_3-4-bromobenzoic acid,[S10] an oven-dried wide-mouth screw-top FEP bottle (Nalgene®) (60.0 mL) containing a magnetic stir bar was charged with 1-[$^{13}CF_3$]-4-bromobenzoic acid (1.5 mmol, 0.302 g) and Fluolead (1.127 g, 4.5 mmol, 3.0 equiv.). 70% HF·Pyridine (7.5 mmol, 5 equiv., 0.26 mL, neat) was added with a plastic syringe (**CAUTION**; this was conducted in a well-ventilated fume-hood, with the operator wearing full and appropriate PPE. The empty syringe was immediately quenched in aqueous $NaHCO_3$). The screw-top FEP bottle was tightly sealed and then heated, with stirring, in an oil bath at 70 °C for 24 hours. (**CAUTION** this was conducted in a well-ventilated fume-hood, with a protective screen between the oil bath and the operator, with the operator wearing full and appropriate PPE during manipulations. Note that the head space of the screw-top FEP bottle was chosen to be considerably larger than the reaction volume). After cooling the bottle to room temperature and cautiously opening it, wet isopropanol (15 mL) was added; the resultant mixture was stirred at room temperature for a further 30 minutes. (**CAUTION** this was conducted in a well-ventilated fume-hood, with a protective screen in place between the bottle and the operator, and with the operator wearing full and appropriate PPE). The solution was then added to saturated aqueous $NaHCO_3$ (20 mL) at 0°C which was then extracted with pentane (3 x 30mL). The combined organic layer was dried over anhydrous $MgSO_4$, filtered and concentrated using gentle heating. Careful kugelrohr distillation afforded [$^{13}CF_3$]-4-bromobenzotrifluoride, [$^{13}CF_3$]-**2** as a colourless liquid (b.p. 155 °C; 39 mg, 11.5%. The NMR data is consistent with that expected, based on reference to a commercial sample of natural abundance 4-bromobenzotrifluoride. ^{1}H NMR (400 MHz, $CDCl_3$) δ 7.63 (d, J = 7.8 Hz, 2H), 7.54 – 7.44 (dd, J = 8.69 Hz, 4.18 Hz, 2H). ^{19}F NMR (377 MHz, $CDCl_3$) δ -62.9 (d, J = 272 Hz, 3F). ^{13}C (101 MHz, $CDCl_3$) δ 123.9 (q, J = 272 Hz, 1C). HRMS (EI) calculated for $C_6{}^{13}C_1H_4{}^{79}Br_1F_3$ 224.94807, observed 224.94765.

3. Kinetic Studies

3.1 General Procedure for *in situ* Kinetic Analyses

In a N_2-filled glovebox, a solution of fluorobenzene (internal standard, IS) in 1,2-dimethoxyethane (DME, 0.03 M) was prepared and this then used to make up all other solutions to ensure [IS] was constant in all reactions. For each reactant, reagent, or catalyst, the appropriate amount was added to various volumetric flasks, vide infra, within the glovebox (solid weighed on a microbalance, liquids volumetrically by microsyringe) and the flask filled to the line using the IS stock solution. For the Ni-catalysts stock solution, $NiCl_2$.glyme and dtbbpy were weighed into the same volumetric flask and the solution was stirred for at least ten minutes (a colour change was observed, along with dissolution of all solids) prior to addition to a reaction. Typical stock solution concentrations for each species were: 0.1 M ArBr, 0.15 M AlkBr, 0.1M $(TMS)_3SiH$, 0.2 M 2,6-lutidine, 0.0025 M $NiCl_2$(dtbbpy), 0.005 M $Ir(dF(CF_3)ppy)_2(dtbbpy)(PF_6)$. Stock solutions for all stoichiometric reagents were stored in a glovebox freezer at -40 °C; stock solutions of Ni and Ir catalysts were prepared freshly on the day of reaction. The reaction mixture was assembled in the glovebox by addition of the appropriate volume of each stock solution to a volumetric flask; the remaining volume was made up with the IS stock solution. Each reaction mixture was used within 24 hours of being made; in the case it was not used immediately, it was stored in the dark in a glovebox freezer at -40 °C. The assembled reaction was mixed well and measured (280 µL) into an amberized NMR tube in the glovebox. This NMR tube was then removed from the glovebox; the fibre optic containing quartz coaxial insert was inserted and the assembled NMR tube was placed inside the NMR spectrometer. The spectrometer was shimmed on the ^1H frequency, and the resultant NMR spectrum was checked to ensure proper lineshape had been achieved. The spectrometer was then tuned to ^{19}F and a reference spectrum was taken. The sample was then irradiated whilst subsequent NMR spectra were taken until analysis was complete.

The spectra were stacked and bulk-processed; both a phase and a baseline correction were typically applied. Each peak was integrated and concentrations of all substrates calculated in excel by comparison of substrate integral to that of the fluorobenzene internal standard.

3.2 Representative temporal-concentration data

3.2.1 4-bromotetrahydropyran, 1a, as AlkBr

a)

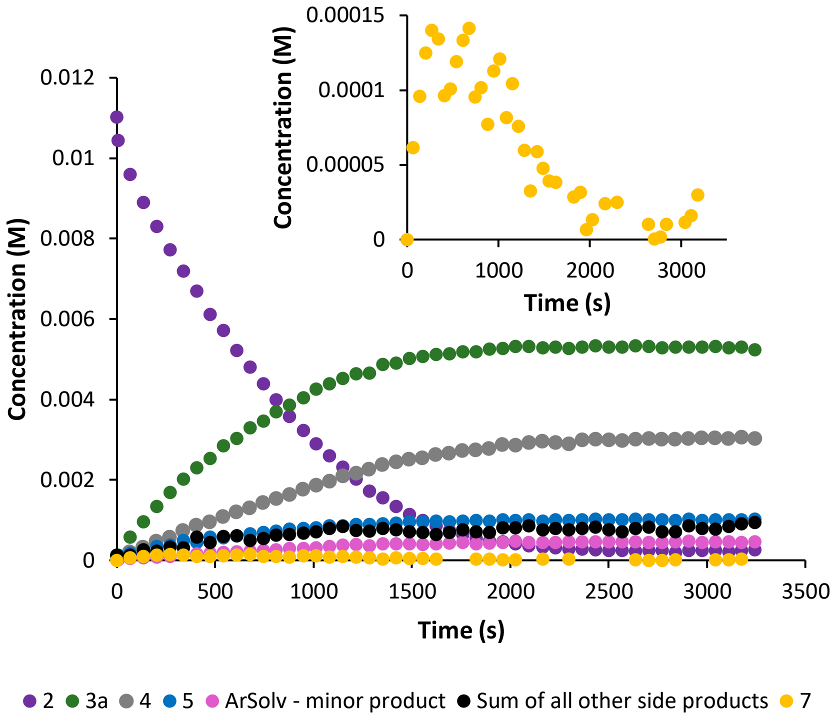

b)

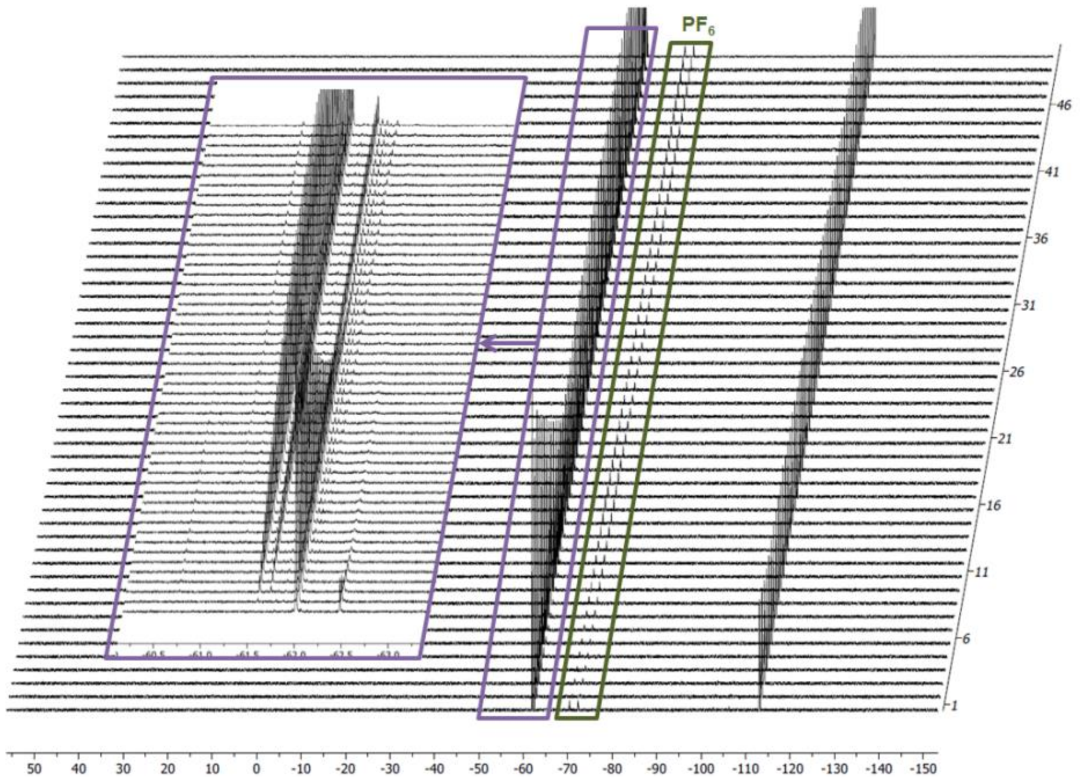

cont.

c)

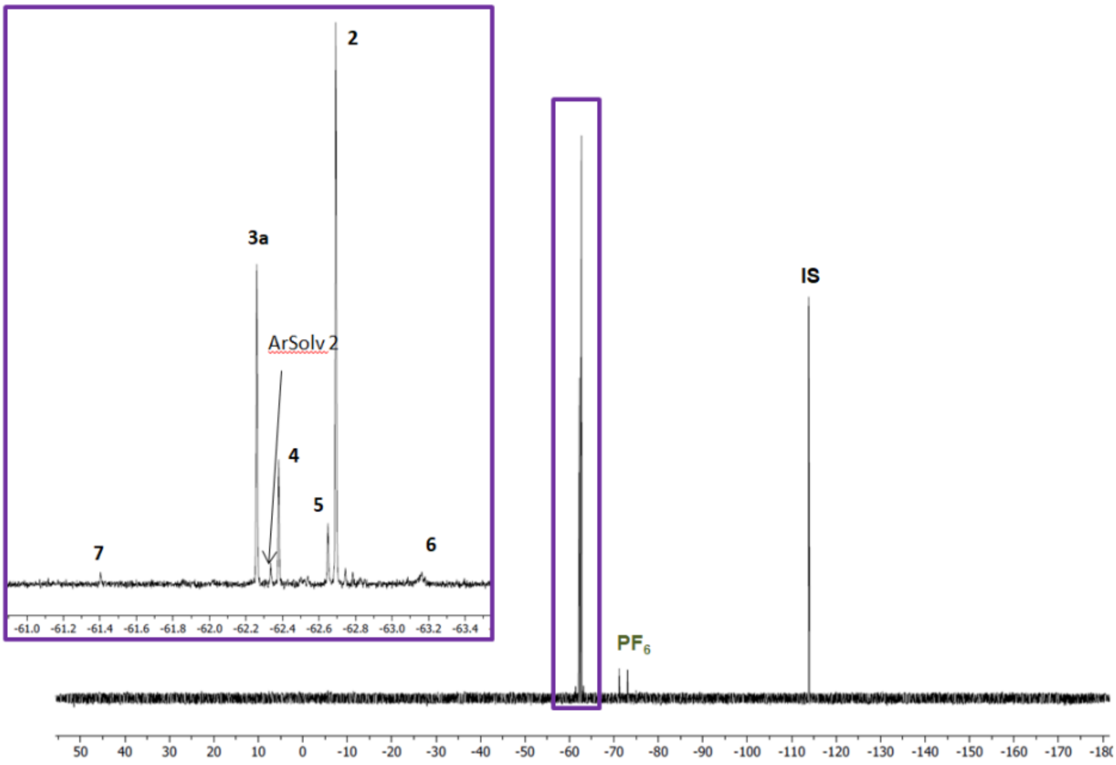

Figure S1. (continued from previous page) a) Kinetic trace of a 'typical' reaction using **1a** as the alkyl bromide substrate. Initial conditions: $[\mathbf{1a}]_0$ = 15 mM; $[\mathbf{2}]_0$ = 11 mM; $[Ir]_{tot}$ = 0.5 mM; $[Ni]_{tot}$ = 0.25 mM; $[(TMS)_3SiH]_0$ = 10 mM; $[2,6\text{-lutidine}]_0$ = 20 mM; I_0 = 1.1 $mEL^{-1}s^{-1}$ (420 nm). The intermediate **7** signal is shown as an inset graph. b) The stacked NMR spectra of the same kinetic trace. c) Single spectrum of a partially completed reaction, showing the locations of the major peaks present during the reaction; IS = internal standard.

3.2.2 N-(4-fluorobenzoyl)-4-bromopiperidine, 1b, as AlkBr

a)

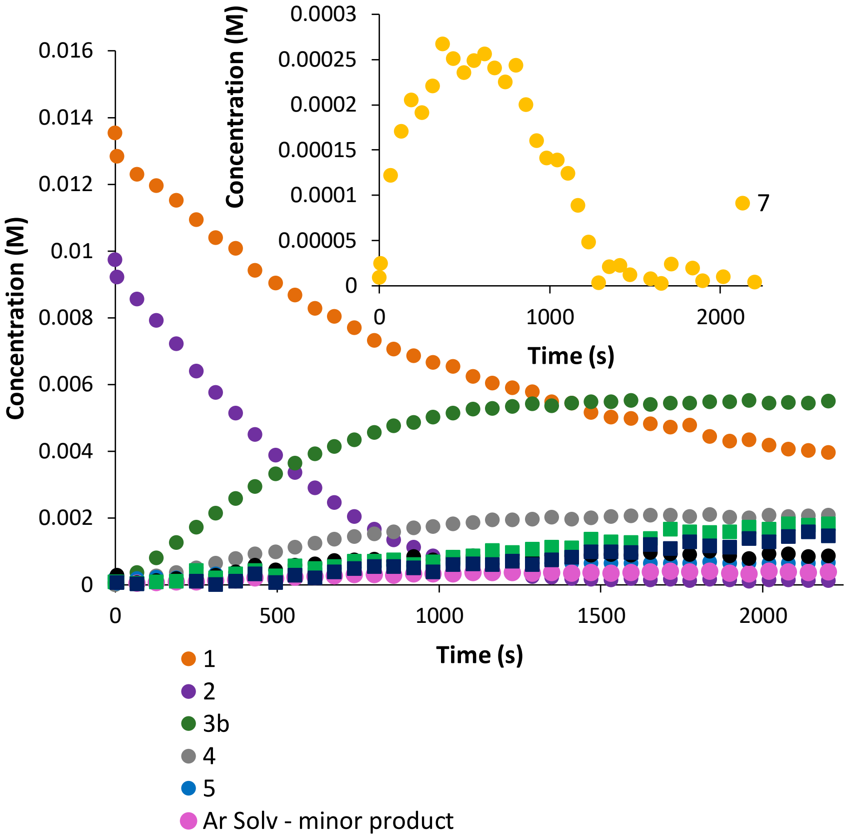

b)

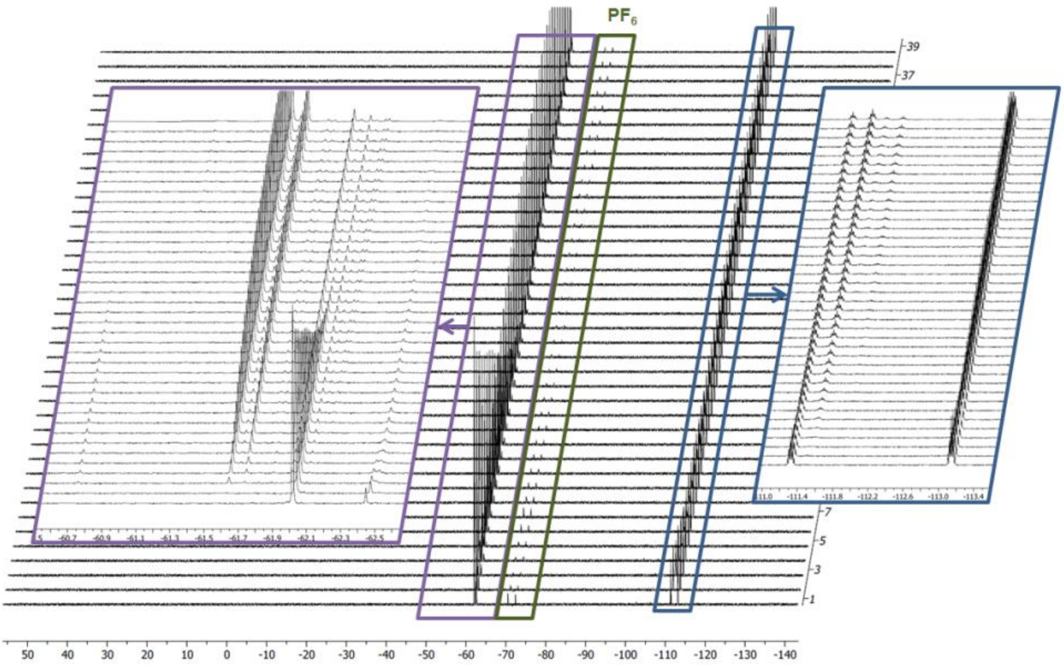

cont.

c)

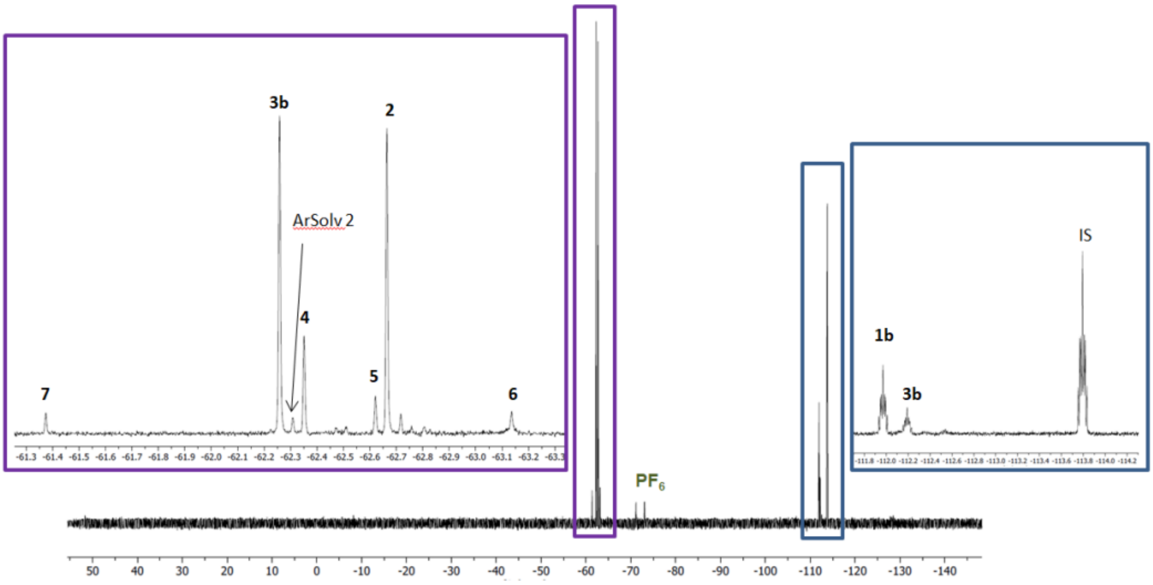

Figure S2. (continued from previous page) a) Kinetic trace of a 'typical' reaction using 1b as the alkyl bromide substrate. Initial conditions: [**1b**]$_0$ = 14 mM; [**2**]$_0$ = 10 mM; [Ir]$_{tot}$ = 0.5 mM; [Ni]$_{tot}$ = 2.5 mM; [(TMS)$_3$SiH]$_0$ = 10 mM; [2,6-lutidine]$_0$ = 20 mM; I$_0$ = 1.1 mEL^{-1}s^{-1} (420 nm). The intermediate **7** signal is shown as an inserted graph. b) The stacked NMR spectra of the same kinetic trace. c) Single spectrum of a partially completed reaction, showing the locations of the major peaks present during the reaction; IS = internal standard.

3.3 Control Experiments

3.3.1 Comparison of reactions at 455 nm and 420 nm

(**a**)

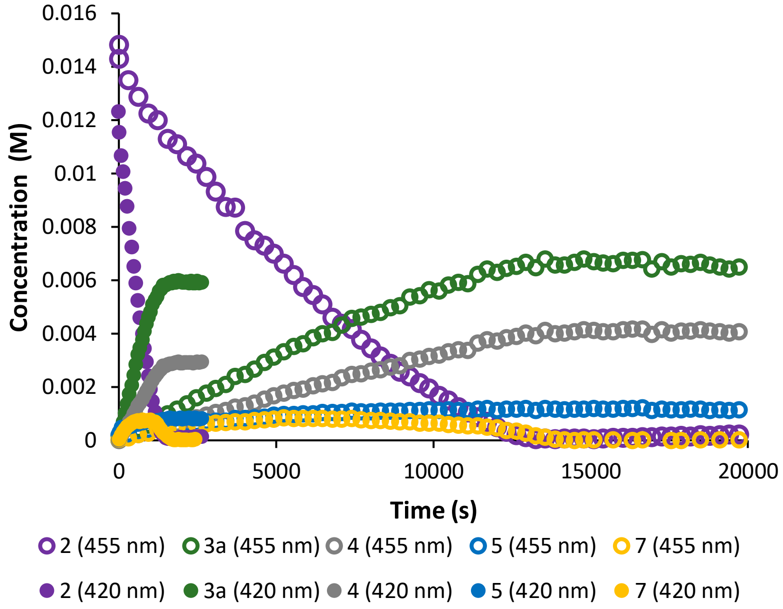

(**b**)

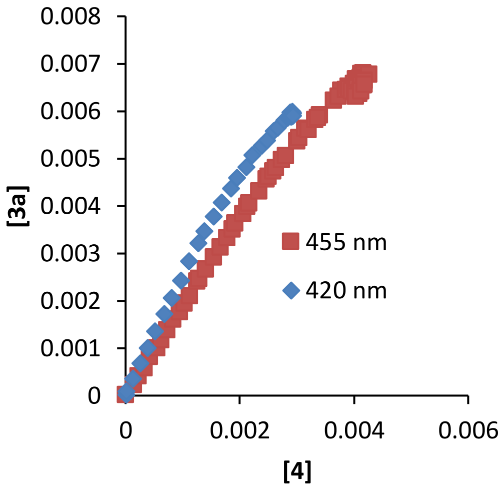

Figure S3. (a) Overlayed kinetic profiles of reaction from Figures S2 with the same process conducted at 455 nm ([**1a**]$_0$ = 20 mM; [**2**]$_0$ = 15 mM; [Ir]$_{tot}$ = 0.75 mM; [Ni]$_{tot}$ = 0.38 mM; [(TMS)$_3$SiH]$_0$ = 15 mM; [2,6-lutidine]$_0$ = 30 mM; I_0 = 1.1mEL^{-1}s^{-1}, 455 nm). The kinetic behaviour is similar in terms of selectivity, but the solution irradiated at 455 nm (0.17 µE/s) reacts significantly slower than that irradiated at 420 nm (0.31 µE/s). (**b**) Overlayed concentration-concentration plots (data from Figures S2 and S3) showing the relative partitioning of **3a** and **4** when the reaction is conducted at 420 nm and 455 nm respectively. The plots highlight that similar kinetic behaviour is observed at the two wavelengths.

3.3.2 No Photocatalyst

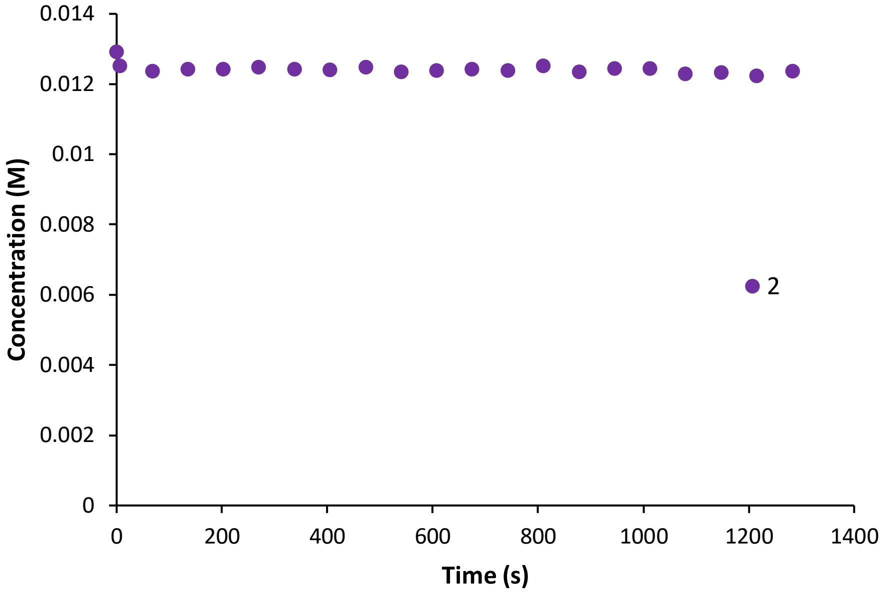

Figure S4. Kinetic profile of a reaction conducted with no photocatalyst present, demonstrating a lack of reaction. Initial conditions: [**1a**]$_0$ = 15 mM; [**2**]$_0$ = 13 mM; [Ni]$_{tot}$ = 0.25 mM; [(TMS)$_3$SiH]$_0$ = 10 mM; [2,6-lutidine]$_0$ = 20 mM; I_0 = 1.1 mEL^{-1}s^{-1} (420 nm).

3.3.3 No AlkBr

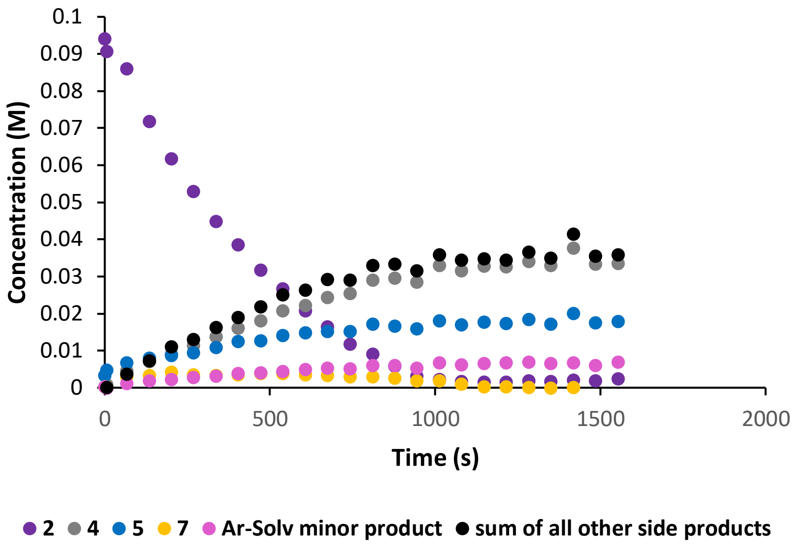

Figure S5. Kinetic profile of a reaction conducted with all components except the alkyl bromide (**1a** or **1b**) present, demonstrating consumption of aryl bromide starting material **2**, the formation of ArNi intermediate **7** and formation of other Ar-containing side products. Initial conditions: [**2**]$_0$ = 9.5 mM; [Ir]$_{tot}$ = 0.5 mM; [Ni]$_{tot}$ = 0.25 mM; [(TMS)$_3$SiH]$_0$ = 10 mM; [2,6-lutidine]$_0$ = 20 mM; I$_0$ = 1.1 mEL^{-1}s^{-1} (420 nm).

3.3.4 No Silane

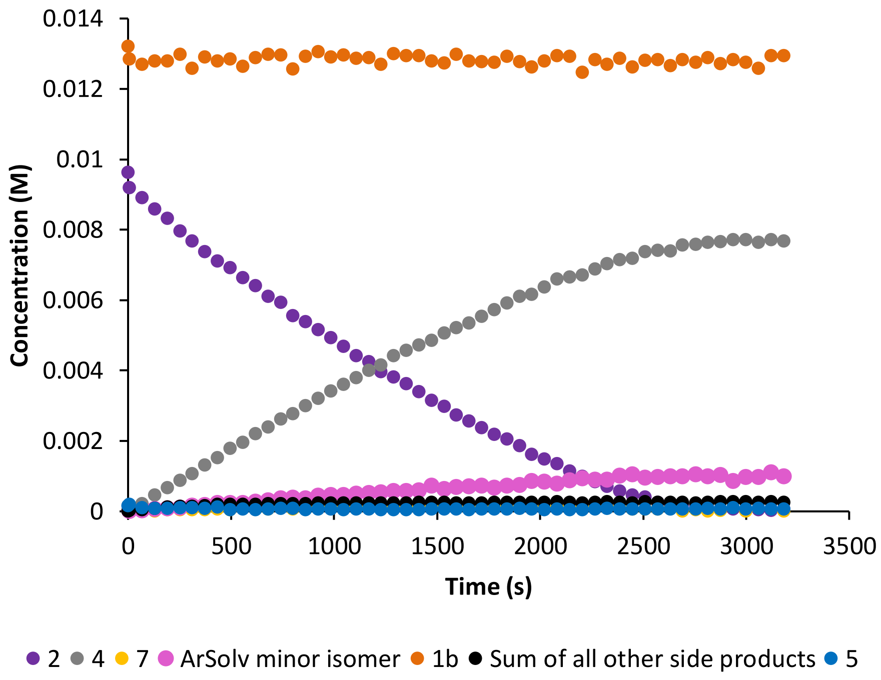

Figure S6. Kinetic profile of a reaction conducted with all components except the silane, demonstrating consumption of aryl bromide starting material **2** and formation of Ar-Solv byproducts near-exclusively. Note that trace amounts of protodebrominated side product **5** and ArNi intermediate **7** were also observed. Initial conditions: [**1b**]$_0$ = 13 mM; [**2**]$_0$ = 10 mM; [Ir]$_{tot}$ = 0.5 mM; [Ni]$_{tot}$ = 0.25 mM; [2,6-lutidine]$_0$ = 20 mM; I$_0$ = 1.1 mEL^{-1}s^{-1} (420 nm).

3.3.5 No Base

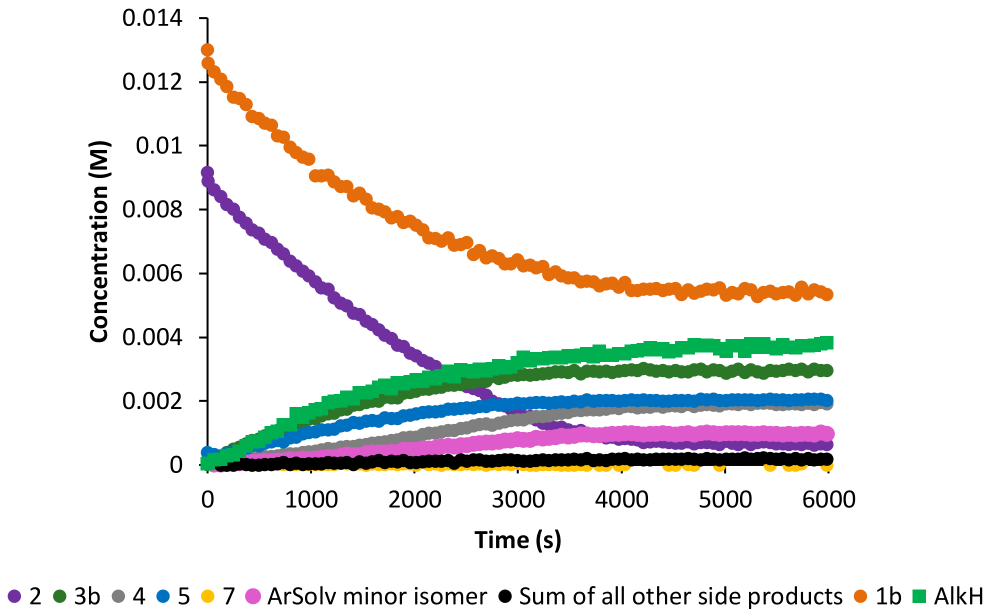

Figure S7. Kinetic profile of a reaction conducted with all components but the base present. The reaction stalls befor full conversion of **2**. Initial conditions: [**1b**]$_0$ = 13 mM; [**2**]$_0$ = 9 mM; [Ir]$_{tot}$ = 0.5 mM; [Ni]$_{tot}$ = 0.25 mM; [[(TMS)$_3$SiH]$_0$ = 10 mM; I$_0$ = 1.1 mEL^{-1}s^{-1} (420 nm).

3.3.6 Midpoint ^{19}F NMR control reactions

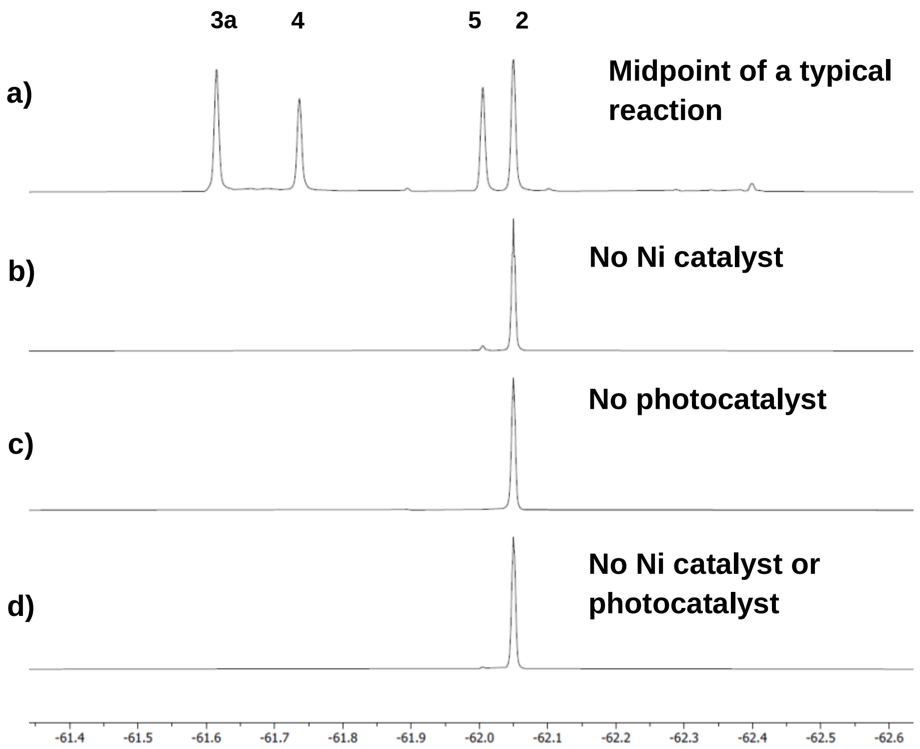

Figure S8. a) major ^{19}F NMR signals corresponding to the starting material (**2**) and products (**3a, 4, 5**). Initial conditions: [**1a**]$_0$ = 15 mM; [**2**]$_0$ = 10 mM; [Ir]$_{tot}$ = 0.5 mM; [Ni]$_{tot}$ = 0.25 mM; [(TMS)$_3$SiH]$_0$ = 10 mM; [2,6-lutidine]$_0$ = 20 mM; I$_0$ = 1.1 mEL^{-1}s^{-1}(420 nm, 20 h exposure). b), c) and d) Identical reaction solutions containing all components except for Ni catalyst (b), photocatalyst (c), Ni catalyst and photocatalyst (d).

3.3.9 Light "on-off" studies

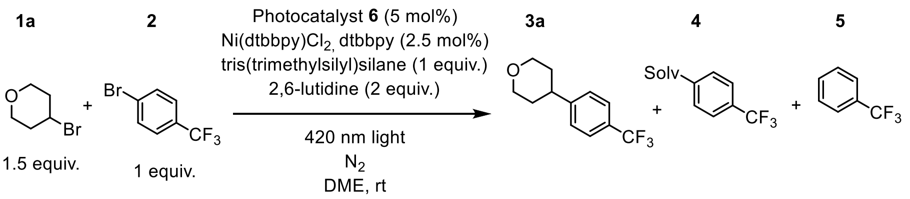

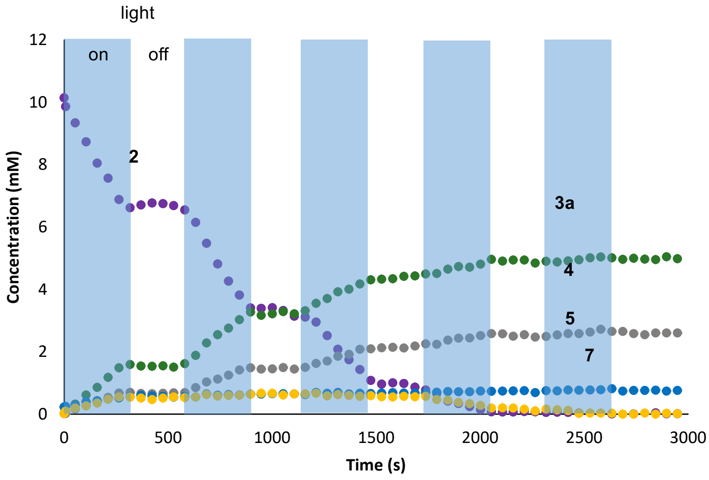

Figure S9. Kinetic profile demonstrating the effects of alternating between lights on and lights off regimes on the behaviour of the system. Initial conditions: [**1a**]$_0$ = 15 mM; [**2**]$_0$ = 10 mM; $[Ir]_{tot}$ = 0.5 mM; $[Ni]_{tot}$ = 0.25 mM; $[(TMS)_3SiH]_0$ = 10 mM; [2,6-lutidine]$_0$ = 20 mM; I_0 = 1.1 mEL^{-1}s^{-1} (420 nm).

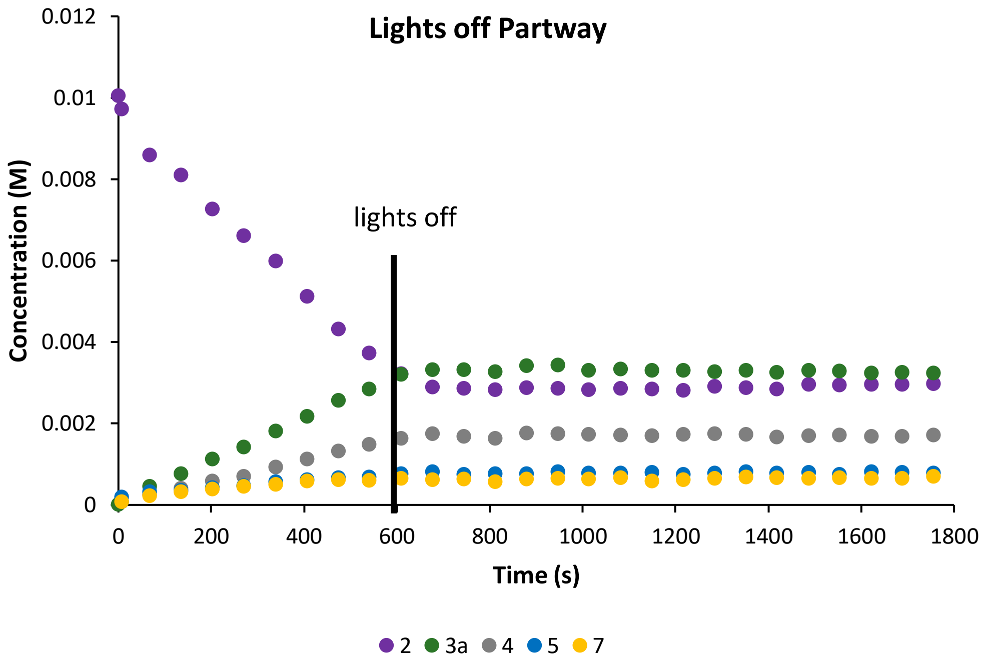

Figure S10. Kinetic profile demonstrating the effects of leaving a partially-reacted system in the dark for a more extended period of time, verifying the persistence of intermediate **7** in the absence of light. Initial conditions: [**1a**]$_0$ = 15 mM; [**2**]$_0$ = 10 mM; $[Ir]_{tot}$ = 0.5 mM; $[Ni]_{tot}$ = 0.25 mM; $[(TMS)_3SiH]_0$ = 10 mM; [2,6-lutidine]$_0$ = 20 mM; I_0 = 1.1 mEL^{-1}s^{-1} (420 nm).

3.3.10 ^{1}H and ^{29}Si NMR spectroscopic analysis of silane.

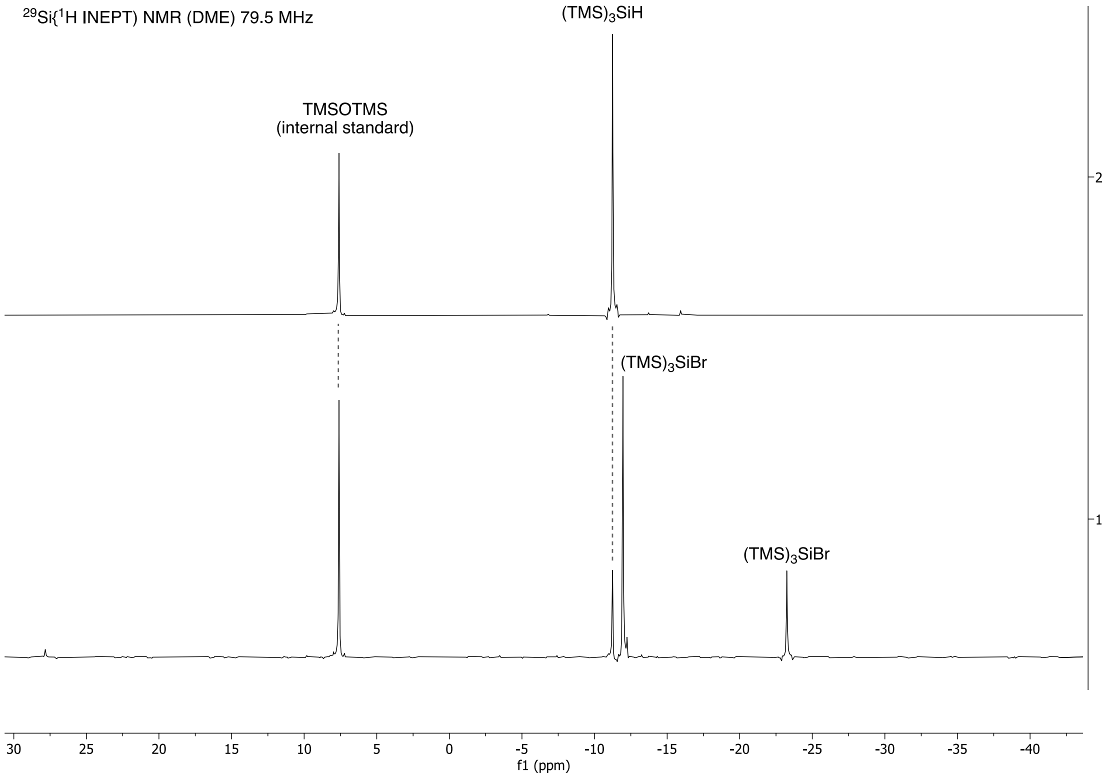

Figure S11. ^{29}Si NMR spectroscopic analysis of the cross-coupling reaction conducted in a J Young-valve sealed NMR tube, using an external light source (420 nm) to irradiate the reaction, and run at higher concentration than the standard process to improve signal/noise in the NMR analysis. Initial conditions: [**1a**]$_0$ = 150 mM; [**2**]$_0$ = 100 mM; [Ir]$_{tot}$ = 5 mM; [Ni]$_{tot}$ = 2.5 mM; [(TMS)$_3$SiH]$_0$ = 100 mM; [2,6-lutidine]$_0$ = 200 mM; 420 nm, plus TMSOTMS spike used as a chemical shift reference (δ = 7.6 ppm). $^{29}Si(^{1}H$ INEPT) NMR spectrum 2 (upper) is taken at the start of reaction, spectrum 1 (lower) at end of reaction. The recorded and reported ^{29}Si NMR shifts (in C_6D_6) are (TMS)$_3$SiH (δ = –11.9 ppm) and (TMS)$_3$SiBr (δ = –23.5 ppm, –12.3 ppm).[S11]

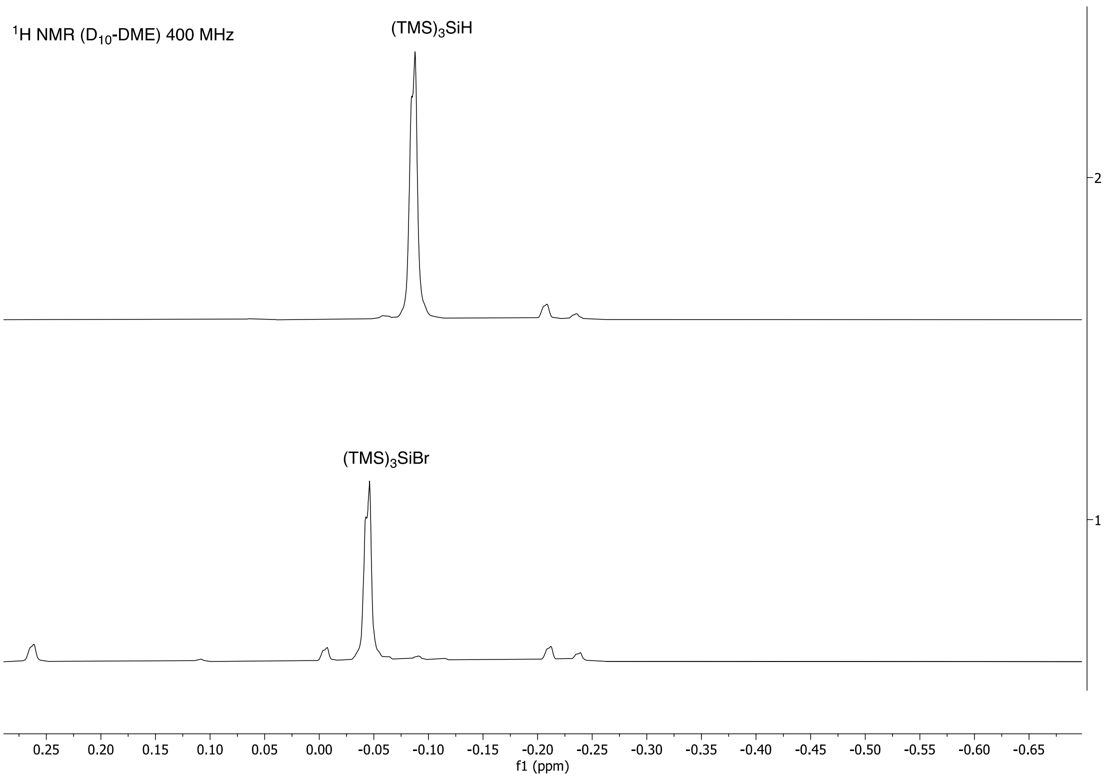

Figure S12. ^{1}H NMR spectroscopic analysis of the cross-coupling reaction conducted in the LED NMR apparatus in D_{10} DME. Initial conditions: [**1a**]$_0$ = 15 mM; [**2**]$_0$ = 10 mM; [Ir]$_{tot}$ = 0.5 mM; [Ni]$_{tot}$ = 0.25 mM; [(TMS)$_3$SiH]$_0$ = 10 mM; [2,6-lutidine]$_0$ = 20 mM; 420 nm. ^{1}H NMR spectrum 2 (upper) is taken at the start of reaction, and spectrum 1 (lower) at end of reaction. The reported ^{1}H NMR chemical shifts (in $CDCl_3$) are (TMS)$_3$SiH (δ = 0.20 ppm) and (TMS)$_3$SiBr (δ = 0.25 ppm); i.e. $\Delta\delta$ = + 0.05.[S12]

3.4 Reactivity of NiArBr complex 7

3.4.1 Stoichiometric reaction of 7 with Alkyl Bromide 1b

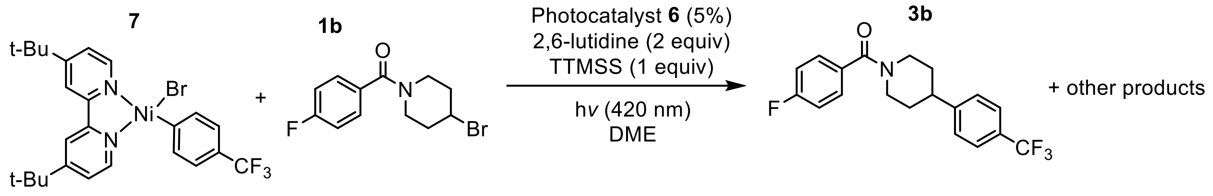

1 equiv 1.5 equiv

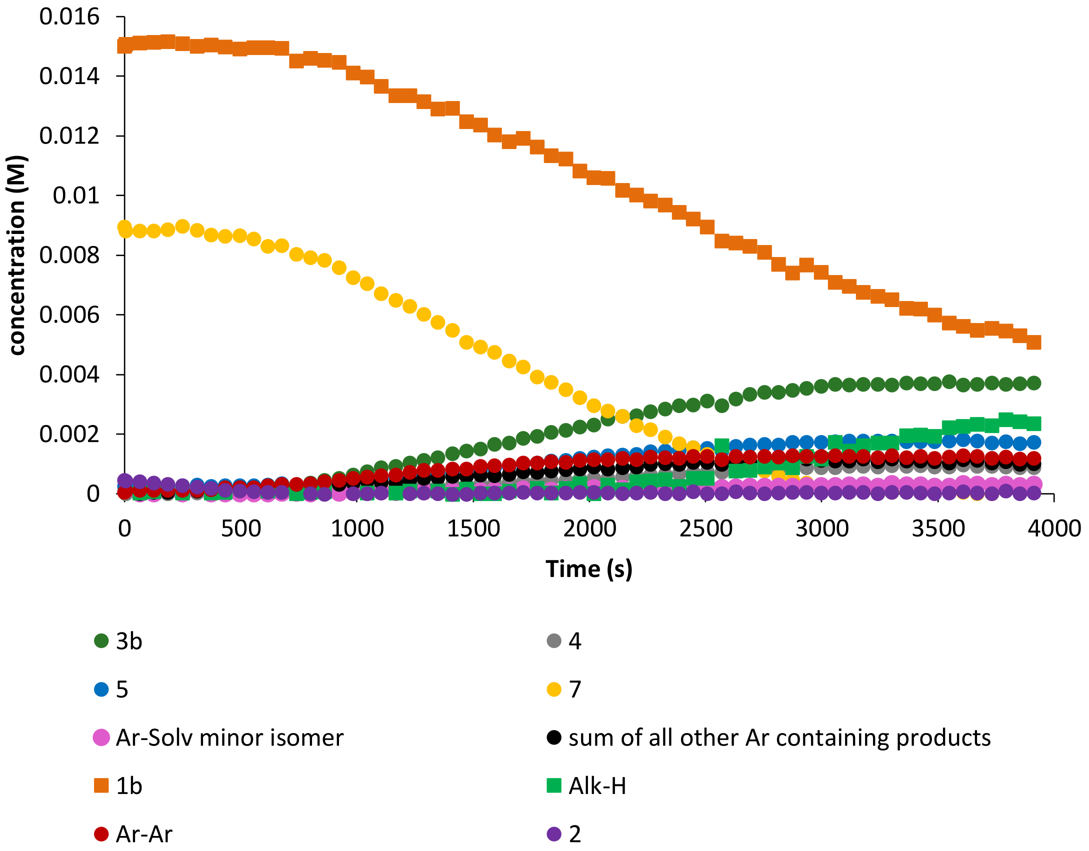

Figure S13. Kinetic profile demonstrating the stoichiometric reactivity of Ni intermediate **7** with alkyl bromide **1b**. The short induction period possibly arises from the presence of a trace quantity of quenching species in the sample that requires consumption before productive coupling can be mediated by the Ir/light. Experimental evidence for this includes temporary inhibition after addition of a large excess of **7** to an ongoing reaction. Initially small amounts of residual ArBr **2** are present in the solution; these are converted into **7** as the Ni reacts. Although small amounts of products are initially formed there is an acceleration in the reaction rate once all of **2** has been consumed. Additionally, and only under these conditions, the homocoupled biaryl product (denoted here as Ar-Ar) is observed as a significant side product. Initial conditions: [**1b**]$_0$ = 15 mM; [**2**]$_0$ = 9 mM; [Ir]$_{tot}$ = 0.5 mM; [Ni]$_{tot}$ = 0.25 mM; [(TMS)$_3$SiH]$_0$ = 10 mM; [2,6-lutidine]$_0$ = 20 mM; I_0 = 1.1 mEL^{-1}s^{-1} (420 nm).

3.4.2 Use of ArNi(L)Br complex 7 as catalyst

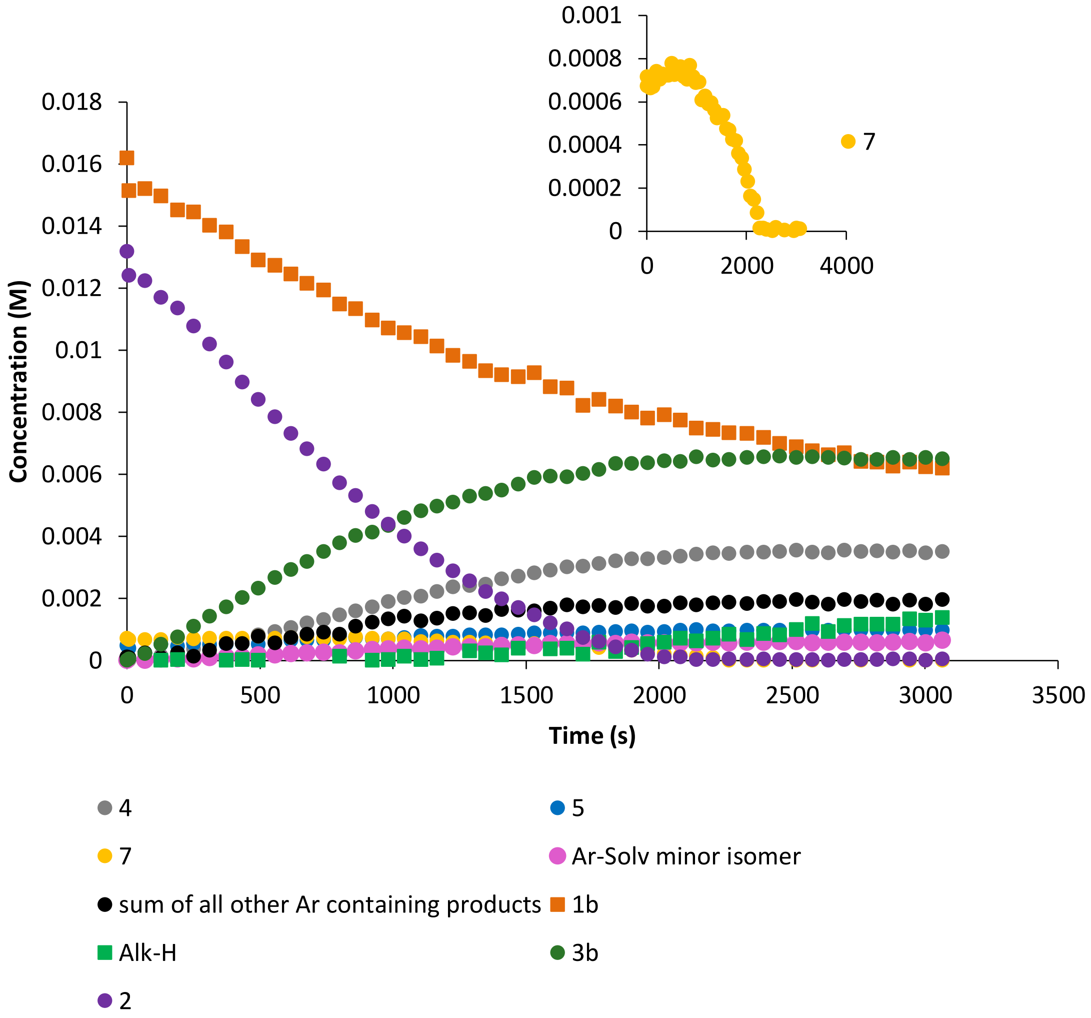

Figure S14. Kinetic profile demonstrating the ability of Ni complex **7** to act as a catalyst for the reaction. Initial conditions: [**1b**]$_0$ = 16 mM; [**2**]$_0$ = 13 mM; [Ir]$_{tot}$ = 0.5 mM; [Ni]$_{tot}$ = 0.8 mM; [(TMS)$_3$SiH]$_0$ = 10 mM; [2,6-lutidine]$_0$ = 20 mM; I_0 = 1.1 mEL^{-1}s^{-1} (420 nm).

3.5 Photochemically-Induced Ar-exchange between ArBr (2) and ArNi(L)Br (7)

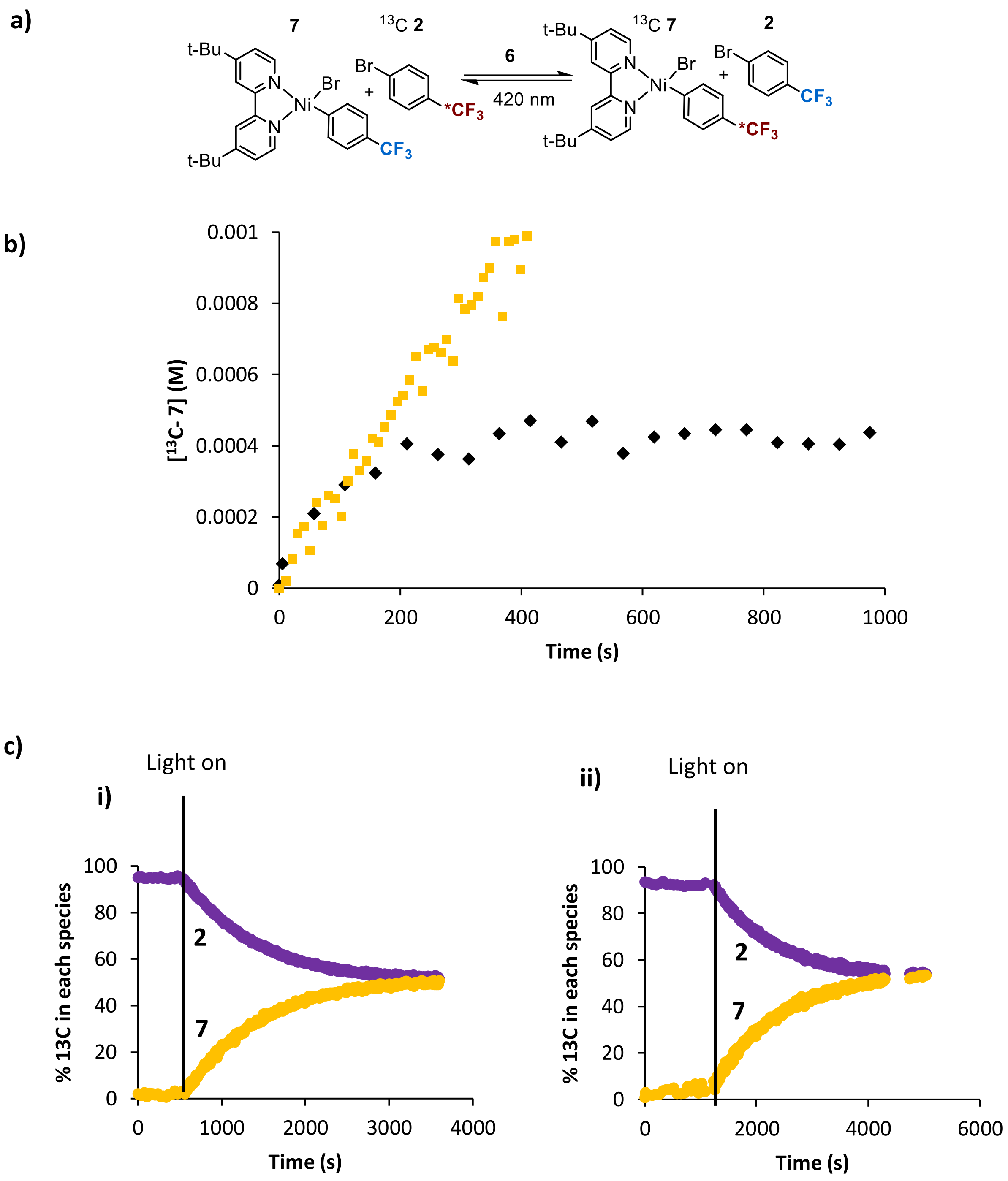

Figure S15. a) Photochemically-induced Ar-exchange of ArBr with NiAr intermediate **7**. **b**) Plot showing the concentration of ^{13}C-labelled **7** in a system starting with 1:1 unlabelled intermediate **7** (5 mmol) to photocatalyst **6** (5 mmol) and 1 equivalent of ^{13}C-labelled **2** (5 mmol) (yellow squares) and the concentration of ^{13}C-labelled **7** in a typical reaction starting with a 2:1 ratio unlabelled intermediate **7** to photocatalyst **6**, both in catalytic concentrations relative to the reaction substrates, including ^{13}C-labelled **2** (10:1 ratio of ArBr **2** / ArNiBr **7**) (black diamonds). This shows that the rate of exchange between **7** and **2** is the same in isolation as in a 'live' reaction. **c**) Graphs showing ^{13}C incorporation (%) versus time in the case where photocatalyst **6** is added i) stoichiometrically and ii) catalytically relative to the 1:1 mixture of **7** (5 mmol) and ^{13}C-labelled **2** (5 mmol). The solution was initially monitored in the absence of irradiation, and then irradiated ('light on') I_0 = 0.1 mEL^{-1}s^{-1} (420 nm) to demonstrate the Ir-photocatalysis of this exchange process.

3.6 Isotope Entrainment

In preliminary experiments, a ‘standard’ reaction of **1b** with **2** was set up as described in section S.3.1, but using 0.5 equivalents of **2**. The reaction was run to 23% conversion, and then 0.5 equivalents of ^{13}C-**2** was added to the reaction mixture. Under these conditions I_0 = 1.1 mEL^{-1}s^{-1} (420 nm) and exchange between **2** and **7** was rapid relative to the rate of data acquisition; i.e. the data-density was low, Figure S.16. Nonetheless, the short delay detected in the growth of ^{13}C into product **3b** was indicative that **7** is possibly an on-cycle intermediate.

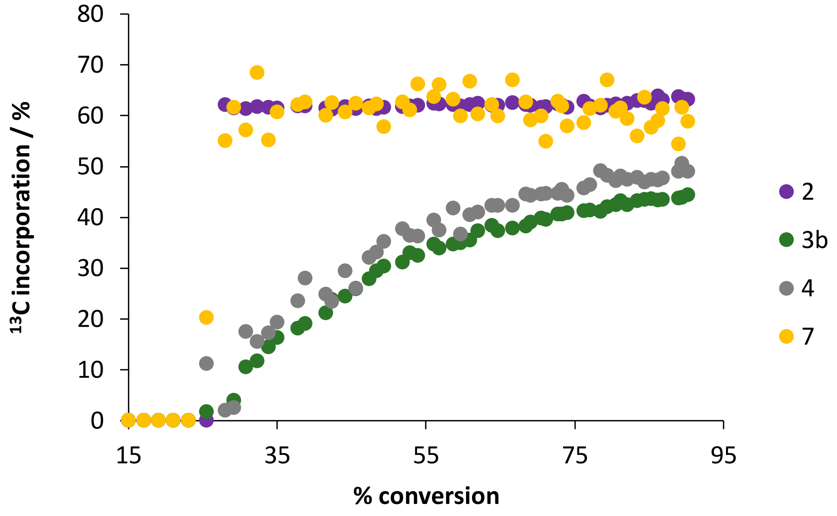

Figure S16. Preliminary isotope entrainment experiment conducted under ‘standard’ reaction conditions. [**1b**]$_0$ = 15 mM; [**2**]$_0$ = 7.5 mM; [Ir]$_{tot}$ = 0.5 mM; [Ni]$_{tot}$ = 1 mM; [(TMS)$_3$SiH]$_0$ = 10 mM; [2,6-lutidine]$_0$ = 20 mM; I_0 = 1.1 mEL^{-1}s^{-1} (420 nm). [^{13}C-**2**] = 7.5 mM added at 23 % total conversion of [**2** + ^{13}C-**2**]

To facilitate a more-detailed analysis of the system, we initiated the reaction using unlabelled complex **7** (11 mol%) together with labelled aryl substrate, ^{13}C-**2**, and used the method of 'periodic activation'[S13] to enable higher data density to be acquired within the early stages of the reaction evolution. In order to minimize pre-equilibration of ^{13}C 4-bromobenzyl trifluoride (^{13}C-**2**) and **7**, the solution was assembled and used immediately. The amberized NMR tube with 280 uL of reaction solution containing an empty quartz coaxial insert was assembled in the glovebox. The sample was removed from the glovebox, a bare fibre (treated as described above in section 1) was placed inside the insert and secured in place, and the entire assembly was lowered into the spectrometer probe. After the usual shimming routine a series of ^{19}F NMR spectra were taken using the Bruker AU program ‘multi_zgvd’, which takes a series of NMR spectra at a set temporal interval. The standard pulse sequence was modified such that a consistent TTL signal usually emitted by the spectrometer was switched off during NMR acquisition. The LED driver was programmed such that it initially responded to a change in TTL conditions and thereafter followed the behaviour of the LED signal; i.e. there was initially no irradiation of the solution, which persisted until the conclusion of the first NMR acquisition; at this point, the TTL signal resumed, and the solution was irradiated until cessation of this signal at the beginning of the pulse sequence of the next NMR acquisition, at which point the irradiation was ceased and resumed once NMR acquisition had completed. Each NMR

acquisition consisted of 32 scans and took about 3.5 minutes to acquire. The solution was irradiated at low power (5% of full LED capacity; 0.05 $mEL^{-1}s^{-1}$) for 30 seconds between each acquisition. Under these conditions it took 19 hours to reach just over 40% conversion.

The acquired spectra were processed in mestrenova and excel. The conversion (%) was calculated at each time point from the sum of the remaining concentrations of $[\mathbf{2}]_t$ and $[^{13}C\text{-}\mathbf{2}]_t$ and their initial concentrations $[\mathbf{2}]_0$ and $[^{13}C\text{-}\mathbf{2}]_0$ as defined by the spectra taken immediately before beginning the periodic irradiation of the solution. The ^{13}C incorporation (%) was calculated at each time point from the relevant concentrations. The signals from the two major side products (**4** and **5**) overlapped with trace quantities of other species, which made their concentration at *t*=0 appear non-zero. In such cases each concentration was normalised against this value to allow meaningful comparison of the rates of formation of ^{12}C and ^{13}C labelled species. To minimise scatter in the plots resulting from noise in the spectra a 'filter' was applied to the final %^{13}C data which eliminated all data points where at least one of the apparent concentrations was below a minimal threshold. The data and model are shown in figure S17.

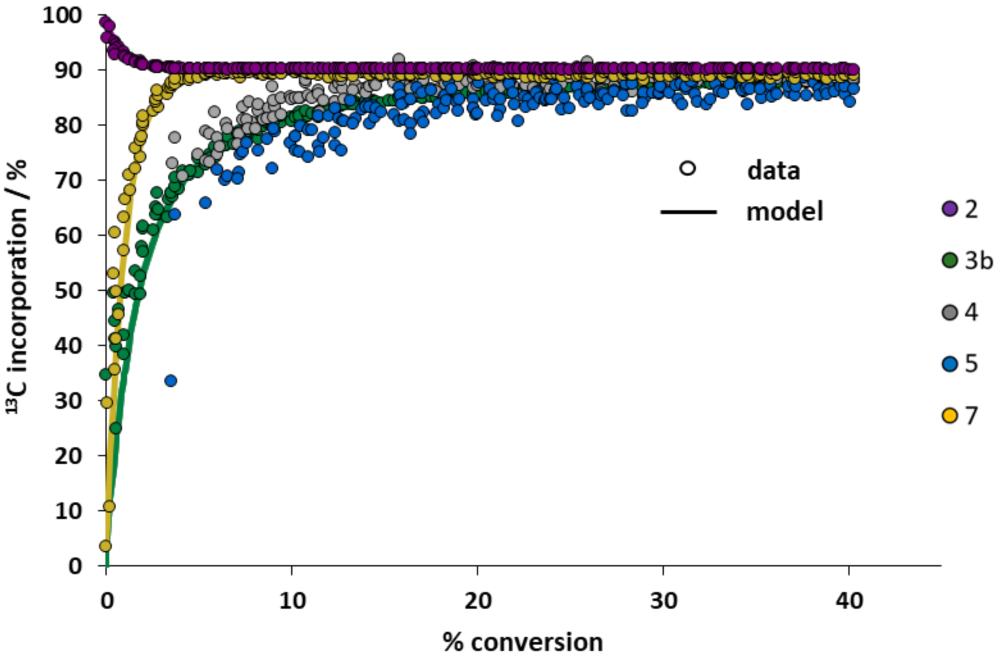

Figure S17. Isotope entrainment analysis, showing both the experimentally recorded % incorporation of ^{13}C in each species against reaction conversion, and the predicted plot (solids lines) from the minimal kinetic model (see Section S5) when the recycling ratio ($1/K_{f3} = k_{RE}/k_C$) is set at 9.3. Initial conditions: $[\mathbf{1b}]_0$ = 13 mM; $[^{13}C\text{-}\mathbf{2}]_0$ = 7 mM; $[Ir]_{tot}$ = 0.5 mM; $[Ni]_{tot}$ = 0.8 mM; $[(TMS)_3SiH]_0$ = 10 mM; $[2,6\text{-lutidine}]_0$ = 20 mM; I_0 = 0.05 $mEL^{-1}s^{-1}$ (420 nm), alternated with I_0 = 0 (^{19}F NMR spectral acquisition).

In further experiments, the effect of variation in the Ni concentration was explored, Table S1. Two of the analyses (entries 2 and 5) were conducted using an identical procedure to that detailed above, i.e. ^{19}F NMR spectroscopic determination of $^{13}CF_3$ (%) in the substrate (**2**), ArNi intermediate (**7**) and in the products **3**, **4** and **5**. Two of the analyses (entries 1 and 4)

were conducted without silane and alkylbromide, thus requiring determination of $^{13}CF_3$ (%) in the substrate (**2**), ArNi intermediate (**7**) and the solvent-coupling product Ar-DME (**4**).

Entry	Ni mol %	[^{13}C-ArBr] /mM	[ArNi(L)Br] /mM	[2,6-lutidine] /mM	[$(TMS)_3SiH$] /mM	[RBr, **1b**] /mM	k_{RE}/k_C ($1/K_{f3}$)
1	0.02	17.8	0.3	20	0	0	12.3[a]
2	0.05	11.2	0.6	20	10	17	2.3[b]
3	0.11	7.0	0.8	20	10	13	9.3[b,c]
4	0.13	14.8	1.9	20	0	0	37.9[a]
5	0.20	11.5	2.3	20	10	17	92.9[b]

[a] Silane-free conditions giving solvent coupling only, with rate-ratio estimated by fitting %^{13}C in **2**, **4**, and **7** as a function of conversion **2** analyzed by in situ LED-^{19}F-NMR, to model shown in Section S.5. [b] Normal conditions giving cross-coupling product, rate-ratio estimated by fitting %^{13}C in **2**, **3**, **4**, **5**, and **7** as a function of conversion **2** analyzed by in situ LED-^{19}F-NMR, to model shown in Section S.5. [c] Data shown in Figure S7.

Table S1 - Additional isotope entrainment analyses, showing the recycling ratio ($1/K_{f3} = k_{RE}/k_C$) calculated from % incorporation of ^{13}C in various species as a function of reaction conversion using the minimal kinetic model (see Section S5). $I_0 = 0.05$ mEL^{-1}s^{-1} (420 nm), alternated with $I_0 = 0$ (^{19}F NMR spectral acquisition).

During these analyses it was found that the formal exchange of aryl groups between **2** and **7** relative to product generation (i.e. the recycling ratio, $1/K_{f3} = k_{RE}/k_C$) was increased at the raised nickel concentrations, Table S1. Control experiments conducted in amberized NMR tubes without 420 nm LED irradiation ($I_0 = 0$) did not lead to a significant increase in the slow background 'dark' exchange process. Overall, the data shows that use of high Ni-concentrations under the photocatalyzed Ir/Ni coupling conditions, will increase non-productive recycling ($1-f_3$) thus augmenting the reduced quantum efficiency arising from competing light-absorption by Ni ($1-f_1$).

3.7 Systematic Variation of Individual Components

All solutions were prepared as described in section S3.1. For each series in which the indicated component was varied while leaving all other components equal, the experiments were conducted back-to-back on the same day in a randomised order using pre-irradiation reaction mixtures assembled in parallel in the glove box from identical stock solutions. The overlaid temporal concentration profiles for each set of component concentration variations that are summarized in Figure S18 are presented in Sections 3.7.1 to 3.7.7. In some cases, additional 'concentration-versus-concentration' plots are presented.

(**a**)

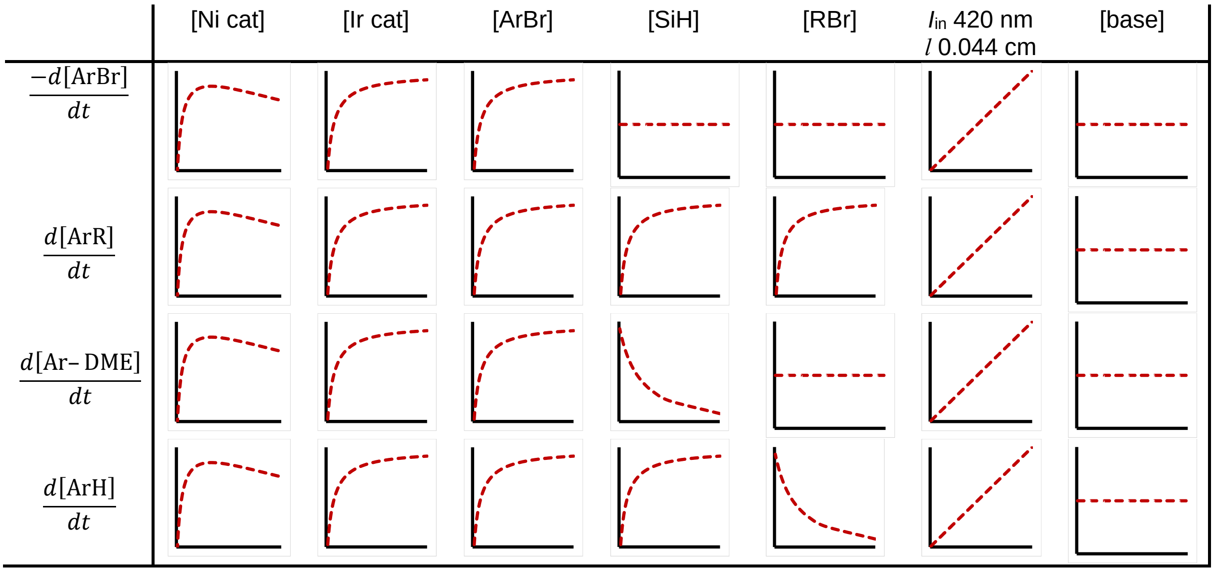

(**b**)

$$f_4 = \frac{d[\mathrm{ArR}]+d[\mathrm{ArH}]}{d[\mathrm{ArDME}]+d[\mathrm{ArR}]+d[\mathrm{ArH}]} \approx \left[\frac{1}{1+\frac{[\mathrm{DME}]}{K_{f4}[\mathrm{SiH}]}}\right]$$

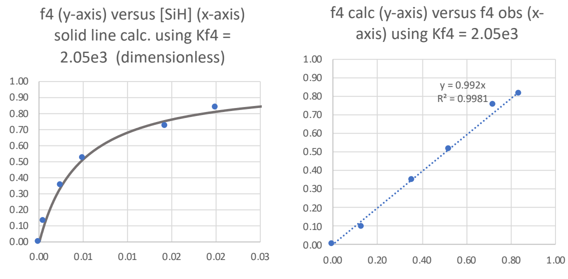

(**c**)

$$f_5 = \frac{d[\mathrm{ArR}]}{d[\mathrm{ArH}]+d[\mathrm{ArR}]} \approx \left[\frac{1}{1+\frac{1}{K_{f5}[\mathrm{RBr}]}}\right]$$

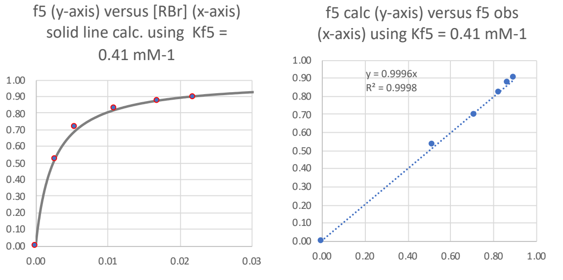

Figure S18. (a) Schematic effect of each reaction component on the rates of decay / growth of the major species present in the reaction mixture, determined by in situ LED-^{19}F NMR spectroscopy, with low concentrations of Ni, Ir, reactants and reagents, and short light pathlength **(b)** Empirical analysis of selectivity factor K_{f4} for partitioning (f_4) of ArBr into Ar-R + ArH versus Ar-DME. Relative rates used to calculate f_4 are the average values from Fig S3.7.5 **(c)** Empirical analysis of selectivity factor K_{f5} for partitioning (f_5) of ArBr into Ar-R versus Ar-H. Relative rates used to calculate f_5 are the average values from Fig S3.7.4

3.7.1 Variation of Ni catalyst loading

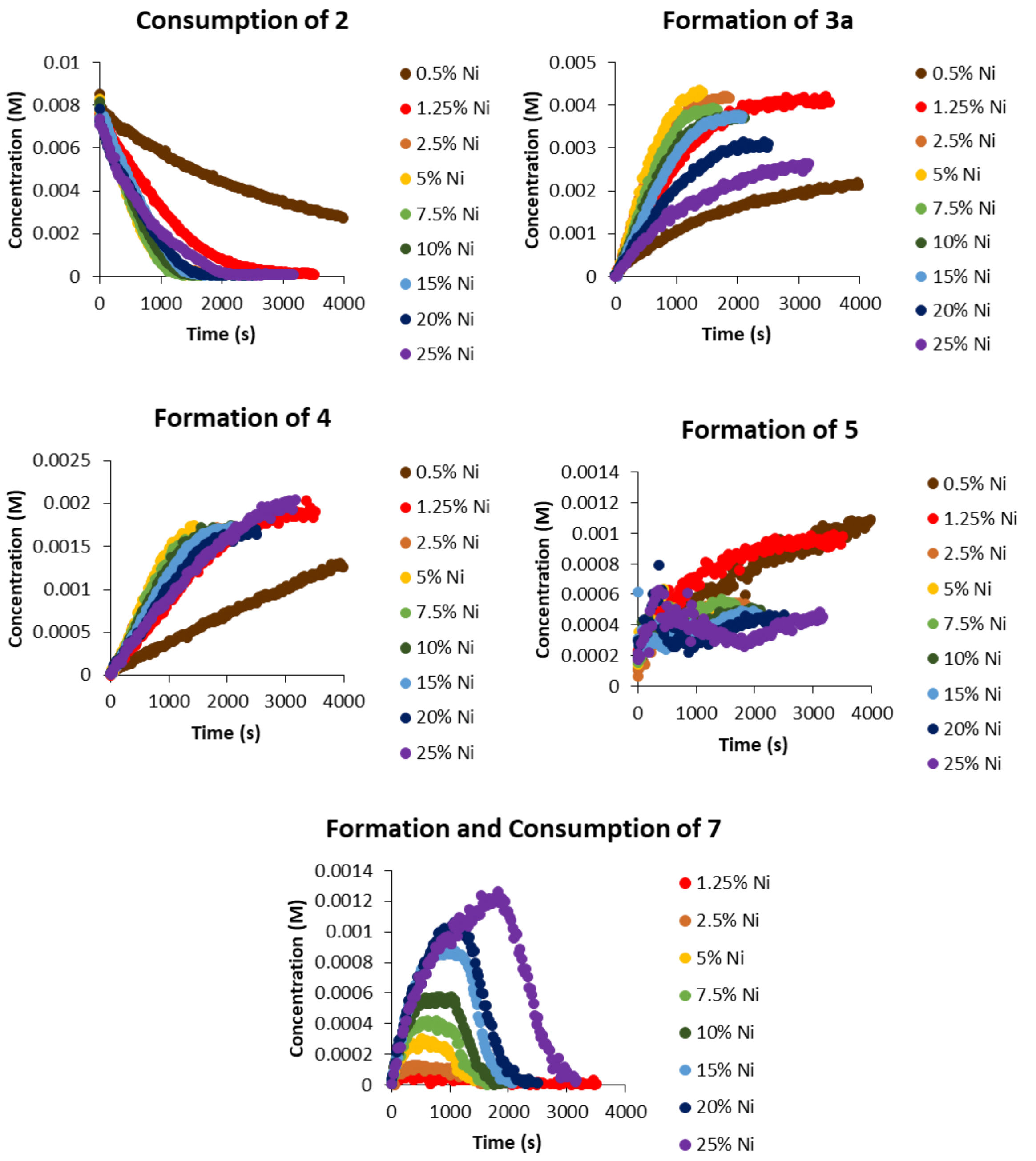

Figure S19. Overlaid spectra detailing the effect of varying starting Ni catalyst loading on the concentration of the major species present during the reaction against time. Each colour of data represents a different initial Ni loading. Initial concentrations of all other components, which are kept constant: [**1a**]$_0$ = 15 mM; [**2**]$_0$ = 10 mM; $[Ir]_{tot}$ = 0.5 mM; $[(TMS)_3SiH]_0$ = 10 mM; [2,6-lutidine]$_0$ = 20 mM; I_0 = 1.1 mEL^{-1}s^{-1} (420 nm).

3.7.2 Variation of Ir photocatalyst 6 loading

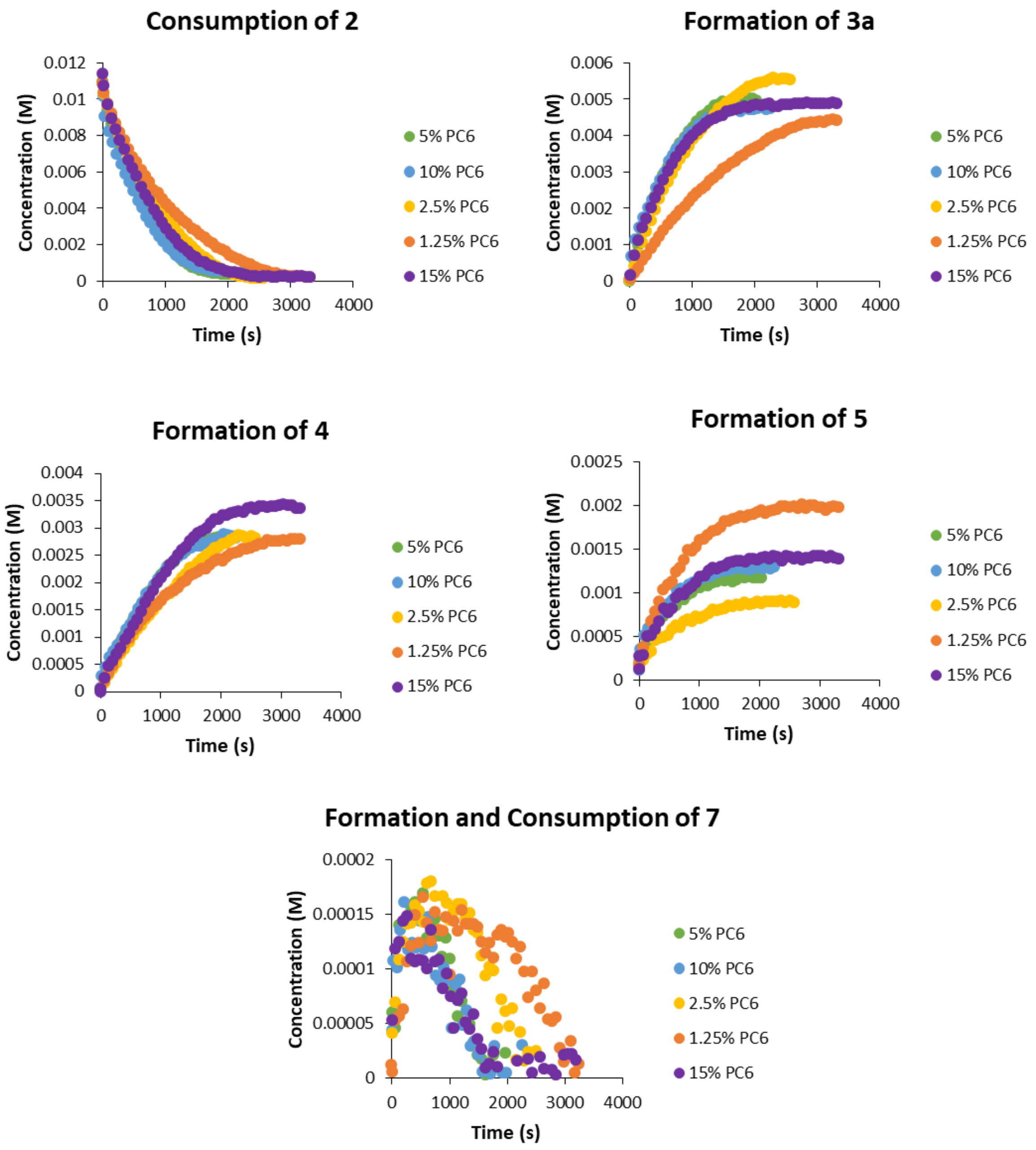

Figure S20. Overlaid spectra detailing the effect of varying starting photocatalyst loading on the concentration of the major species present during the reaction against time. Each colour represents a different initial photocatalyst loading. Initial concentrations of all other components, which are kept constant: $[\mathbf{1a}]_0$ = 15 mM; $[\mathbf{2}]_0$ = 10 mM; $[Ni]_{tot}$ = 0.25 mM; $[(TMS)_3SiH]_0$ = 10 mM; $[2,6\text{-lutidine}]_0$ = 20 mM; I_0 = 1.1 mEL^{-1}s^{-1} (420 nm).

3.7.3 Variation of ArBr 2 loading

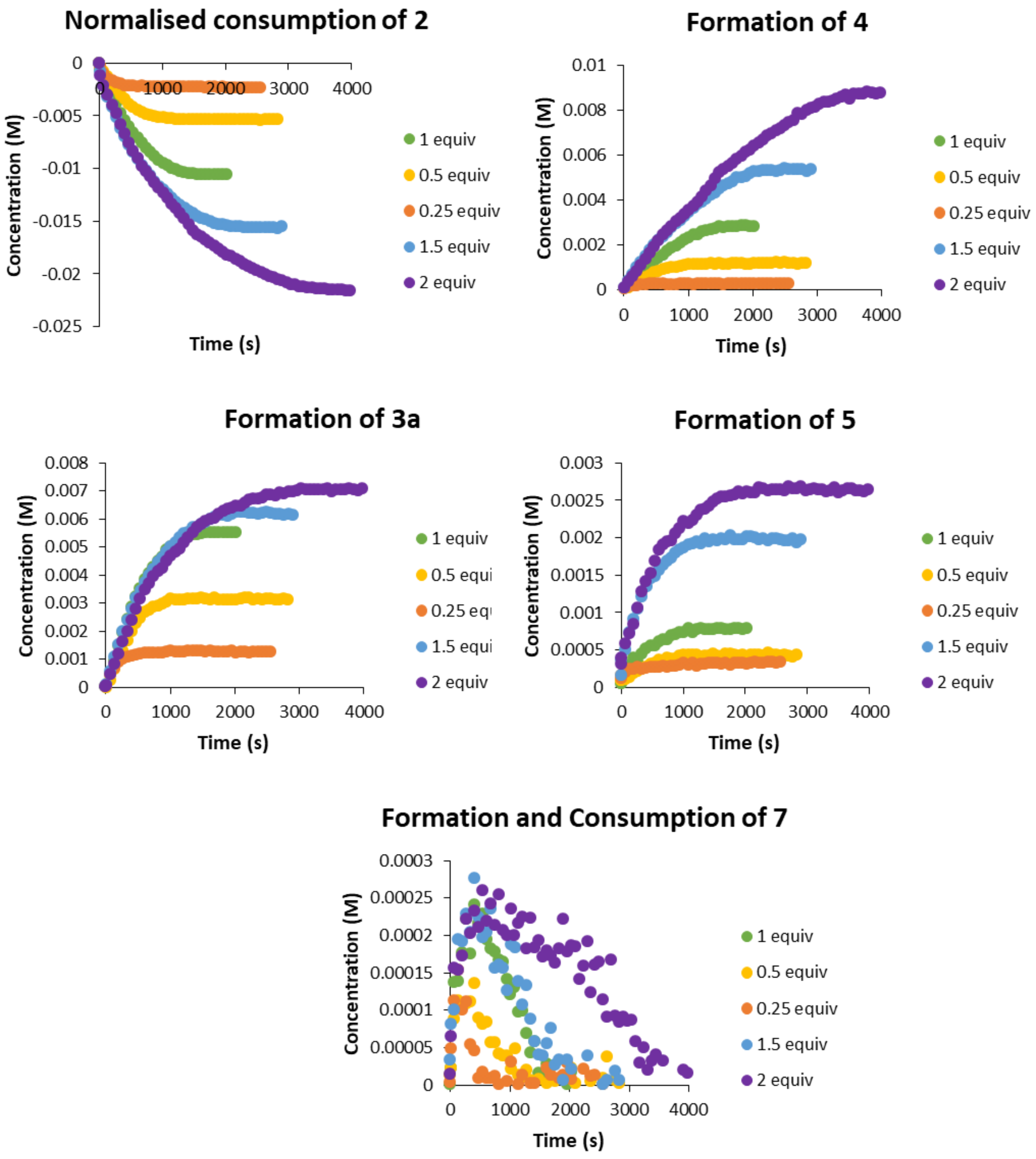

Figure S21. Overlaid spectra detailing the effect of varying starting ArBr loading on the concentration of the major species present during the reaction against time. Each colour represents a different initial ArBr loading. In the case of the plot showing consumption of ArBr **2** the data has been normalised such that the starting concentration of **2** is the same in each case, to more obviously visualise the relative behaviour of each dataset. Initial concentrations of all other components, which are kept constant: [**1a**]$_0$ = 15 mM; [Ir]$_{tot}$ = 0.5 mM; [Ni]$_{tot}$ = 0.25 mM; [(TMS)$_3$SiH]$_0$ = 10 mM; [2,6-lutidine]$_0$ = 20 mM; I_0 = 1.1 mEL^{-1}s^{-1} (420 nm).

3.7.4 Variation of Alkyl Bromide 1a Loading

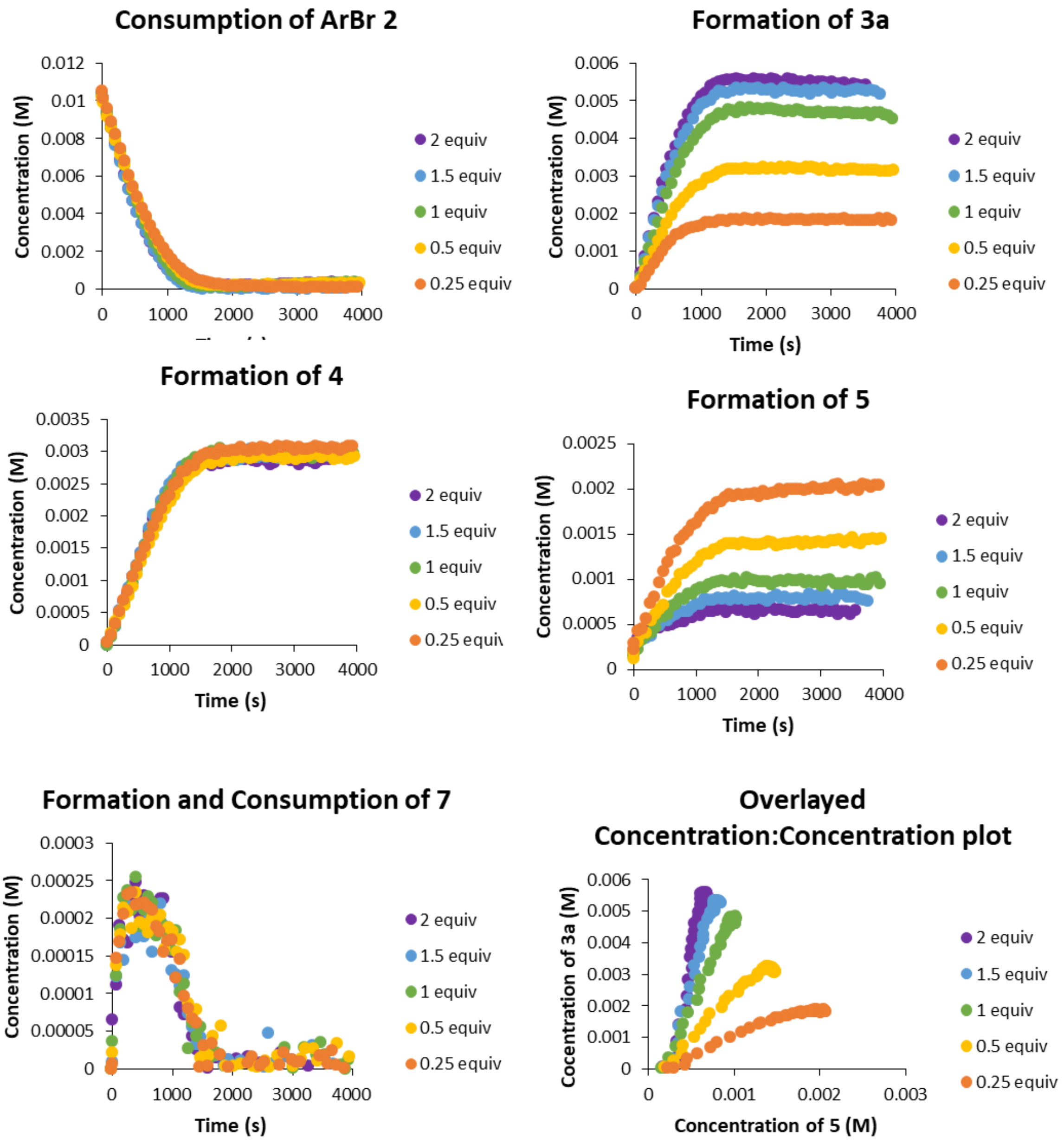

Figure S22. Overlaid spectra detailing the effect of varying starting silane loading on the concentration of the major species present during the reaction against time. Each colour represents a different initial silane loading. Included also is a plot of the concentration of **3a** plotted against the concentration of **5**, demonstrating the lack of secondary partition between these two substrates that could have been obscured by the more obvious partition between them and **4**. Initial concentrations of all other components, which are kept constant: [**1a**]$_0$ = 15 mM; [**2**]$_0$ = 10 mM; [Ir]$_{tot}$ = 0.5 mM; [Ni]$_{tot}$ = 0.25 mM; [2,6-lutidine]$_0$ = 20 mM; I_0 = 1.1 mEL^{-1}s^{-1} (420 nm).

3.7.5 Variation of Silane Loading

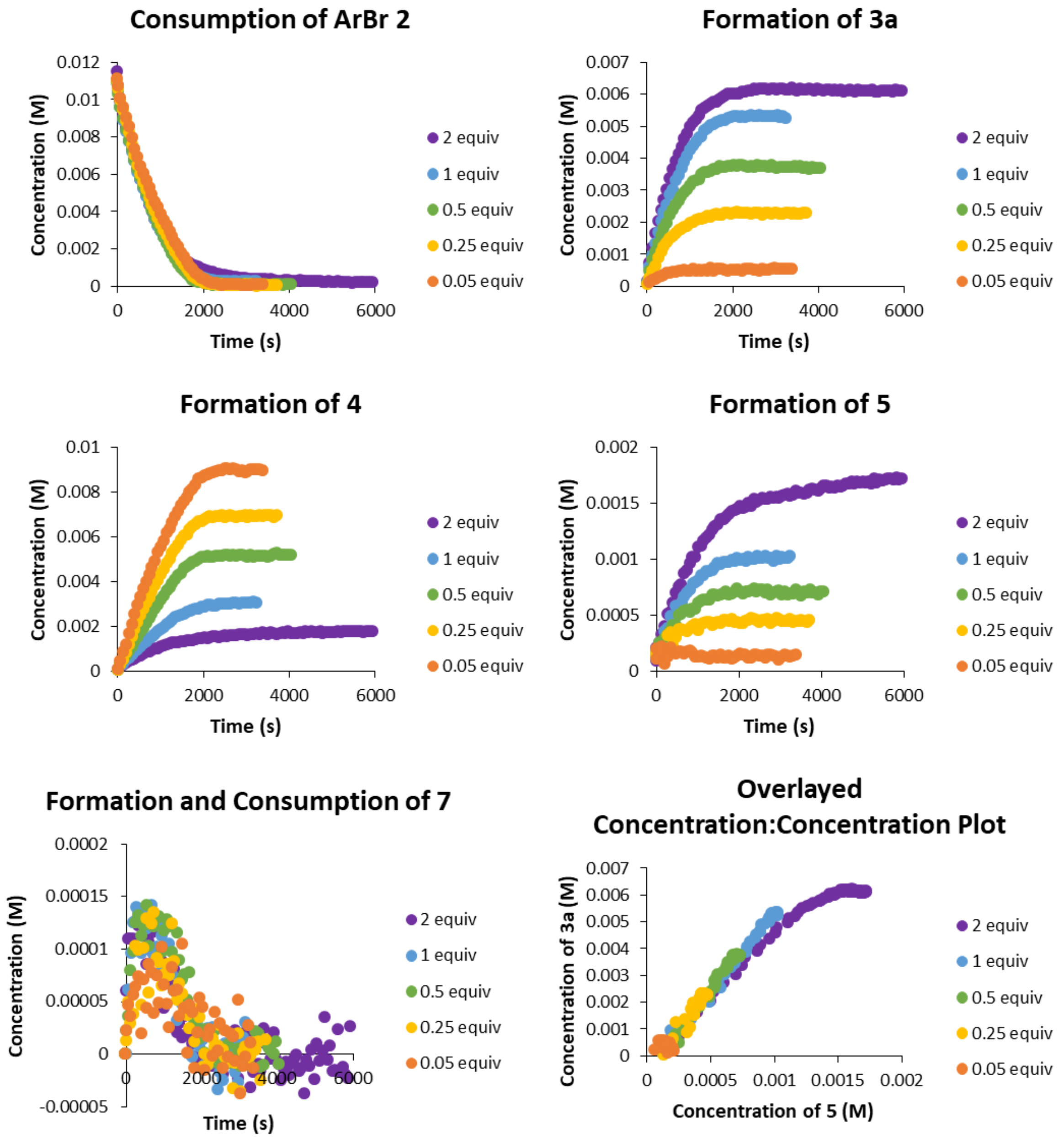

Figure S23. Overlaid spectra detailing the effect of varying starting silane loading on the concentration of the major species present during the reaction against time. Each graph includes only one species, with each colour of data representing a different initial silane loading. Included also is a plot of the concentration of **3a** plotted against the concentration of **5**, demonstrating the lack of secondary partition between these two substrates that could have been obscured by the more obvious partition between them and **4**. Initial concentrations of all other components, which are kept constant: $[\mathbf{1a}]_0$ = 15 mM; $[\mathbf{2}]_0$ = 10 mM; $[Ir]_{tot}$ = 0.5 mM; $[Ni]_{tot}$ = 0.25 mM; $[2,6\text{-lutidine}]_0$ = 20 mM; I_0 = 1.1 $mEL^{-1}s^{-1}$ (420 nm).

3.7.6 Variation of Base Loading

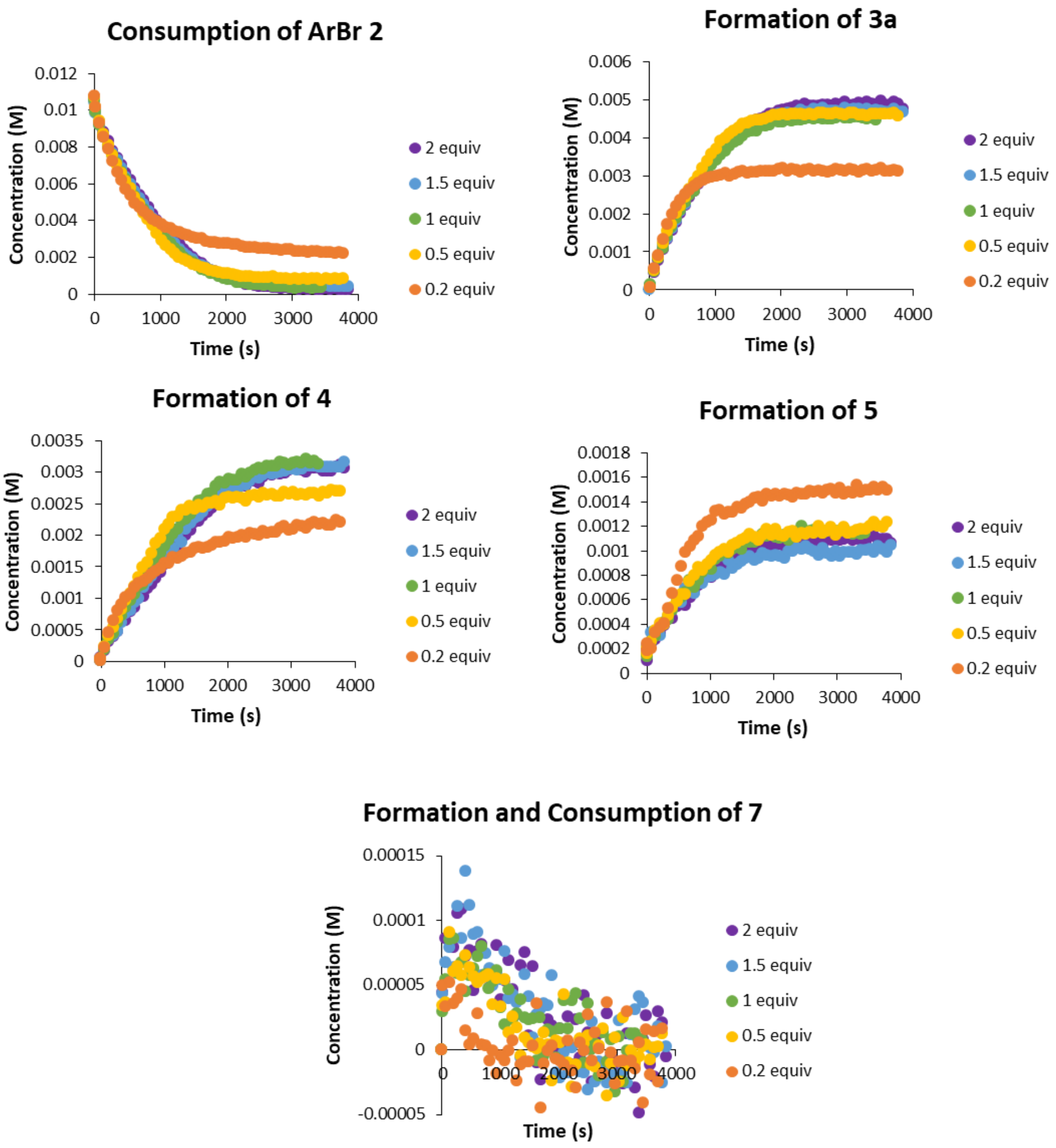

Figure S24. Overlaid spectra detailing the effect of varying starting base loading on the concentration of the major species present during the reaction against time. Each colour represents a different initial base loading. Initial concentrations of all other components, which are kept constant: $[\mathbf{1a}]_0$ = 15 mM; $[\mathbf{2}]_0$ = 10 mM; $[Ir]_{tot}$ = 0.5 mM; $[Ni]_{tot}$ = 0.25 mM; $[(TMS)_3SiH]_0$ = 10 mM; I_0 = 1.1 mEL^{-1}s^{-1} (420 nm).

3.7.7 Variation of Light Intensity

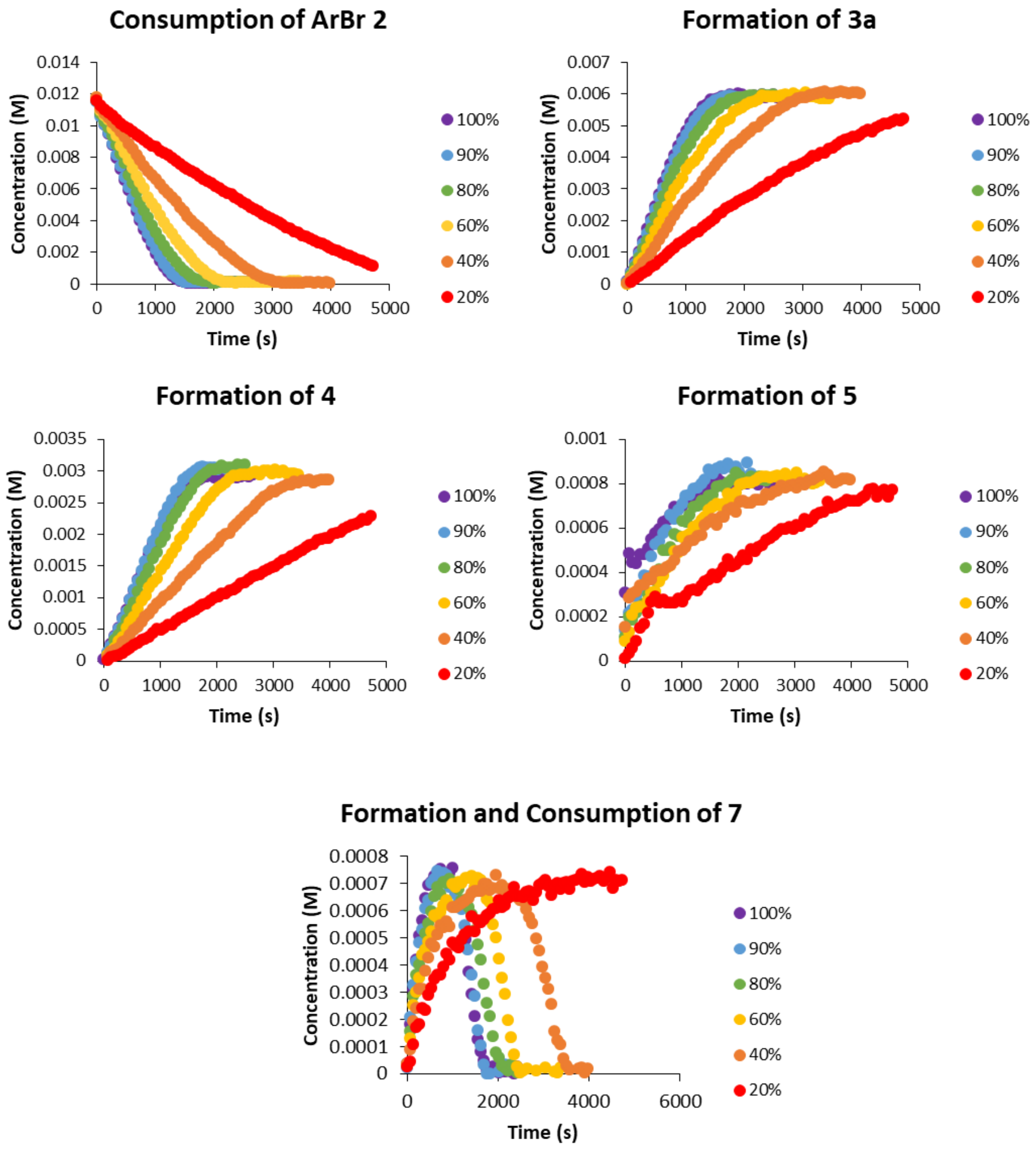

Figure S25. Overlaid spectra detailing the effect of varying light intensity, given as a percentage of the maximum power output of the system, on the concentration of the major species present during the reaction against time. Each graph includes only one species, with each colour of data representing a different light intensity. Initial concentrations of all other components, which are kept constant: [**1a**]$_0$ = 15 mM; [**2**]$_0$ = 10 mM; $[Ir]_{tot}$ = 0.5 mM; $[Ni]_{tot}$ = 0.25 mM; $[(TMS)_3SiH]_0$ = 10 mM; [2,6-lutidine]$_0$ = 20 mM.

3.8 Comparative concentration-concentration plot showing independence of silane-mediated and Alk-Br mediated partitions

The independence of the silane-mediated partition of the identity of the alkyl bromide can be further be seen by plotting the concentration of solvent-coupling product **4** against the sum of other products of the reaction for two reactions conducted with two distinct alkyl bromides:

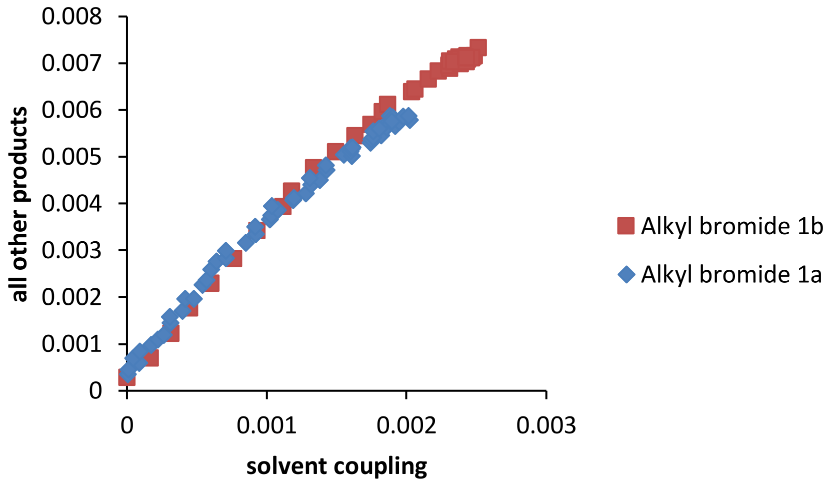

Figure S26. Overlayed concentration-concentration plots demonstrating the independence of the silane-mediated partition on the identity of the alkyl bromide. This provides further evidence for both the partitioning scheme in general and that the silane-mediated partition is ‘upstream’ of the AlkBr-mediated partition.

3.9 DME Solvent $^1H/^2H$ KIE

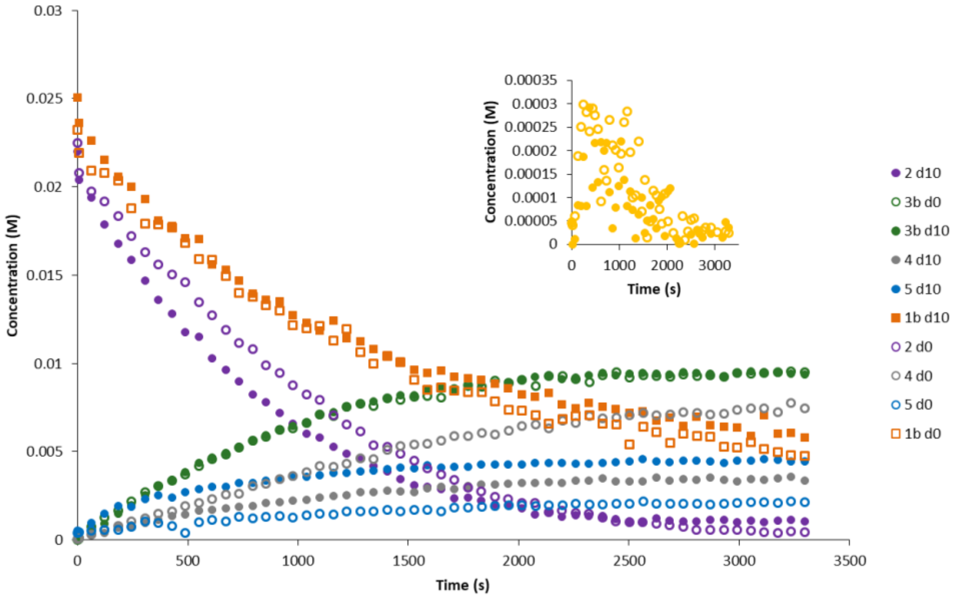

Figure S27. Overlayed concentration-time graphs for two otherwise-identical reactions conducted in d_0-DME (open shapes) and d10-DME (filled shapes) respectively. Kinetic modelling of the solution gives the solvent KIE at f_4 as 2.2 whereas analysis via concentration-concentration plots (see figure S28) gives the solvent KIE at f_4 as 1.9. An apparent solvent KIE at f_5 can be seen to primarily arise from an initial burst of protodebromination product **5** at the start of the reaction in d10-DME. Analysis of the endpoints of these reactions by NMR and MS (EI and ESI) provided no evidence for the proton in protodebrominated product **5** originating from the solvent. Initial conditions: [**1b**]$_0$ = 25 mM; [**2**]$_0$ = 20 mM; [Ir]$_{tot}$ = 0.5 mM; [Ni]$_{tot}$ = 0.25 mM; [(TMS)$_3$SiH]$_0$ = 20 mM; [2,6-lutidine]$_0$ = 40 mM; I_0 = 1.1 mEL^{-1}s^{-1} (420 nm).

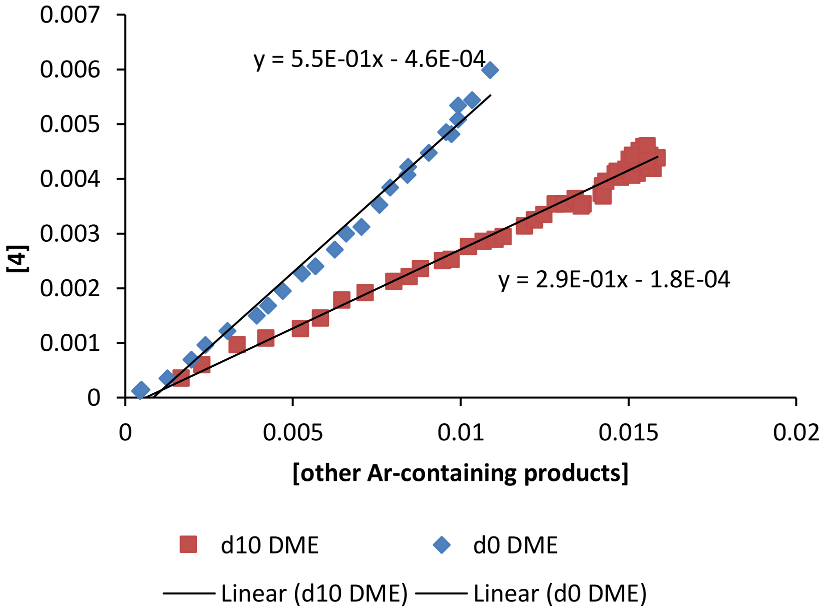

Figure S28. Overlaid concentration-concentration plots of solvent-coupled side product **4** against all other products, for identical reactions conducted in d_0-DME (blue diamonds) and d_{10}-DME (red squares). The partitioning f_4 can be taken as the gradient of each respective graph, and calculation of KIE = f_4(H)/f_4(D) yields a value for the solvent KIE of 1.9.

4. Determination of the Absolute Photon Flux of the LED-NMR Apparatus using Ferrioxolate Actinometry

All of the below manipulations were conducted in a dark room under red illumination, and the experiment was designed following literature precedent.[S15]

Sulfuric acid (14 mL, 17.8 M) was diluted up to 500 mL to form a 0.5 M aqueous solution of sulfuric acid. Sulfuric acid (10 mL, 0.5 M) was diluted up to 100 mL to form a 0.05 M aqueous solution of sulfuric acid. Potassium ferrioxalate trihydrate (1.85 g, 3.76 mmol) was dissolved in sulfuric acid (25 mL, 0.05 M). Sodium acetate (22.6 g, 277 mmol, 49 eq.) was dissolved in sulfuric acid (100 mL, 0.5M). 1,10-phenanthroline (1.02 g, 5.67 mmol. 1 eq.) was then added.

An aliquot of the potassium ferrioxalate solution (0.28 mL) was added to an amberized NMR tube, into which was then placed the LED-NMR insert. The solution was then illuminated for the required time at 420 nm, at the highest intensity setting of the LED driver. 10 µL of this solution was then added to an aliquot of the sodium acetate + phenanthroline solution (5 mL) containing a magnetic stirrer bar. The mixture was left to stir for one hour in the dark. A UV-Vis spectrum was then taken of this solution, in a 1 cm pathlength cuvette, and the absorbance at 510 nm used to calculate the moles of generated Fe^{2+} in the illuminated solution, as shown below:

$$[Fe(C_2O_4)_3]^{3-} \xrightarrow{h\nu} Fe^{2+} + 2(C_2O_4^{2-}) + C_2O_4^{-}$$

$$Fe^{2+} + 3(\text{phen}) \longrightarrow [Fe(\text{phen})_3]^{2+}$$

From the Beer-Lambert law:

$$[(\mathrm{Fe(phen)}_3)^{2+}] \text{ in the 5 mL solution} = \frac{\Delta A}{\varepsilon l}$$

Where:

ΔA = absorbance at 510 nm relative to a non-irradiated control sample

ε = extinction coefficient of $[Fe(phen)_3]^{2+}$ at 510 nm (11 100 L cm^{-1} mol^{-1})[S14,S15]

l = path length of the light through the solution (1 cm)

Thus,

$$n((\mathrm{Fe(phen)}_3)^{2+} \text{ in the 5 mL solution}) = \frac{\Delta A \times 0.005\ L}{1\ cm \times 11100\ Lcm^{-1}mol^{-1}}$$

The number of moles, n, of Fe^{2+} that is generated in the 0.28 mL solution contained in the NMR tube after illumination is thus:

$$n = n((Fe(phen)_3)^{2+} \times \frac{280}{10}$$

The data are given in Table S3, and plotted in Figure S29.

Time (s)	Absorbance at 510 nm	Δ Absorbance	$n(Fe^{2+})$ (mol) ($\times 10^{-6}$)
0	0.149	0	0
10	0.446	0.297	3.74
20	0.643	0.494	6.23
30	0.815	0.666	8.40
40	0.929	0.781	9.84
50	1.00	0.855	10.8

Table S3 - moles, n, of Fe^{2+} generated in the NMR tube after illumination.

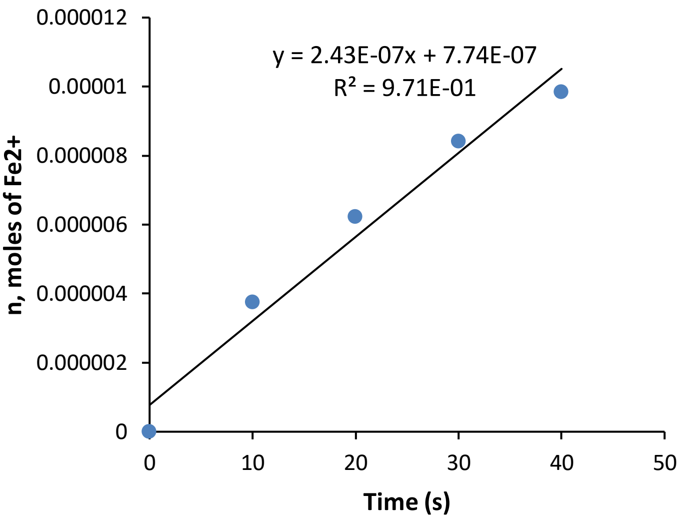

Figure S29. Data from ferrioxolate actinometry, see text for full details.

Thus $n(Fe^{2+})$ generated per second of irradiation is approximately 2.43 $\times 10^{-7}$ mol/s.

Given that ϕ ($K_3[Fe(C_2O_4)_3]$) at 420 nm = 1.12 (from literature[S16])

And that:

$$\phi = \frac{n(product)}{n(photons)}$$

Under these conditions:

$$n(\text{photons absorbed by } K_3[\text{Fe}(C_2O_4)_3]) = \frac{n(\text{product})}{\Phi} = \frac{2.43 \times 10^{-7}}{1.12} = 2.17 \times 10^{-7}\ mol\ s^{-1}$$

The total incident light intensity can then be calculated from the Beer-Lambert law:

$$n(\text{photons absorbed by } K_3[\text{Fe}(C_2O_4)_3]) = I_0(1 - 10^{-\varepsilon lc})$$

Where:

I_0 = the number of incident photons

ε = extinction coefficient at 420 nm.

l = path length (measured as 0.44 mm - in the LED-NMR apparatus)

c = concentration of $K_3[Fe(C_2O_4)_3]$ (0.15 M)

From the literature, logε of the $K_3[Fe(C_2O_4)_3]$ at 420 nm is approximately 1.9.[S16] Thus: $\varepsilon = 10^{1.9} = 80\ M^{-1}\ cm^{-1}$

And therefore:

$$I_0 \approx \frac{2.17 \times 10^{-7}}{1 - 10^{-(80\times 0.044 \times 0.15)}} \approx 0.31\ \mu\text{E/s}$$

and thus, I_{in}, the average incident photon flux per unit volume in the solution surrounding the light source within the LED-NMR setup is:

$$I_{in} \approx \frac{3.1 \times 10^{-7}}{0.28 \times 10^{-3}} = 1.1 \times 10^{-3}\ \text{mol photon L}^{-1}\text{s}^{-1}$$

$$I_{in} \approx 1.1\ \text{mEL}^{-1}\text{s}^{-1}$$

5. Kinetic Simulations

5.1 Model Development

In the discussion below (Section 5.2), we focus on kinetic simulations using our final model (Figure S30). *The model is predominantly 'chemically agnostic' and is presented as a phenomenological framework for future evaluation and evolution*. It evolved from the exploration of a diverse and extensive series of kinetic dependencies. The process was guided by visual analysis of the general effects of the rate and selectivity on the process parameters (section S 3.7) and evolved in a predominantly heuristic manner. A number of models based on literature proposals (Section S6) were tested but later eliminated. Throughout, the primary goal was a reasonably robust *minimal* kinetic model that accounts for the temporal evolution of [**2**], [**3**], [**4**], [**5**], and [**7**], based on input initial values for [**1**], [**2**], $[Ni]_{tot}$, $[Ir]_{tot}$, $[(TMS)_3SiH]$, and the photon intensity (I_{in}), whilst allowing for reasonable experimental error. Competing processes consuming **1**, e.g. RBr $\rightarrow$ RH, were minor and not included in the model.

Notes

(a). The conversion of light absorbed into turnover was modelled by introduction of a nominal chemical species, 'hv' that reacts as a catalyst to activate the Ir ground state complex **6,** eq S1 and eq. S2. The rate of decay of Ir* to **6** was set such that [**6**] >> [Ir*]. Traces of progressive Ir catalyst degradation or inhibition are observed experimentally (by ^{19}F NMR) and are required in the model (k_{dec}); this process may be related to a slow Ir-redox re-speciation, see Section S8. The model does not include 'hv' in a direct or indirect catalyst activation step. However, it is evident from the correlation of k_{act} with $[h\nu]$ that light is a required component.

(b) The $[h\nu]$ concentration requires modification in a manner that not only mimics Beer-Lambert absorption but also the additional empirically-detected (but mechanistically unidentified) Ir/Ni rate-attenuation processes. Attempts to include these in f_2 (Ir) and f_3 (Ni) with the $[h\nu]$ concentration modulated only by Beer-Lambert absorption (f_1) were successful, but the overall more complex model gave similar or slightly poorer fits than the simpler one, where $[h\nu]$ are fitted, see Table S2, entries 24-28 (Ir) and16-23 (Ni).

(c). The partition f_2 (equation S4) for Ir* quenching (mechanisms undefined) by ArNiBr complex **7**, versus inherent (k_r) relaxation was set using $K_{f2} = 1.1\ mM^{-1}$. This would suggest that f_{2max} is approached at $[Ni]_0$ >12 mM. However, experimentally (Figure S19) when $[Ni]_0$ >1 mM, catalyst pre-activation is not complete before all of the ArBr has been consumed.

(d). Ar-commitment (k_C) versus ArBr recycling (k_{RE}), i.e. partition f_3 (equation S8) was set using $K_{f3} = 0.11\ mM^{-1}$ to be consistent with the exchange detected by isotope entrainment at 1 mM Ni. It should be noted that higher Ni concentrations accelerate recycling (1-f_3) see Table S1, and this is reflected in the fitting values for [hv], Table S2, entries 16-23.

e). Competing Ar-solvent coupling (k_{solv}) versus commitment (k_{Si}), i.e. partition f_4, was guided by systematic variations in silane; section S3.7.5. The empirical partitioning constant ($K_{f4} = 2.05 \times 10^3$, dimensionless, based on $[(TMS)_3SiH]_0$, Figure S23) differs from the fitting value of $K_{f4} = 2.74 \times 10^3$ (eq. S10) because the former is determined by the cumulative selectivity. The fitting value reflects the instantaneous partitioning which varies with $[(TMS)_3SiH]_t$.

f). Competing Ar-H generation (k_H) versus reaction with alkyl bromide (k_{RBr}), i.e. partition f_5, was guided by systematic variations in alkyl bromide **1a**; section S 3.7.4. The empirical partitioning constant ($K_{f5} = 0.41\ mM^{-1}$, based on $[\mathbf{1a}]_0$, Figure S22) differs from the fitting value of $K_{f5} = 0.39\ mM^{-1}$ (eq. S12) because the former is determined by the cumulative selectivity. The fitting value reflects the instantaneous partitioning which varies with $[\mathbf{1a}]_t$.

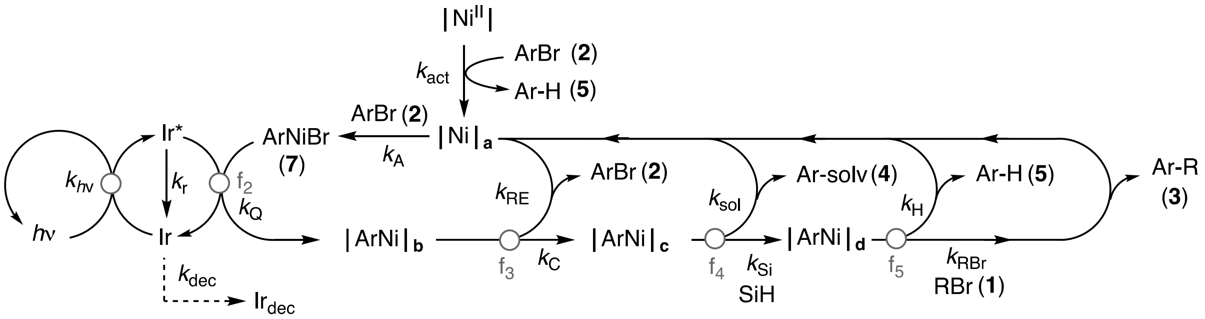

Figure S30. Model employed for kinetics simulations *at low concentrations*; see Sections S5.1, S5.2 and S5.3 for full discussion. Subscripts a-d are labels not speciation. Empirically, the unproductive partition (1-f_2) is catalyzed by Ir, and analogously (1-f_3) by Ni. See Table S2 entries 16-28, Figure S7, and sections S.3.7.1 and S.3.7.2.

$$\frac{d[\mathrm{h\nu}]}{dt} = 0; \text{ and } [\mathrm{Ir}] >> [\mathrm{Ir}^*] \qquad \text{eq S1}$$

$$\frac{d[\mathrm{Ir}^*]}{dt} \approx k_{h\nu}[\mathrm{h\nu}][\mathrm{Ir}] - [\mathrm{Ir}^*](k_Q[\mathrm{ArNi^{II}Br}] + k_r) \qquad \text{eq S2}$$

$$\frac{d[\mathrm{Ir}]}{dt} \approx -k_{dec}[\mathrm{Ir}]; \text{ and } [\mathrm{Ir}] \approx [\mathrm{Ir}]_0 - [\mathrm{Ir}_{dec}] \qquad \text{eq S3}$$

$$f_2 \approx \left[\frac{1}{1+\frac{1}{K_{f2}[\mathrm{ArNiBr}]}}\right]; \;\; K_{f2} = \frac{k_Q}{k_r} \qquad \text{eq S4}$$

$$\frac{d[\mathrm{Ni}_a]}{dt} \approx (k_C + k_{RE})[\mathrm{ArNi}_b] + k_{act}[\mathrm{Ni^{II}}][\mathrm{ArBr}] - k_A[\mathrm{Ni}_a][\mathrm{ArBr}] \qquad \text{eq S5}$$

$$\frac{d[\mathrm{ArNi^{II}Br}]}{dt} \approx k_A[\mathrm{Ni}_a][\mathrm{ArBr}] - k_Q[\mathrm{Ir}^*][\mathrm{ArNi^{II}Br}] \qquad \text{eq S6}$$

$$\frac{d[\mathrm{ArNi}_b]}{dt} \approx k_Q[\mathrm{Ir}^*][\mathrm{ArNi^{II}Br}] - (k_C + k_{RE})[\mathrm{ArNi}_b] \qquad \text{eq S7}$$

$$f_3 \approx \left[\frac{1}{1+\frac{1}{K_{f3}}}\right]; \text{ where } K_{f3} = \frac{k_C}{k_{RE}} \qquad \text{eq S8}$$

$$\frac{d[\mathrm{ArNi}_c]}{dt} \approx k_C[\mathrm{ArNi}_b] - k_{sol}[\mathrm{DME}][\mathrm{ArNi}_c] - k_{Si}[\mathrm{SiH}][\mathrm{ArNi}_c] \qquad \text{eq S9}$$

$$f_4 \approx \left[\frac{1}{1+\frac{[\mathrm{DME}]}{K_{f4}[\mathrm{SiH}]}}\right]; \; K_{f4} = \frac{k_{Si}}{k_{sol}}; \; [\mathrm{DME}] = 9.63\,M \qquad \text{eq S10}$$

$$\frac{d[\mathrm{ArNi}_d]}{dt} \approx k_{Si}[\mathrm{SiH}][\mathrm{ArNi}_c] - k_H[\mathrm{ArNi}_d] - k_{RBr}[\mathrm{RBr}][\mathrm{ArNi}_d] \qquad \text{eq S11}$$

$$f_5 \approx \left[\frac{1}{1+\frac{1}{K_{f5}[\mathrm{RBr}]}}\right]; \text{ where } K_{f5} = \frac{k_{RBr}}{k_H} \qquad \text{eq S12}$$

$$\frac{d[\mathrm{Ar{-}R}]}{dt} \approx k_{h\nu}[\mathrm{Ir}][\mathrm{h\nu}] \cdot f_2 \cdot f_3 \cdot f_4 \cdot f_5 \qquad \text{eq S13}$$

$$\frac{d[\mathrm{Ar{-}Sol}]}{dt} \approx k_{h\nu}[\mathrm{Ir}][\mathrm{h\nu}] \cdot f_2 \cdot f_3 \cdot (1 - f_4) \qquad \text{eq S14}$$

$$\frac{d[\mathrm{Ar{-}H}]}{dt} \approx k_{h\nu}[\mathrm{Ir}][\mathrm{h\nu}] \cdot f_2 \cdot f_3 \cdot f_4 \cdot (1 - f_5) \qquad \text{eq S15}$$

5.2 Optimisation of the Final Minimal Model (Figure S30)

The temporal concentration data of **2**, **3**, **4**, **5** and **7** obtained by ^{19}F in situ LED NMR analysis was found to be sufficiently reproducible for kinetic simulation, see for example the triplicate repeat shown in Figure S31

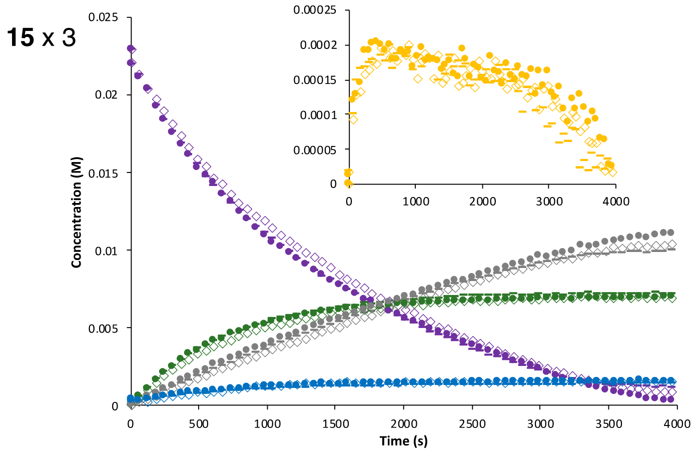

Figure S31. Reproducibility of data - three superimposed runs under the conditions of entry 15 (Table S2).

In the first stage, the model was fitted to a subset of the data (Table S2, entries 1-5). Constants k_{act}, k_{dec}, k_A, k_Q, and ratios (k_Q/k_r), (k_Q/k_{rlr}), (k_{Si}/k_{sol}), (k_{RBr}/k_H), were optimised in parallel, using a proprietary algorithm to minimise the error between experimental and predicted temporal concentrations of **2**, **3**, **4**, **5** and **7** over the entire reaction evolution, via a large series of small time-increments. The silane and alkyl bromide concentrations were allowed freedom within specified ranges that included the intended variations.

In the second stage, the model was tested on the remaining data sets (Table S2, entries 6-35) by allowing freedom in k_{act}, k_{dec}, and initial nickel, silane, alkyl bromide, and $h\nu$ concentrations within specified ranges that included intended systematic variations.

The fitted data are presented graphically for each of the 35 datasets in Figure S32, Section S5.2.2.

In this, and all models explored, numerous simplifications were made: many steps are telescoped steps and have notional irreversibility. The individual steps and associated rate constants should not be interpreted in isolation. The model (Fig S30) is predominantly 'chemically agnostic' and is presented as a phenomenological kinetic framework for reactions at low concentrations of catalyst and reactants, with short pathlength light, and for future evaluation and evolution.

5.2.1 Datasets, initial conditions and fitted variables.

entry	$[ArBr]_0$ (mM)	$[AlkBr]_0$ (mM)	$[Ni]_{tot}$ (mM)	$[Ir]_{tot}$ (mM)	$[(TMS)_3SiH]_0$ (mM)	$[ArH]_0$ (mM)	$k_{hv}[hv]$ (s^{-1})	k_{act} ($\mu M^{-1}s^{-1}$)	$k_{dec.}$ ($10^{-3}s^{-1}$)
1	10.53	**2.28**	0.25	0.49	9.39	0.33	1.56	1.20	0.70
2	10.18	**4.81**	0.25	0.45	9.58	0.29	1.56	1.30	0.68
3	10.31	**10.21**	0.25	0.44	10.15	0.28	1.56	1.50	0.55
4	10.34	**17.25**	0.25	0.48	10.34	0.25	1.56	1.80	0.71
5	10.42	**19.54**	0.25	0.45	10.72	0.25	1.56	1.50	0.49
6	11.13	12.75	0.16	0.60	**0.67**	0.19	1.56	2.00	0.77
7	11.02	12.75	0.16	0.62	**3.51**	0.19	1.56	1.30	0.64
8	10.92	12.75	0.16	0.60	**5.16**	0.22	1.56	1.30	0.57
9	11.03	12.75	0.16	0.61	**11.21**	0.23	1.56	1.30	0.38
10	11.48	10.40	0.16	0.62	**20.52**	0.23	1.56	1.30	0.33
11*	**2.48**	12.00	0.25	0.48	12.20	0.00	1.56	17.70	1.90
12	**5.48**	14.61	0.25	0.52	12.19	0.21	1.56	1.10	0.31
13	**10.82**	14.27	0.25	0.53	11.51	0.21	1.56	1.40	0.75
14	**16.00**	12.00	0.25	0.54	10.78	0.32	1.56	1.20	0.38
15	**20.59**	12.00	0.25	0.49	10.88	0.52	1.56	0.80	0.55
16	7.57	12.01	**0.13**	0.34	11.53	0.27	2.15	1.30	0.60
17	7.81	13.00	**0.25**	0.34	11.92	0.15	2.13	1.30	1.10
18	7.72	13.00	**0.50**	0.37	12.07	0.44	1.78	0.36	1.20
19	7.68	12.00	**0.75**	0.33	12.04	0.20	1.28	0.31	1.00
20	7.52	13.00	**1.00**	0.35	10.48	0.32	0.48	0.28	0.15
21	7.81	12.63	**1.50**	0.66	10.46	0.37	0.18	0.23	0.18
22	7.67	11.50	**2.00**	0.33	9.15	0.08	0.28	0.20	0.21
23	7.64	11.50	**2.50**	0.26	9.00	0.23	0.25	0.25	0.69
24	10.35	8.00	0.18	**0.11**	9.92	0.19	6.02	1.30	0.61
25	10.29	11.00	0.18	**0.26**	10.96	0.26	2.64	1.30	0.52
26	10.32	10.26	0.18	**0.47**	11.08	0.19	2.16	1.30	0.58
27	10.15	8.00	0.18	**0.97**	11.46	0.20	1.04	1.30	0.65
28	10.78	8.00	0.18	**1.47**	11.29	0.22	0.72	1.30	0.85
29	10.77	15.00	0.14	0.53	**0.00**	0.07	1.56	1.30	0.65
30	11.68	15.10	0.77	0.58	11.20	0.00	**0.11**	0.17	0.13
31	11.69	15.10	0.77	0.57	11.20	0.13	**0.22**	0.20	0.20
32	11.64	15.10	0.77	0.62	11.20	0.21	**0.33**	0.28	0.40
33	11.60	15.10	0.77	0.64	11.20	0.25	**0.45**	0.37	0.63
34	11.51	15.10	0.77	0.58	11.20	0.21	**0.50**	0.31	0.46
35	11.39	15.10	0.77	0.53	11.20	0.11	**0.56**	0.53	0.60

* behaved as an outlier to multiple trends established across the data-sets.

Table S2. Final parameters used for fitting. **Bold** numbers are initial parameters that were systematically varied between and within datasets, and the fitting variables used to obtain best fit of the model. Blue numbers are fitting parameters for 'hv' (the proxy for incident light at iridium), Ni-catalyst activation (evident from entries 30-35 to require light) and Ir-catalyst decomposition or deactivation. Grey numbers are concentration terms that were allowed flexibility within experimental error.

5.2.2. Individual fits of datasets, numbering as in Table S2.

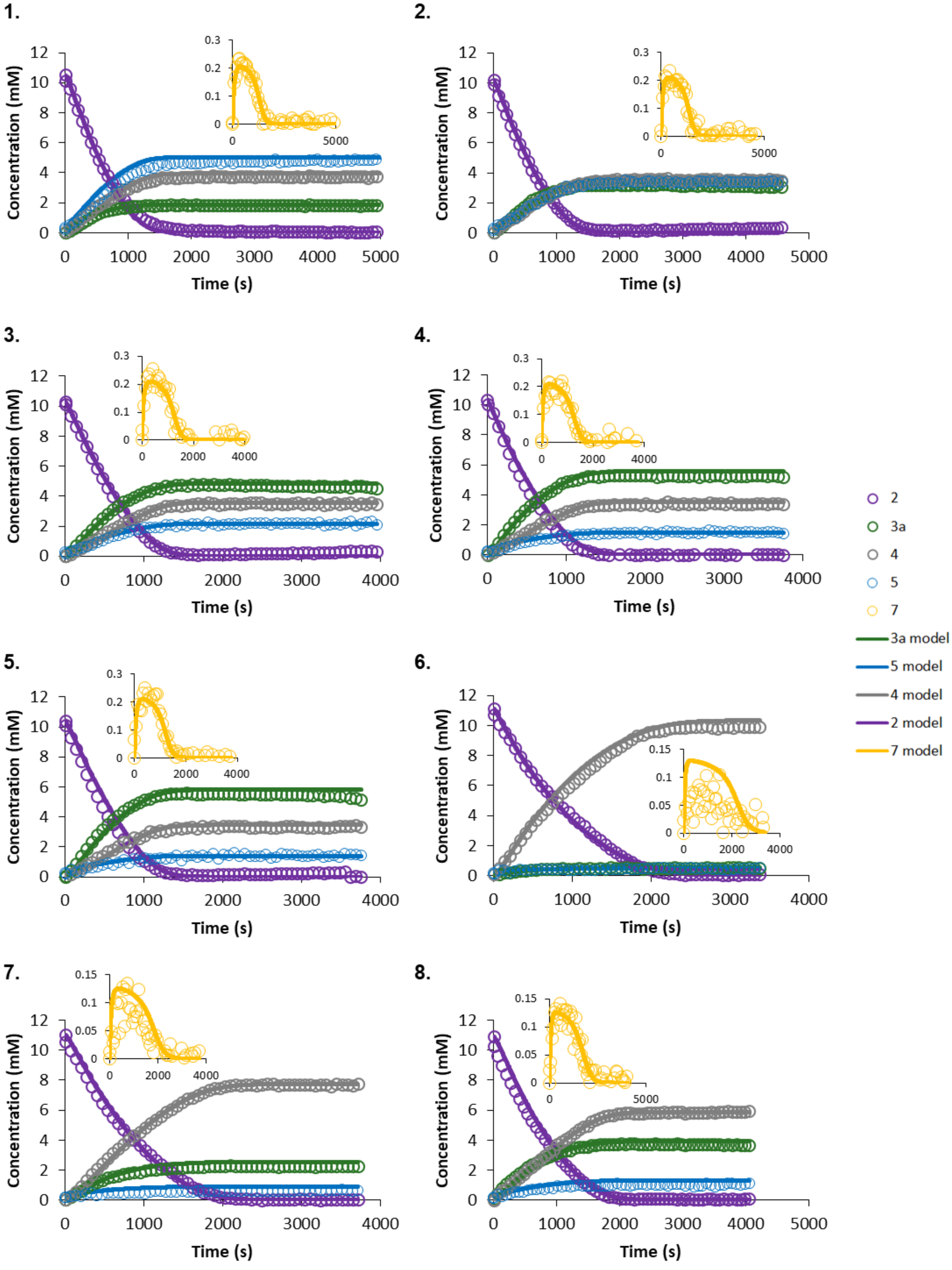

cont.

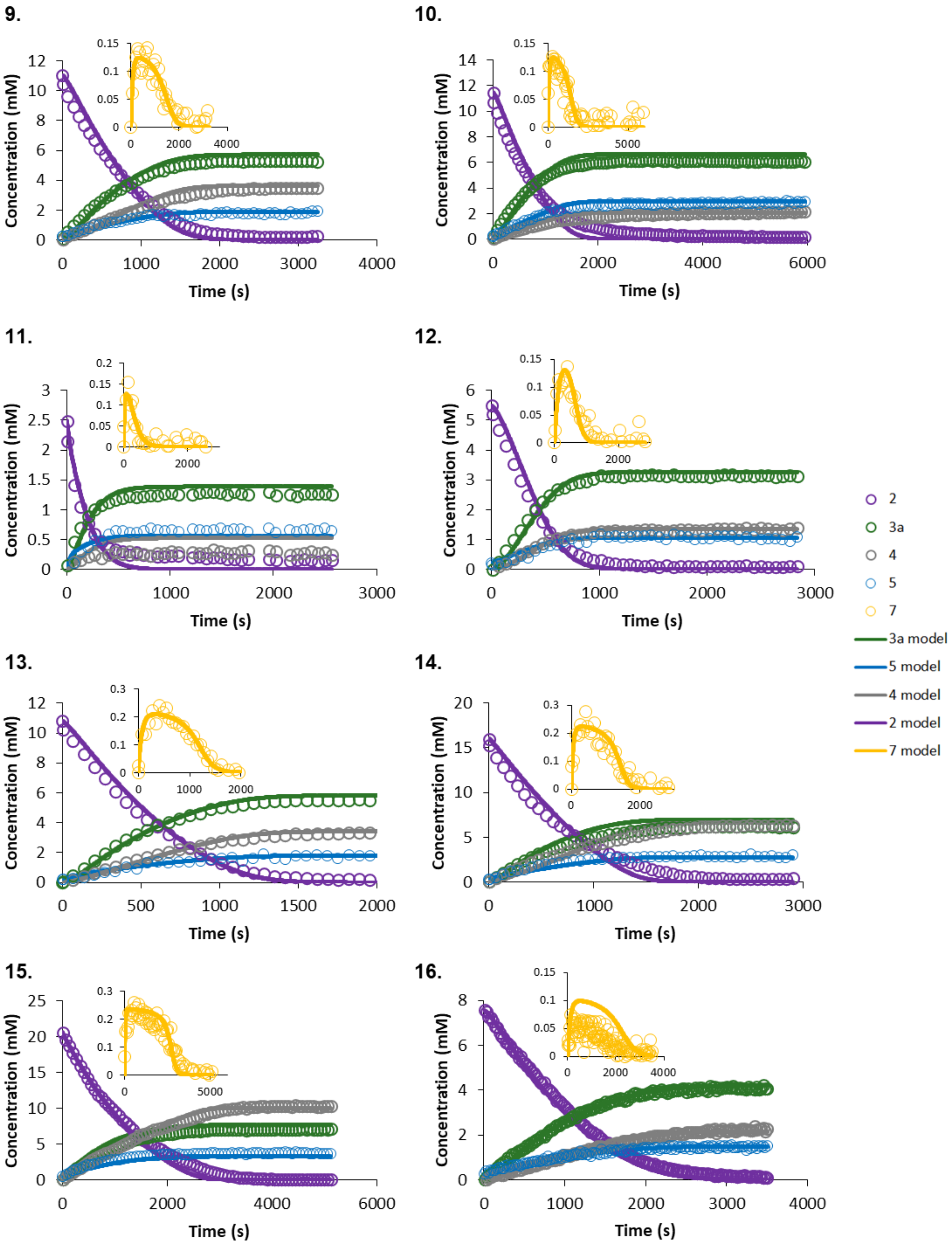
9.
10.
11.
12.
13.
14.
15.
16.
Concentration (mM)
Time (s)
2
3a
4
5
7
3a model
5 model
4 model
2 model
7 model

cont.

17.

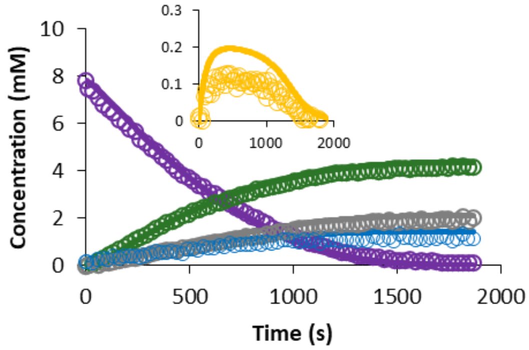

18.

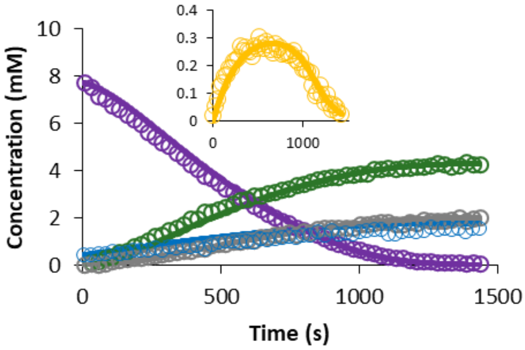

19.

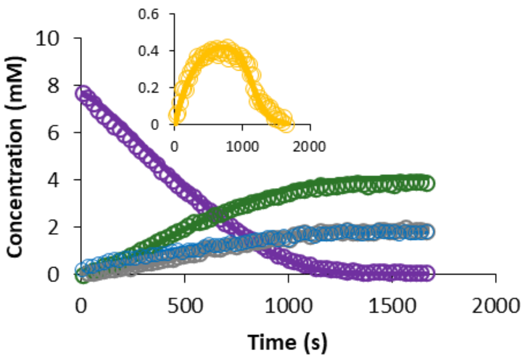

20.

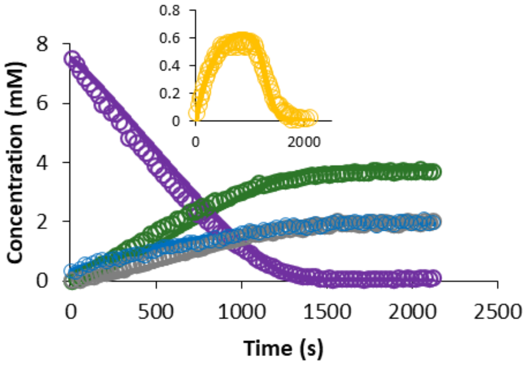

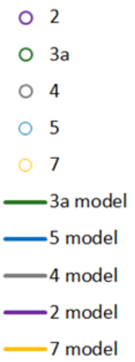

21.

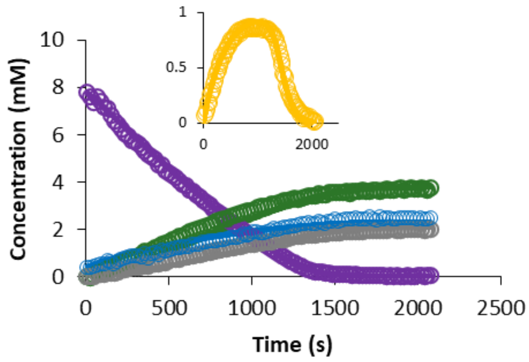

22.

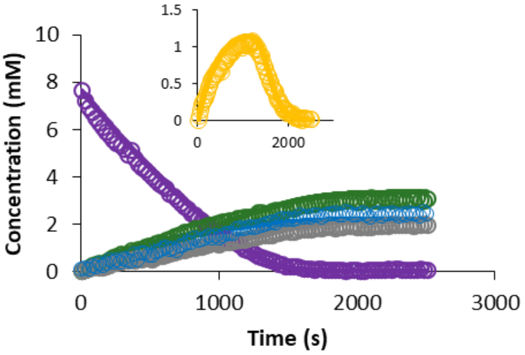

23.

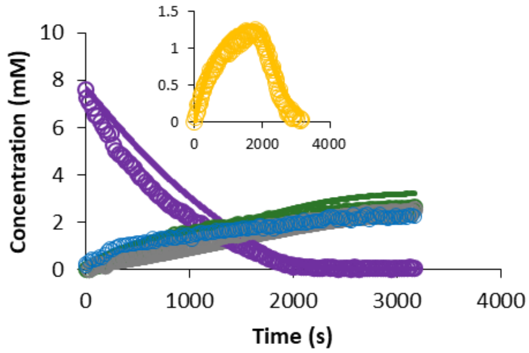

24.

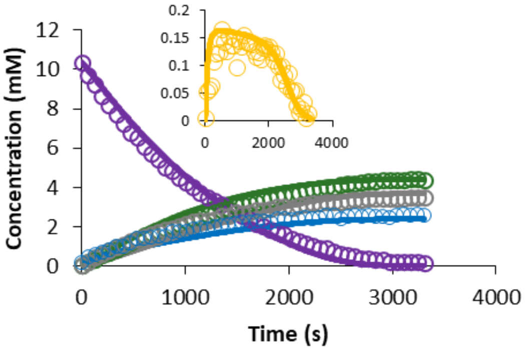

cont.

25.

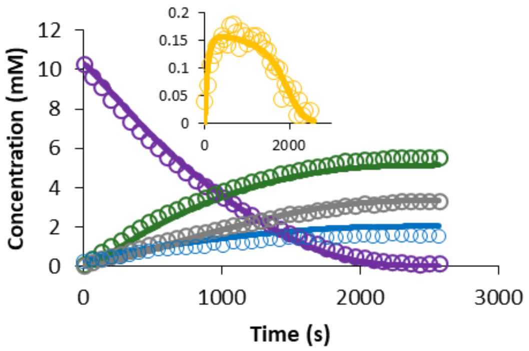

26.

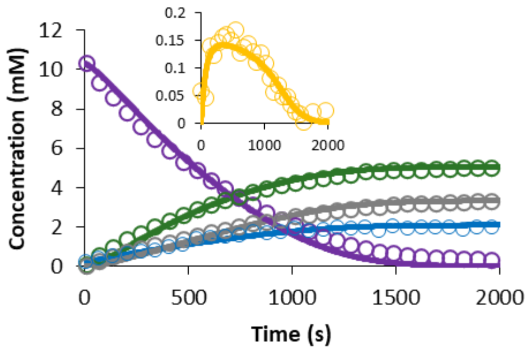

27.

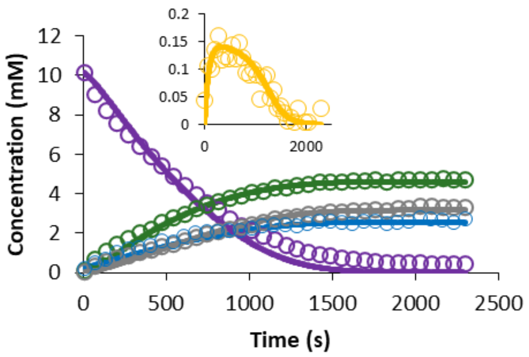

28.

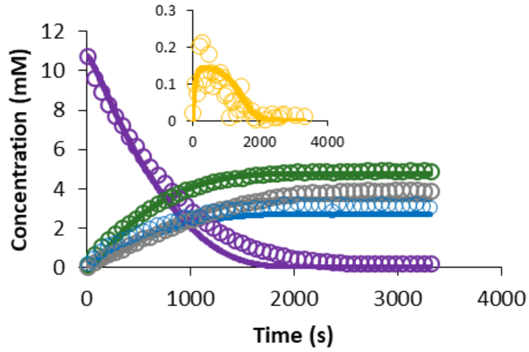

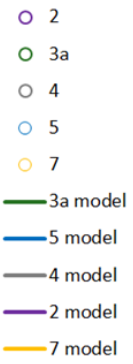

29.

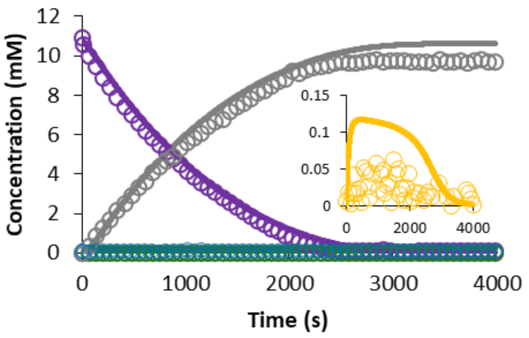

30.

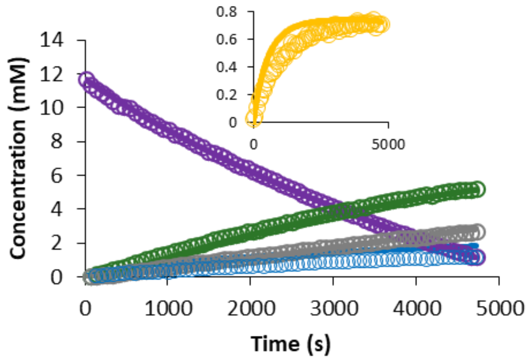

31.

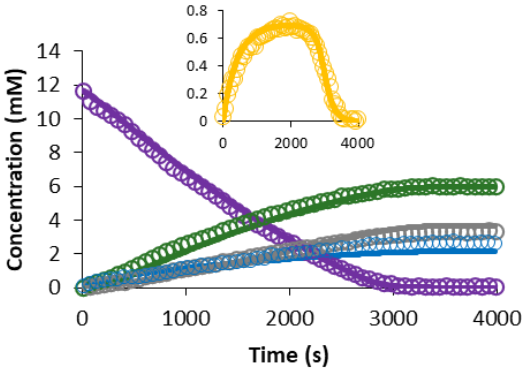

32.

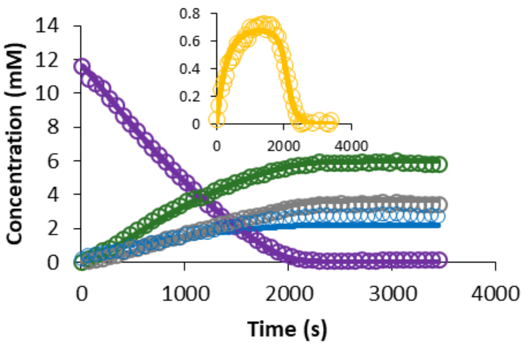

cont.

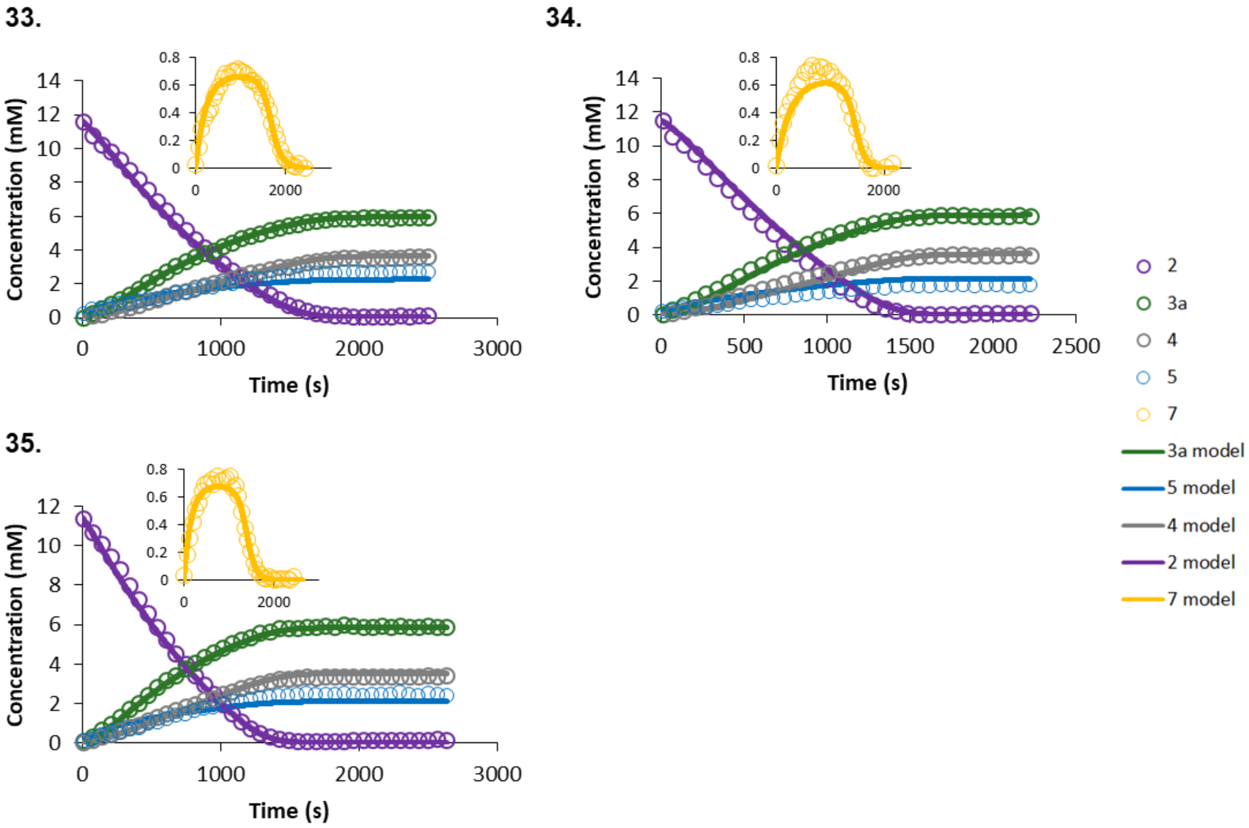

Figure S32. Experimental datasets (conditions as indicated in entries 1 to 35 in Table S2) together with kinetic simulation (solid lines) according to model shown in Figure S30.

5.3 General considerations of the Impact of Beer-Lambert Law Absorption of [Ir] and [Ni], and the effect of concentration and pathlength on the reaction rate

From the data presented in Sections S3.7 and S5.2 above, it is evident that analysis of the Ni/Ir photo-catalyzed coupling of ArBr with RBr can be simplified by considering it to comprise two discrete sections: one determining the rate of consumption of the ArBr (**2**), the other controlling the selectivity of partitioning of committed ArBr (**2**) into Ar-Solv (**4**), Ar-H (**4**) and Ar-R (**3**). In the section below, the rate of consumption of ArBr (**2**) is treated as being governed in terms of simple Beer-Lambert and steady-state rate behaviour by the concentrations of ArNiBr (**7**), the Ir photo-catalyst (**6**) and the light intensity, I_{in}. The quantum efficiency may be greater than that predicted using the light intensity value determined by actinometry if there are short chain processes. Empirically, it is found that rate attenuation occurs at raised concentrations of Ir and Ni, presumably due to (unidentified) competing quenching processes. in for example the ArBr recycling (k_{RE}) step; see Table S1 and $k_{h\nu}$[hν] in Table S2 entries 16-28.

The short pathlength (l = 0.44 mm) in the LED-NMR set-up (see Section S1), the relatively low concentrations of [Ir] and [Ni] employed in the study, and their relatively low extinction coefficients (see Section S7) result in approximately constant light intensity through the reaction volume for the majority of the conditions analysed; see later discussion in this Section (S5.3). Below we expand this to consider a more general analysis of the rate of consumption of ArBr (equation S1) and derivation of the terms f_1, f_2, and f_3 (as used in equations 1 to 4 in the main text).

The turnover rate of the productive cycle involves direct or indirect interaction between the excited photocatalyst and the resting state intermediate, **7**, and can be described by the approximate rate law given in equation S16 (see also equations S1 to S15).

$$\frac{-d[ArBr]}{dt} \approx I_{in} \cdot f_1 \cdot f_2 \cdot f_3 \qquad \text{(eq S16)}$$

In equation S16, term f_1 describes the net absorption of the incident light, I_{in}, and its fractionation into productive photoactivation of the iridium. Term f_2 describes the productive transfer of catalytic impetus from photoactivated Ir* to ArNiBr complex **7**, versus all other competing decay pathways. Term f_3 describes the productive fractionation of the activated Ar-Ni species into product-generating turnover, relative to degenerate turnover that returns ArBr to the substrate pool. The analysis below is based on the approximation that the concentrations of ArNiBr complex **7** and the ground-state iridium complex **6** are in large excess over a relatively short-lived photoactivated iridium complex, Ir*, so that a steady-state of Ni-activation is achieved. This is consistent with most observations made on the major phase of the reaction evolutions, see Sections S3.7.1 and S3.7.3, where at the steady state [**7**] ≈ $[Ni]_{tot}$ = $[Ni]_0$, without dependency on [ArBr]. These approximations break down in the final phases of reaction through depletion of the aryl bromide concentration and photocatalyst degradation; at high Ni pre-catalyst loadings (see e.g. 20 and 25 mol%, Section S3.7.1) where activation is incomplete and a steady-state of [**7**] is not reached, where high ArNi(L)Br concentrations decrease f_3 (see Table S1), and at high iridium concentrations where self-quenching or analogous deactivating effects, e.g. disproportionation, can occur.

Under the approximations outlined above, the rate of photoexcitation of the iridium (I_{Ir}) is treated as being equal to the flux of light absorbed by the ground-state photocatalyst, and at steady state this equals the *total* rate of decay (k_d) of Ir* back to the ground-state, equation S17.

$$\frac{d[Ir*]}{dt} \approx 0 \approx I_{Ir} - k_d\,[Ir^*] \qquad \text{(eq. S17)}$$

In the case where there are two dominant competing light absorbers in solution (in this case, iridium photocatalyst **6**, and ArNiBr complex **7**) the *total* light absorption, I_A, over pathlength l must be fractionated between **6** and **7** by weighting according to their concentrations, *c*, and extinction coefficients, ε. Rearrangement of the Beer-Lambert law, equation S18, gives the light intensity that is absorbed by the solution I_A, equation S19.

$$A = \log_{10}\frac{I_{in}}{I_T} = l\,(\varepsilon_{Ir}c_{Ir} + \varepsilon_{Ni}c_{Ni}) \qquad \text{(eq. S18)}$$

$$I_A = I_{in}\{1 - 10^{-(A_{Ir}+A_{Ni})}\} \qquad \text{(eq. S19)}$$

The weighting function, f_1, for absorption by iridium versus total absorption by nickel and iridium is given by equation S20. This can be combined with equation S18 to give *net* rate of photoexcitation of the iridium photocatalyst, I_{Ir}, over pathlength l, equation S21.

$$f_1 = \{1 - 10^{-(A_{Ir}+A_{Ni})}\}\frac{A_{Ir}}{A_{Ir}+A_{Ni}} = \{1 - 10^{-(A_{Ir}+A_{Ni})}\}\frac{1}{1+\frac{1}{K_{f1}}}\ ;\ \text{where } K_{f1} = \frac{A_{Ir}}{A_{Ni}} \qquad \text{(eq. S20)}$$

$$I_{Ir} = I_{in}\,\frac{\{1-10^{-(A_{Ir}+A_{Ni})}\}}{1+\frac{1}{K_{f1}}} = I_{in}\cdot f_1 \qquad \text{(eq. S21)}$$

The steady state equation S17, gives the net rate of decay of Ir*, equation S22. This can be considered as comprising two competing processes: productive encounter with **7**, k_Q, versus all other non-productive relaxation processes, k_r, equation S23; note that the rate coefficient k_r may contain concentration terms, e.g. [Ir] for self-quenching. Rearrangement gives equation S24, with weighting function f_2 that reflects the fraction of photoactivated Ir* that undergoes productive transfer of catalytic impetus to ArNiBr complex **7**.

$$I_{Ir} \approx k_d\,[Ir^*] \qquad \text{(eq. S22)}$$

$$k_d\,[Ir^*] \approx k_Q[Ir^*][ArNiBr] + k_r[Ir^*] \qquad \text{(eq. S23)}$$

$$f_2 \approx \frac{k_Q\,[Ir^*][ArNiBr]}{k_Q\,[Ir^*][ArNiBr]+k_r\,[Ir^*]} = \frac{1}{1+\frac{1}{K_{f2}\,[ArNiBr]}}; K_{f2} = \frac{k_Q}{k_r} \qquad \text{(eq. S24)}$$

Equation S25 provides the weighting function f_3 that reflects the fraction of productive encounter of Ir* with **7** that leads to arylbromide commitment and thus to ArNiBr derived products, **3**, **4** and **5**.

$$f_3 \approx \frac{k_C\cdot f2\,k_d[Ir^*]}{k_C\cdot f2\,k_d[Ir^*] + k_{RE}\cdot f2\,k_d[Ir^*]} = \frac{1}{1+\frac{1}{K_{f3}}}\ ;\ K_{f3} = \frac{k_C}{k_{RE}} \qquad \text{(eq. S25)}$$

Combining equations S21, S24 and S25 gives a steady state approximation, equation S26, for the rate of generation of ArBr derived products as a function of light intensity, iridium and nickel concentration, and pathlength. It is important to note that this reflects the net rate across the reaction volume pathlength, not the instantaneous rate at a given pathlength.

$$\frac{-d[ArBr]}{dt} \approx I_{in} \frac{\left[1-10^{-(A_{Ir}+A_{Ni})}\right]}{\left[1+\frac{1}{K_{f1}}\right]\left[1+\frac{1}{K_{f2}\,[ArNiBr]}\right]\left[1+\frac{1}{K_{f3}}\right]} = I_{in} \cdot f_1 \cdot f_2 \cdot f_3 \quad \text{(eq. S26)}$$

This model can also be modified to be more accurate to the specific apparatus used in this study in which an internal cylindrical source radiates light outwards into a cylindrical reservoir of solution. The reaction volume can be treated as a series concentric cylinders, which when 'sliced' horizontally each contain a ring of area, A_{slice}.

$$A_{slice} = \pi {r_n}^2 - \pi {r_{n-1}}^2 \quad \text{(eq. S27)}$$

The average light intensity (I_{av}) experienced by the overall solution can be expressed as:

$$I_{av} = \sum I_{t_{previous\ slice}} \cdot \frac{A_{slice}}{A_{total}} \quad \text{(eq. S28)}$$

At the scale of the LED-NMR device it was found that this weighting had a very minimal effect on the predicted averaged light intensity, and thus rates of reaction, compared to a simple linear approximation. However, at the longer pathlengths employed in synthesis, the difference between linear and radial analysis becomes substantial. Employing the experimentally determined extinction coefficients (see Section S7) for **6** and **7**, and application of the Beer Lambert law across the 0.044 cm pathlength of the LED-NMR cell, at the concentrations of Ir and Ni employed for the typical conditions (Table S2, entries 1-15; 29-35) indicates that the average light across the pathlength varies by about ±10%. Even at the highest Ni loadings (see Section S3.7.1) the light intensity only varies by about ±25% across the reaction volume; Figure S33. In synthetic applications of photocatalytic reactions, the solution is usually being agitated during irradiation. In contrast, the reaction volume in the LED-NMR cell is nominally stagnant, with mass transfer occurring by convection and diffusion. However, the short pathlength and homogeneous nature of the initial reaction volume means that negligible chemical gradients are established as the reactions proceed.

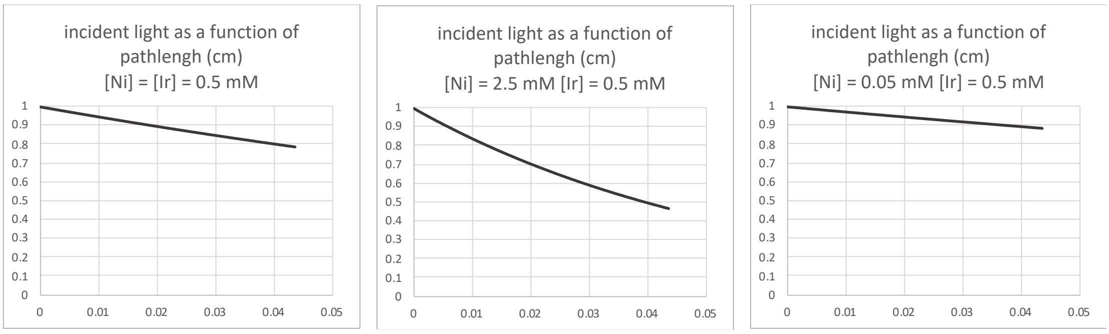

Figure S33. Calculated attenuation of incident light (y-axis, arbitrary units) as a function of pathlength (x-axis, cm) at three concentrations of Ir and Ni representative of typical and limiting conditions employed in the LED-NMR cell (pathlength 0.044 cm), using experimentally determined extinction coefficients.

Equation S26 can in principle be employed in a generic sense to explore the impact of light intensity, iridium and nickel concentration, and pathlength. However, additional Ni and Ir-mediated quenching results in the rate/concentration profiles similar to those reported for several different photocatalytic reactions by Wittenberger and coworkers[S17] see Section S3.7.1, where experimentally the rate of reaction reaches saturation (rate maximum) at a lower [Ir] concentration than predicted by eq. S21.

6. Potential Mechanisms

In the discussion above, and in the main text, we have been deliberately 'agnostic' about many aspects of the chemical / catalyst speciation. The kinetic behaviour that has been elucidated (Section 3.7, and Figure S18) and simulated using the model (Figures S30) discussed in Section S5 is phenomenologically consistent with a wide range of possible mechanisms. In this section we provide additional mechanistic and kinetic considerations to supplement the main text.

In the main text we considered three generic examples (**I**, **II**, **III**) that contain some of the more commonly encountered components / sequences for such couplings.[S18] We noted that the general kinetic behaviour for the specific case reported herein (**1** + **2**), can be readily fit using Figure S30 as a generic model for mechanisms **I** and **II** (Figure S34) under most conditions. In contrast, very specific conditions and constraints are required to adapt models based on **III**, to obtain any consistent behavior. Indeed, to the best of our efforts we were unable to find a general fit for this (or mechanism **IV** which was excluded from the main text as it does not feature an ArNi^{II}(L)Br intermediate), with significant deviations between the predicted and general kinetic behaviour across the 35 datasets that were explored (Table S2, Figure S32).

6.1 Processes facilitating a general fit of the model

The two generic mechanisms (**I**, and **II**) that the model (Figures S18 and S30, Section S5) can be readily reconciled with in terms of phenomenological kinetics were shown in Figure 7a in the main text and are reproduced below in Figure S34. With appropriate configuration, both mechanisms result in turnover-rate limiting dependencies on light, Ir, Ni, and ArBr, and the selectivity dependent on $(TMS)_3SiH$ and RBr concentrations.

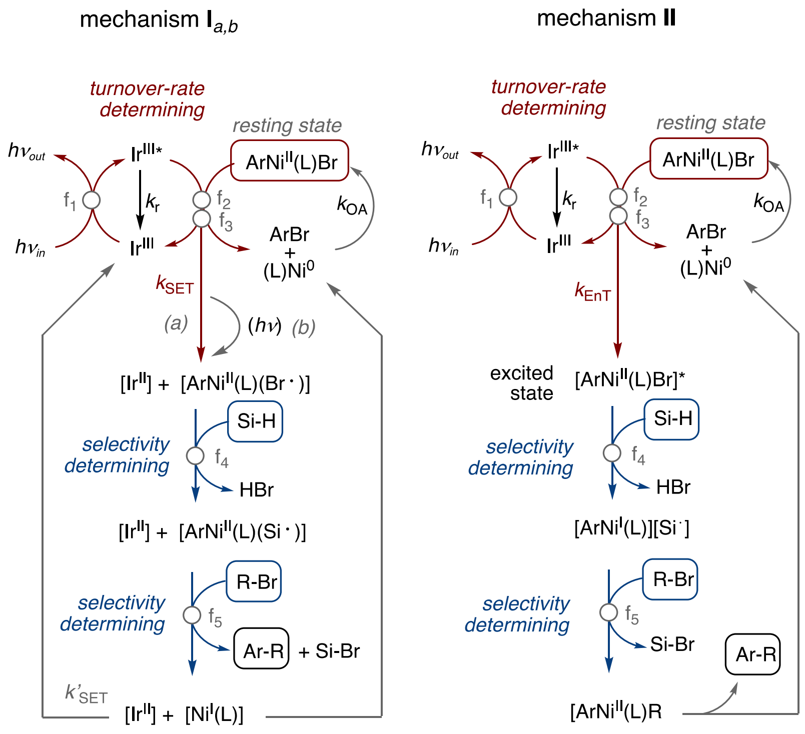

Figure S34. Generic mechanisms **I** and **II** with which, under specific conditions, the model (Figures S18 and S30; plus discussion in section S5) can be phenomenologically consistent with in terms of overall kinetic behaviour.

As discussed in the main text, the key distinction between the two mechanisms is that mechanism **I** involves electron transfer (k_{SET}) between an ArNiBr intermediate and the excited state photocatalyst (Ir*), while mechanism **II** involves energy transfer (k_{EnT}) between the same species. This results in different Ni speciation and oxidation states (Ni and Ir) and thus mechanisms for (non-rate limiting) selectivity. Prior investigations on similar systems have been unable to definitively distinguish these processes (energy transfer or electron transfer),[S19] although we note that in the absence of any further information, mechanism **II** might be favored over mechanism **I** as it does not require a non-rate limiting interaction of two highly reactive species in low concentration ($k'_{SET}[Ir^{II}][Ni^{I}]$). We also note that interaction between the photocatalyst and the NiArBr species to generate a common intermediate (that can either decay into Ni(0) and aryl bromide or further react productively), is kinetically indistinguishable from the case where two separate interactions between the photocatalyst and the NiArBr intermediate result in two or more intermediate species, with one or more of which decaying into Ni(0) and aryl bromide, while one or more react productively. Furthermore, photocatalysed steps that are outside of the turnover-rate limiting events and that do not affect selectivity (e.g. mechanism $\mathbf{I}_b$) are kinetically indistinguishable from the data generated (Figures S18 and S30; plus discussion in section S5).

6.2 Processes for which a general fit of the model could not be found

The generic mechanism **III** was considered in the main text. Below we also include generic mechanism **IV**. Both contain a number of features by which they differ from the experimental observations reported herein (Figures S18 and S30, Section S5).

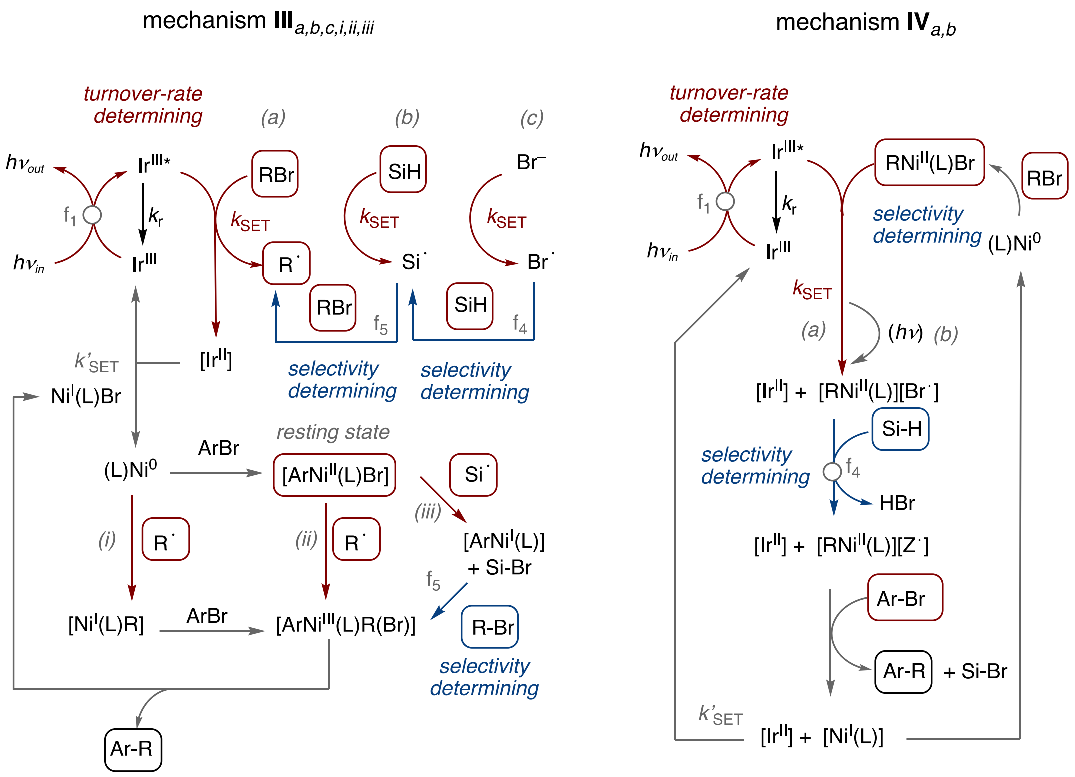

Figure S35. Generic mechanisms (**III** and **V**) with which the model (Figures S18 and S30; plus discussion in section S5) appears to be phenomenologically inconsistent in terms of specific or overall kinetic behaviour; see text for further discussion.

Mechanism **III** comprises components of some of the most commonly invoked steps in C-C bond forming dual nickel/photocatalysis. The overarching mechanism involves the generation of a carbon centred radical by means of either a direct redox exchange with the excited state photocatalyst (**III**$_a$), or an indirect process mediated by a radical generated from an additive, e.g. silane or amine (**III**$_b$), the latter process can itself be mediated by halide redox (**III**$_c$). The carbon-based radical undergoes a one electron addition to a nickel centre, either to Ni(0) (**III**$_i$) followed by ArBr addition, or to ArNi(II)Br complex (**III**$_{ii}$). Alternatively, a silyl radical can add to ArNi(II)Br, (**III**$_{iii}$), followed by RBr. All three paths lead to the same Ar-Ni(III) complex that reductively eliminates the Ar-R product, co-generating Ni(I) which is reduced to Ni(0) by SET from the reduced form of the photocatalyst, thus regenerating the photocatalyst ground state. Pathways **III**$_{ii}$ and **III**$_{iii}$ feature an ArNi(II)Br complex as a resting state, which has been shown via isotope entrainment to be a productive intermediate. However, the radical generating steps in **III**$_a$ and **III**$_b$ could cause kinetic dependency on [RBr] or [$(TMS)_3SiH$] which are not detected in the current system (Figures S18 and S30, Section S5). Under limiting conditions of rate-limiting generation of Br-radical from bromide counter-anion, with rapid capture of the Br radical by SiH then RBr, pathways **III**$_{cii}$ and **III**$_{ciii}$ appear in principle to be consistent: an ArNi(II)Br complex resting state is captured in a light-intensity dependent step, with [RBr] and [$(TMS)_3SiH$] only controlling the *selectivity* (e.g. where Br-radical mediates solvent coupling of ArNi(II)Br and $(TMS)_3Si$-radical mediates generation of ArH from ArNi(II)Br). The Br-radical generation would need to be irreversible, or only be reversible at accumulated Ir^{II} concentrations, and the kinetics would be pseudo zero-order dependent on ArNi(II)Br, Ni, or ArBr. Experimentally, the rate and selectivity of the reaction is found to be independent of endogenous bromide, see section S9, both in the form of accumulating lutidinium bromide, and exogenous bromide. The SET from bromide would thus need to occur to a manner that is independent of progressive changes in counter-anion speciation to the cationic iridium photocatalyst, see section S9.

Mechanism **IV** is analogous to Mechanism **I** but involves SET to an alkyl nickel complex RNi(II)Br. While this is consistent with the data when conversion of Ni(0) to RNi(II)Br is not turnover-rate limiting, the ArBr (**2**) will act as an inhibitor by biasing the major speciation of the Ni towards the spectroscopically characterised ArNi(II)Br complex (**7**). Moreover the latter has been shown via isotope entrainment to be a productive intermediate.

7. In Situ UV-Vis Spectra and Extinction Coefficients

7.1 Photocatalyst 6

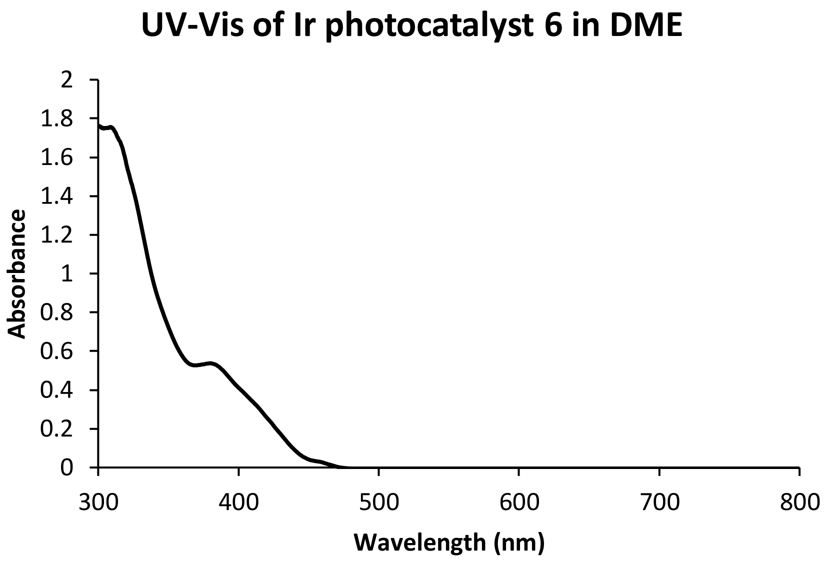

Figure S36 UV-Vis spectrum of complex **6** (0.11 mM in DME).

A solution of 0.553 M **6** in DME was prepared using standard volumetric glassware. Four further solutions were made by serial dilution from the initial solution, and the UV-Vis spectra of each solution was obtained. The absorbances at 420 nm (the wavelength used in this study) and 455 nm (a wavelength more typically used in synthetic photoredox catalysis) were plotted against concentration to calculate the extinction coefficient, ε, of the complex in DME at each wavelength.

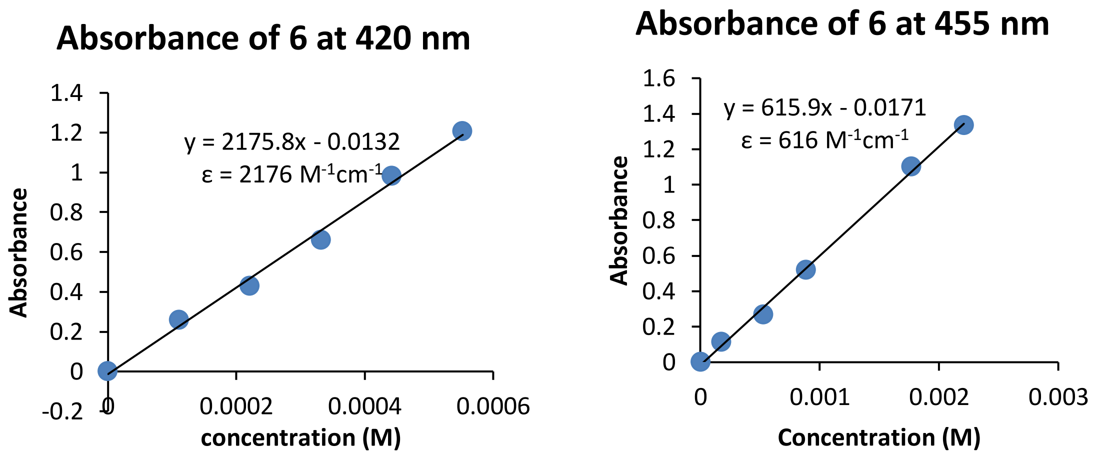

Figure S37 Analysis of the extinction coefficient of **6** at 420 and 455 nm.

7.2 Ni(II) species 7

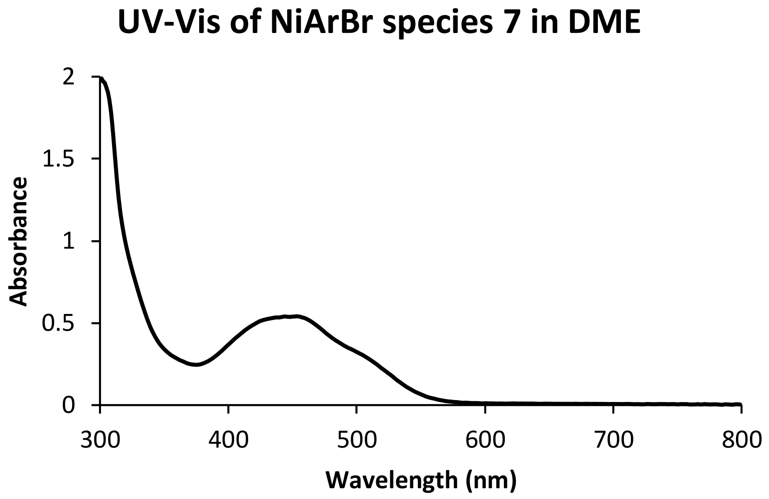

Figure S38 UV-Vis spectrum of complex **7** (0.18 mM in DME).

A solution of 0.177 M **7** in DME was prepared in a nitrogen-filled glovebox using standard volumetric glassware. A sample of this solution was placed in a quartz cuvette with a screw fitting. The cuvette was sealed with a rubber-lined screw top, removed from the glovebox, and a UV-Vis spectrum of the solution was taken. Three further solutions were made by serial dilution from the initial solution, and UV-Vis spectra of each solution was obtained similarly. The absorbances at 420 nm (the wavelength used in this study) and 455 nm (a wavelength more typically used in synthetic photoredox catalysis) were plotted against concentration to calculate the extinction coefficient, ε, of the complex in DME at each wavelength.

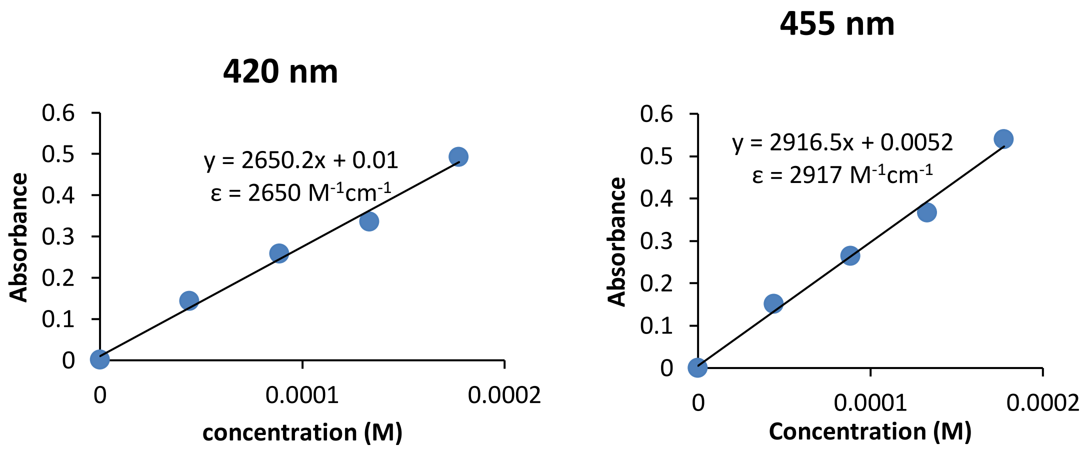

Figure S39 Analysis of the extinction coefficient of **7** at 420 and 455 nm.

7.3 Changes in UV-Vis Spectrum during the Ar-Br/RBr cross coupling.

The following experiment was conducted to assess whether small quantities of highly UV-vis (420 nm) absorbing Ni or Ir species (other than **6** and **7**) are generated during the RBr (**1**) + ArBr (**2**) coupling and attenuate the rate by competitive absorption. A reaction was assembled under the standard conditions and then sealed in a 5 mm O.D. quartz NMR tube. After periodically irradiating the sample, for durations and intensities known to cumulatively take the reaction to complete consumption of **2**, intercalated UV-Vis spectra were acquired using a pair of optical fibres with focussing lenses (approximate pathlength 4 mm) transmitting the beam through the solution in the tube (orthogonal to the vertical axis). The resulting sequential spectra are shown in Figure S40. There is no significant deviation in the series of spectra from that which would be expected based on the presence of **6** and growth of endogenous **7** at known concentrations and extinction coefficients (the initial spectrum is plotted in red). After the final period of irradiation, and full consumption of ArBr (**2**) a fine suspension of Ni black is visually detected and this results in a change in the background absorption (the final spectrum is plotted in purple). The spectrum calculated based on the expected concentrations and extinction coefficients is plotted as a dashed line. The calculated maximum absorption at 420 nm is 0.88, the observed maximum is 0.86.

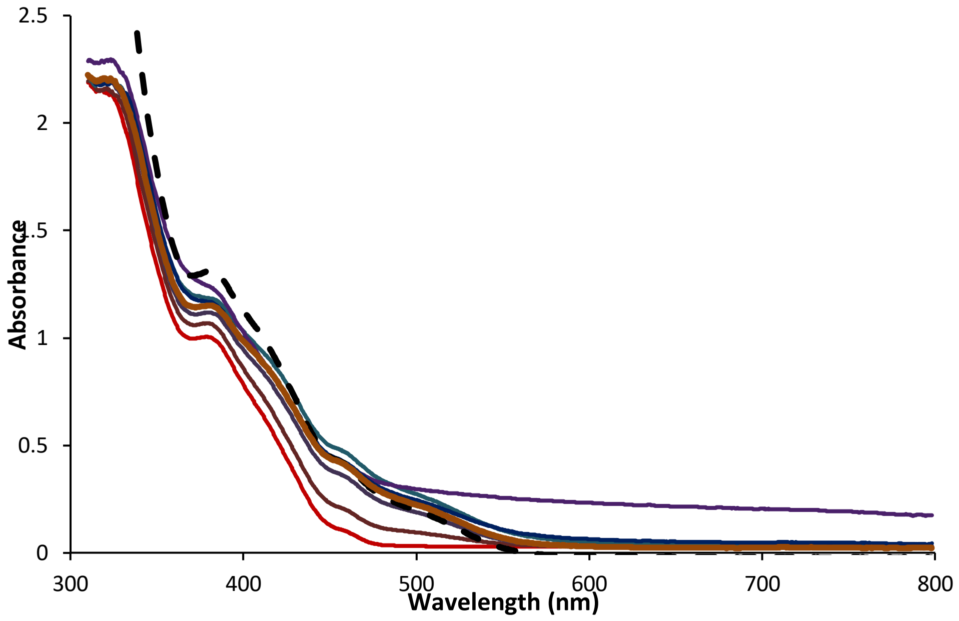

Figure S40. Stacked UV-Vis spectra of the cross-coupling reaction assembled under the standard conditions (15 mM **1b**, 10 mM **2**, 0.25 mM $NiCl_2$(DME), 0.25 mM dtbbpy, 0.5 mM **6**, 10 mM $(TMS)_3SiH$, 20 mM 2,6-lutidine) acquired between intermittent periods of continuous irradiation at 420 nm. The dashed line shows the spectrum predicted from the maximum expected concentrations of **6** and **7** using the experimentally determined extinction coefficients.

8. Behaviour of Iridium complex 6 on irradiation at 420 nm.

When a solution of photocatalyst **6** in DME (5 mM) is irradiated at 420 nm, there is progressive line broadening of the CF_3 signal in the ^{19}F NMR (376.5 MHz) spectrum, as well as a slight chemical shift change ($\Delta\delta_F$ = 0.8 Hz). These changes are also observed in the *in situ* LED-^{19}F NMR spectrum during the ArBr + RBr coupling reaction. Upon cessation of irradiation the ^{19}F NMR spectrum returned to its pre-excitation appearance with approximately (pseudo) first order kinetics of relaxation (k_{obs} = 0.028 s^{-1}), a rate that is more than six orders of magnitude slower than a simple Ir^{III*}→Ir^{III} process, which proceeds on the µs time scale.[S20] From this we conclude that at the Ir-concentrations employed for the C-C coupling reaction, there are additional redox processes occurring as a consequence of, or in parallel with, the simple Ir^{III}→Ir^{III*} excitation-relaxation phenomenon. This slower process may be related to the concentration-dependent photocatalyst self-quenching observed in the C-C coupling that results into the non-linear rate-dependency on photocatalyst at concentrations much lower than expected for simple Beer-Lambert behaviour, see Table S2, entries 24-28.

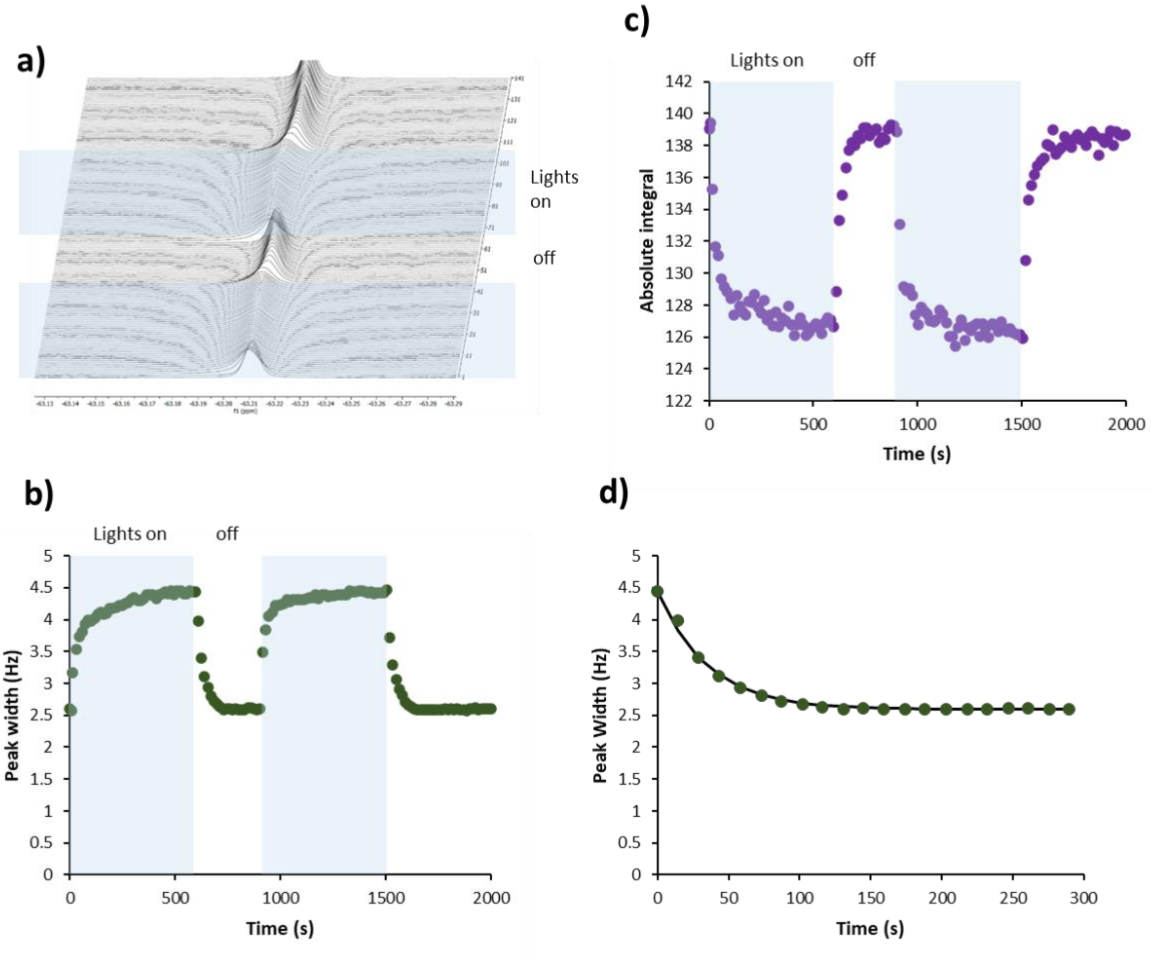

Figure S41. a) Stacked ^{19}F NMR spectra of iridium complex **6** in DME (5 mM) during intermittent periods of continuous irradiation. The data used in (b,c,d) are taken from these NMR spectra. b) A plot showing line broadening of the signal measured by peak width at half maximum height, as a function of time and light irradiation. c) A graph of absolute integral across a fixed region, as a proxy for line broadening, as a function of time and light irradiation. d) First-order fitting (solid line k = 0.028 s^{-1}; $t_{1/2}$ = 24.7 s) of the relaxation in peak width during the first lights-off section (t = 600s to 900s).

9. Effect of endogenous and exogenous bromide ion on rate and selectivity.

During the reaction a small change in chemical shift of the CF_3 signal in photocatalyst **6** is observed, along with the line-broadening discussed in Section S8. This was observed to be analogous to the effect on chemical shift of titrating photocatalyst **6** with tetrabutylammonium bromide (Figure S42) and was assigned as a change in the speciation of the associated counter-anion of the photocatalyst during the reaction.

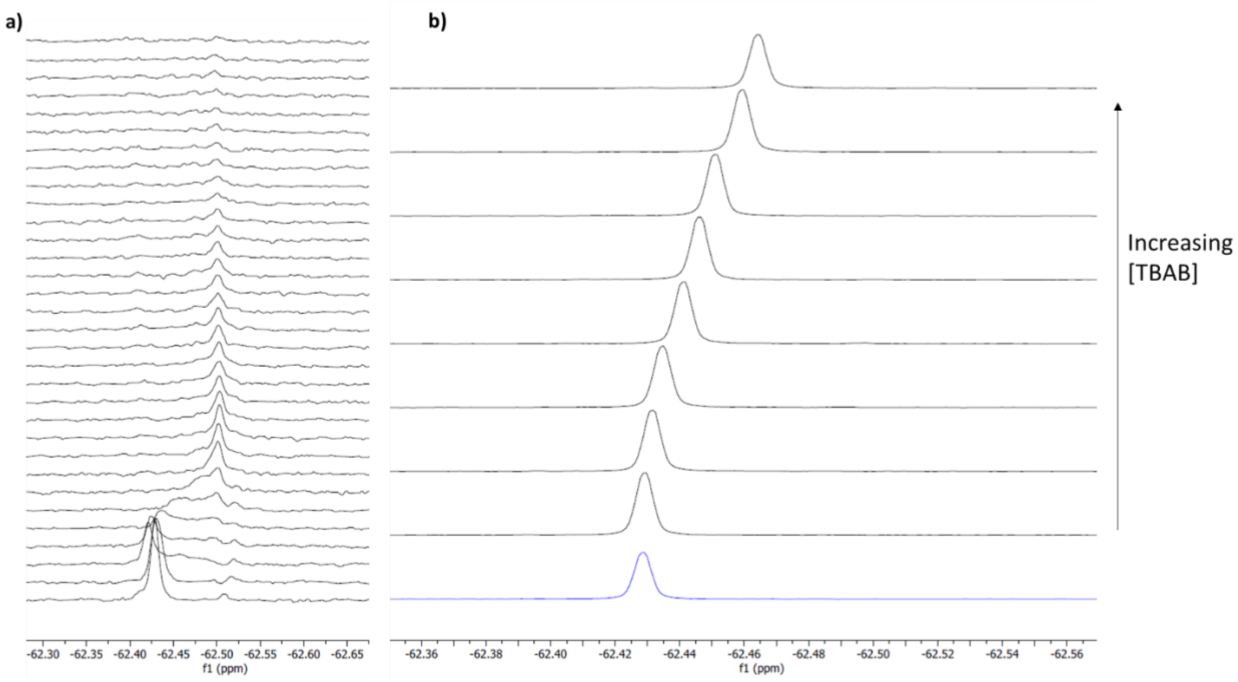

Figure S42. a) Chemical shift and intensity differences observed on the ($-CF_3$) peak of photocatalyst **6** during a typical reaction ($[Ir]_0$ = 0.5 mM). b) Titration of photocatalyst **6** (10 mM) with tetrabutylammonium bromide (0-20 mM). The bottom spectrum, in blue, has had $TBAPF_6$ added instead to ensure that the observed effect is from the presence of the bromide anion rather than the tetrabutylammonium cation.

As counterion identity is known to have an effect on photoredox catalysis[S21] a variety of experiments were conducted, with exogenous bromide introduced before, and during, the reaction. However, no significant impact on either the rate or the selectivity of the reaction was observed (Figures S43 and S44).

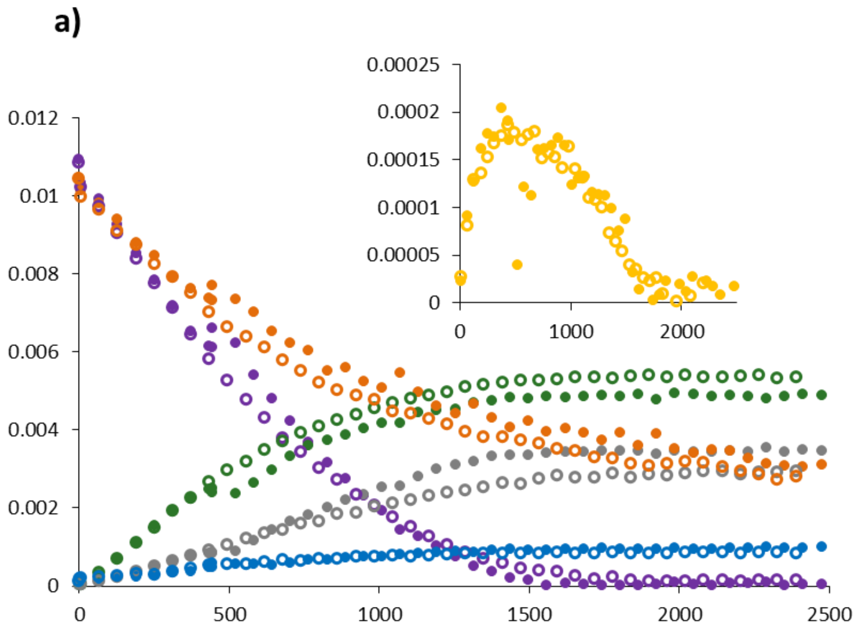

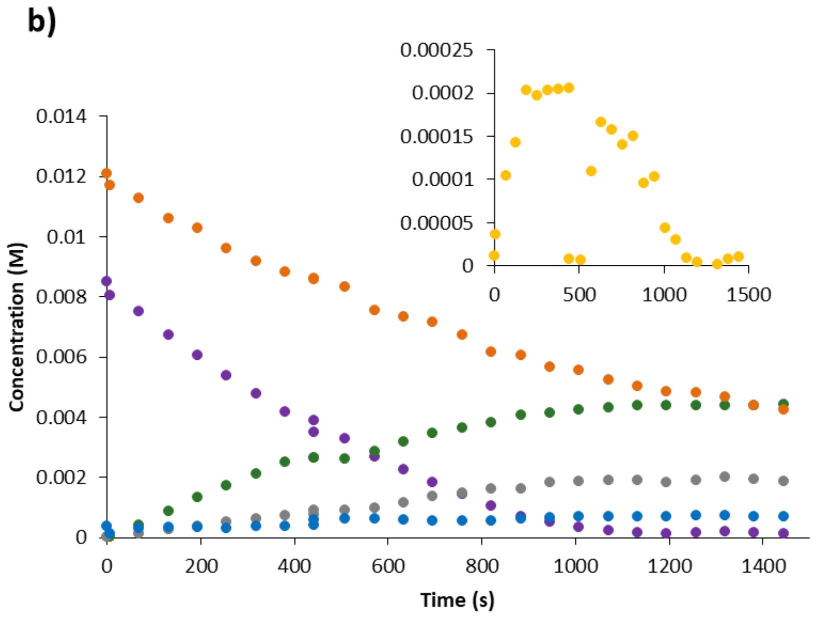

Figure S43. (**a**) Overlayed kinetic profiles of a reaction (hollow circles) (10 mM **1b**, 10 mM **2**, 0.25 mM $NiCl_2$(DME), 0.25 mM dtbbpy, 0.5 mM **6**, 10 mM $(TMS)_3SiH$, 20 mM 2,6-lutidine) run without disturbance and of an identical reaction run for 500 seconds before the reaction was paused by cessation of irradiation and tetrabutylammonium bromide was added into the NMR tube (complex **7** is depleted during the addition process). Overlap between the two reaction profiles clearly shows that exogenous Br^- has minimal impact on the reaction velocity and selectivity. (**b**) A reaction (12 mM **1b**, 8 mM **2**, 0.25 mM $NiCl_2$(DME), 0.25 mM dtbbpy, 0.5 mM **6**, 10 mM $(TMS)_3SiH$, 20 mM 2,6-lutidine) run for 420 seconds prior to being paused and lutidinium bromide being added into the NMR tube and the irradiation and monitoring then resumed (complex **7** is depleted during the addition process). It can be seen that the reaction continues on the previous trajectory indicating that addition of lutidinium bromide has minimal impact on the reaction velocity and selectivity.

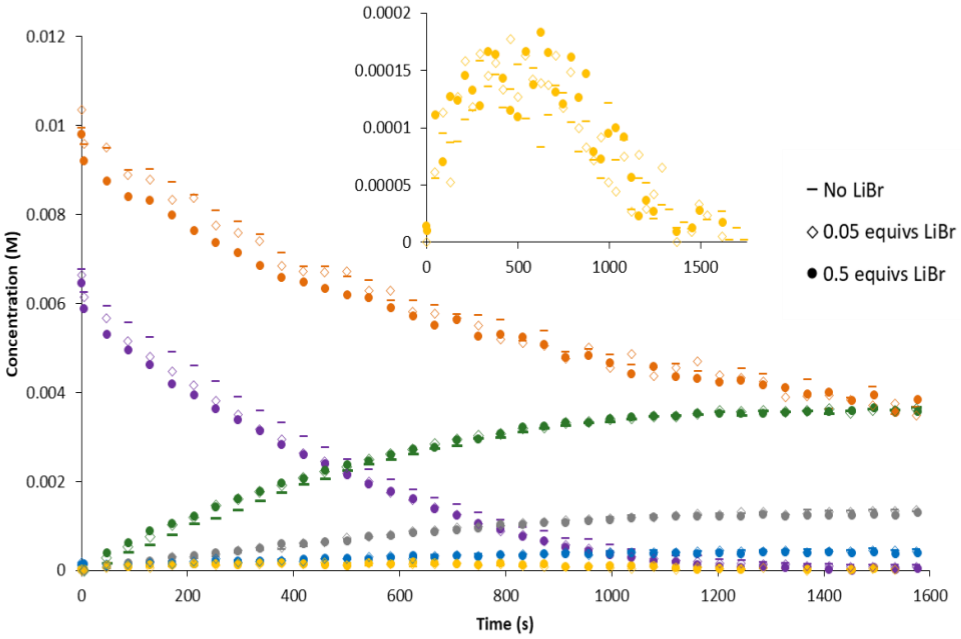

Figure S44. Overlayed kinetic profiles of three reactions with increasing amounts of LiBr being present in the solution for each reaction. Conditions: (7 mM **1b**, 10 mM **2**, 0.25 mM $NiCl_2$(DME), 0.25 mM dtbbpy, 0.5 mM **6**, 10 mM $(TMS)_3SiH$, 20 mM 2,6-lutidine; ‘1 equivalent’ of LiBr is 10 mM).

10. References

S1. Feldmeier, C.; Bartling, H.; Riedle, E.; Gschwind, R. M. LED Based NMR Illumination Device for Mechanistic Studies on Photochemical Reactions - Versatile and Simple, yet Surprisingly Powerful. *J. Magn. Reson*. **2013**, 232, 39–44.

S2. González-Bobes, F.; Fu, G. C. Amino Alcohols as Ligands for Nickel-Catalyzed Suzuki Reactions of Unactivated Alkyl Halides, Including Secondary Alkyl Chlorides, with Arylboronic Acids. *J. Am. Chem. Soc.* **2006**, 128, 5360–5361.

S3. Rosatella, A. A.; Afonso, C. A. M. Brønsted Acid-Catalyzed Dihydroxylation of Olefins in Aqueous Medium. *Adv. Synth. Catal.* **2011**, 353, 2920–2926.

S4. Pan, L.; Ke, Z.; Yeung, Y. Y. Lewis Base Catalyzed Dioxygenation of Olefins with Hypervalent Iodine Reagents. *Org. Lett.* **2021**, 23, 8174–8178.

S5. Colon, I.; Kelsey, D. R. Coupling of Aryl Chlorides by Nickel and Reducing Metals. *J. Org. Chem*. **1986**, 51, 2627–2637.

S6. Cheng, G.; Luo, M. Homocoupling of Arylboronic Acids Catalyzed by CuCl in Air at Room Temperature. *Eur. J. Org. Chem*. **2011**, 13, 2519–2523.

S7. Dai, Y.; Wu, F.; Zang, Z.; You, H.; Gong, H. Ni-Catalyzed Reductive Allylation of Unactivated Alkyl Halides with Allylic Carbonates. *Chem. Eur. J.* **2012**, 18, 808–812.

S8. Tredwell, M.; Preshlock, S. M.; Taylor, N. J.; Gruber, S.; Huiban, M.; Passchier, J.; Mercier, J.; Génicot, C.; Gouverneur, V. A General Copper-Mediated Nucleophilic 18F Fluorination of Arenes. *Angew. Chem. Int. Ed.* **2014**, 53, 7751–7755.

S9. Guo, L.; Tu, H. Y.; Zhu, S.; Chu, L. Selective, Intermolecular Alkylarylation of Alkenes via Photoredox/Nickel Dual Catalysis. *Org. Lett*. **2019**, 21, 4771–4776.

S10. Liang, Y.; Taya, A.; Zhao, Z.; Saito, N.; Shibata, N. Deoxyfluorination of Acyl Fluorides to Trifluoromethyl Compounds by FLUOLEAD/Olah's Reagent under Solvent-Free Conditions. *Beilstein J. Org. Chem*. **2020**, 16, 3052–3058.

S11. Sasaki, H.; Yokouchi, Y.; Nukazawa, T.; Iwamoto, T. Rapid and Mild Synthesis of an NHC-Coordinated Bis(Trimethylsilyl)Silylene via Elimination of Halotrimethylsilane. *Organometallics.* **2021**, 40, 2415–2419.

S12. Pickford, H. D.; Nugent, J.; Owen, B.; Mousseau, J. J.; Smith, R. C.; Anderson, E. A. Twofold Radical-Based Synthesis of N, C-Difunctionalized Bicyclo[1.1.1]Pentanes. *J. Am. Chem. Soc.* **2021**, 143, 9729–9736.

S13. Ben-Tal, Y.; Boaler, P. J.; Dale, H. J. A.; Dooley, R. E.; Fohn, N. A.; Gao, Y.; García-Domínguez, A.; Grant, K. M.; Hall, A. M. R.; Hayes, H. L. D.; Kucharski, M. M.; Wei, R.; Lloyd- Jones, G. C. Mechanistic Analysis by NMR Spectroscopy: A Users Guide. *Prog. Nucl. Magn. Reson. Spectrosc.* **2022**, *129*, 28–106.

S14. Ji, Y.; DiRocco, D. A.; Hong, C. M.; Wismer, M. K.; Reibarkh, M. Facile Quantum Yield Determination via NMR Actinometry*. Org. Lett.* **2018**, 20, 2156–2159.

S15. Demas, J. N.; Bowman, W. D.; Zalewski, E. F.; Velapoldi, R. A. Determination of the Quantum Yield of the Ferrioxalate Actinometer with Electrically Calibrated Radiometers. *J. Phys. Chem*. **1981**, 85, 2766–2771.

S16. Hatchard, C. G.; Parker, C. A. A New Sensitive Chemical Actinometer - II. Potassium Ferrioxalate as a Standard Chemical Actinometer. *Proc. R. Soc. London. Ser. A. Math. Phys. Sci.* **1956**, 235, 518–536.

S17. Harper, K. C.; Moschetta, E. G.; Bordawekar, S. V.; Wittenberger, S. J. A Laser Driven Flow Chemistry Platform for Scaling Photochemical Reactions with Visible Light. *ACS Cent. Sci*. **2019**, 5, 109–115.

S18. a) Diccianni, J. B.; Diao, T. Mechanisms of Nickel-Catalyzed Cross-Coupling Reactions. *Trends Chem.* **2019**, *1*, 830–844.; b) Devery, J. J.; Nguyen, J. D.; Dai, C.; Stephenson, C. R. J. Light-Mediated Reductive Debromination of Unactivated Alkyl and Aryl Bromides. *ACS Catal.* **2016**, *6*, 5962–5967.; c) Heitz, D. R.; Tellis, J. C.; Molander, G. A. Photochemical Nickel-Catalyzed C-H Arylation: Synthetic Scope and Mechanistic Investigations. *J. Am. Chem. Soc.* **2016**, *138*, 12715–12718.; d) Shields, B. J.; Doyle, A. G. Direct C(Sp3)-H Cross Coupling Enabled by Catalytic Generation of Chlorine Radicals. *J. Am. Chem. Soc.* **2016**, *138*, 12719–12722.; e) Santos, M. S.; Corrêa, A. G.; Paixão, M. W.; König, B. C(Sp3)−C(Sp3) Cross-Coupling of Alkyl Bromides and Ethers Mediated by Metal and Visible Light Photoredox Catalysis. *Adv. Synth. Catal.* **2020**, *362*, 2367–2372.; f) Sun, R.; Qin, Y.; Nocera, D. G. General Paradigm in Photoredox Nickel-Catalyzed Cross-Coupling Allows for Light-Free Access to Reactivity. *Angew. Chem. Int. Ed.* **2020**, *59*, 9527–9533.; g) Cavallo, L.; Rueping, M.; Maity, B.; Zhu, C.; Yue, H.; Huang, L.; Harb, M.; Minenkov, Y. Mechanistic Insight into the Photoredox-Nickel-HAT Triple Catalyzed Arylation and Alkylation of α-Amino Csp3-H Bonds. *J. Am. Chem. Soc.* **2020**, *142*, 16942–16952.; h) Gutierrez, O.; Tellis, J. C.; Primer, D. N.; Molander, G. A.; Kozlowski, M. C. Nickel-Catalyzed Cross-Coupling of Photoredox-Generated Radicals: Uncovering a General Manifold for Stereoconvergence in Nickel-Catalyzed Cross-Couplings. *J. Am. Chem. Soc.* **2015**, *137*, 4896–4899.; i) Zhang, P.; Le, C. C.; MacMillan, D. W. C. Silyl Radical Activation of Alkyl Halides in Metallaphotoredox Catalysis: A Unique Pathway for Cross-Electrophile Coupling. *J. Am. Chem. Soc.* **2016**, *138*, 8084–8087.; j) Sakai, H. A.; Liu, W.; Le, C.; MacMillan, D. W. C. Cross-Electrophile Coupling of Unactivated Alkyl Chlorides. *J. Am. Chem. Soc.* **2020**, *142*, 11691–11697.; k) Huang, H. M.; Bellotti, P.; Erchinger, J. E.; Paulisch, T. O.; Glorius, F. Radical Carbonyl Umpolung Arylation via Dual Nickel Catalysis. *J. Am. Chem. Soc.* **2022**, *144,* 1899–1909.; l) Paul, A.; Smith, M. D.; Vannucci, A. K. Photoredox-Assisted Reductive Cross-Coupling: Mechanistic Insight into Catalytic Aryl-Alkyl Cross-Couplings. *J. Org. Chem.* **2017**, *82*, 1996–2003.

S19. Tian, L.; Till, N. A.; Kudisch, B.; MacMillan, D. W. C.; Scholes, G. D. Transient Absorption Spectroscopy Offers Mechanistic Insights for an Iridium/Nickel-Catalyzed C-O Coupling. *J. Am. Chem. Soc*. **2020**, 142, 4555–4559.

S20. Lowry, M. S.; Goldsmith, J. I.; Slinker, J. D.; Rohl, R.; Pascal, R. A.; Malliaras, G. G.; Bernhard, S. Single-Layer Electroluminescent Devices and Photoinduced Hydrogen Production from an Ionic Iridium(III) Complex. *Chem. Mater.* **2005**, 17, 5712-5719.

S21. a) Farney, E. P.; Chapman, S. J.; Swords, W. B.; Torelli, M. D.; Hamers, R. J.; Yoon, T. P. Discovery and Elucidation of Counteranion Dependence in Photoredox Catalysis. *J. Am. Chem. Soc.* **2019**, 141, 6385–6391.; b) Earley, J. D.; Zieleniewska, A.; Ripberger, H. H.; Shin, N. Y.; Lazorski, M. S.; Mast, Z. J.; Sayre, H. J.; McCusker, J. K.; Scholes, G. D.; Knowles, R. R.; Reid, O. G.; Rumbles, G. Ion-Pair Reorganization Regulates Reactivity in Photoredox Catalysts. *Nat. Chem.* **2022**.; c) Ilic, S.; Cairnie, D. R.; Bridgewater, C. M.; Morris, A. J. Investigation into Dual

Emission of a Cyclometalated Iridium Complex: The Role of Ion-Pairing. *J. Photochem. Photobiol.* **2021**, *8*, 100084.
